# Granulocyte differentiation arrest in HAX1-deficient cells, demonstrated in a new in vitro model of a certain phenotypic aspects of Kostmann disease, is caused by ineffective lipid droplet autophagy and fatty acids uptake

**DOI:** 10.1038/s41419-026-08805-y

**Published:** 2026-05-05

**Authors:** Maciej Wakula, Milena Jablonowska, Mateusz Chmielarczyk, Leszek Tarnowski, Mariusz Kulinczak, Ewa Sitkiewicz, Bianka Swiderska, Emilia Samborowska, Mariusz Radkiewicz, Mostafa Kianfar, Malgorzata Statkiewicz, Izabela Rumienczyk, Anna Balcerak, Ryszard Konopinski, Alicja Trebinska-Stryjewska, Ewa A. Grzybowska

**Affiliations:** 1 Maria Sklodowska-Curie National Research Institute of Oncology, Warsaw, Poland; 2 Institute of Biochemistry and Biophysics, Polish Academy of Sciences, Warsaw, Poland; 3 Military University of Technology, Warsaw, Poland

**Keywords:** Cell biology, Mechanisms of disease

## Abstract

Molecular mechanisms underlying congenital neutropenia in patients with HAX1 deficiency are not clear at the moment. HAX1 deficiency was shown to result in the arrest of neutrophil differentiation. Our studies of the effect of HAX1 deficiency on the proteomic and metabolic profiles of promyelocytic cells have led to the conclusion, supported by specific tests, that fatty acid metabolism is affected in *HAX1* KO cells. The lipid droplet content is increased in *HAX1* KO cells, pointing to the accumulation of fatty acids that are not metabolized. Studies of autophagosome function in *HAX1* WT and KO cells revealed that lipid droplet autophagy is defective at the stage of fusion with the lysosome. Autophagy-dependent generation of free fatty acids is critical for neutrophil differentiation, so HAX1 deficiency that affects normal autophagy of lipids in promyeloblasts should explain differentiation arrest. Moreover, we have demonstrated that HAX1-deficient cells are also compromised in fatty acid uptake.

## Introduction

Neutrophils are the most abundant immune cells that represent the first line of defense against pathogens. They are short-lived and constantly produced in the bone marrow (BM) in a tightly regulated and energy-consuming process of granulopoiesis [1]. During granulopoiesis, granulocyte-monocyte progenitor cells (GMPs) develop into mature neutrophils in a series of specific steps, requiring extensive morphological and metabolic changes. Molecular mechanisms governing granulocyte differentiation are highly coordinated, and accompanied by a massive change in gene expression that affects at least one-third of all currently annotated genes [2]. Much of this regulation has been explained, but some of its aspects remain unknown, including the exact role of the HAX1 protein in granulocyte differentiation. Riffelmacher et al. [3] demonstrated that inhibition of autophagy-mediated lipid degradation or fatty acid oxidation was sufficient to cause defective mitochondrial respiration and defective differentiation. Here, we show that HAX1 is involved in the regulation of these processes, which should contribute to clarification of its role in neutrophil differentiation.

HAX1 protein (HCLS1-associated protein X) was originally described as a protein partner of the hematopoietic-specific protein HS1 in B cells [4]. Subsequent studies revealed that HAX1 is ubiquitously expressed and involved in many cellular processes, including apoptosis, cell migration, regulation of calcium homeostasis, mitochondrial homeostasis, autophagy, angiogenesis and translation [5, 6].

HAX1 deficiency has been shown to cause autosomal, recessive severe congenital neutropenia (SCN) [7], but, despite several attempts to clarify the underlying mechanism, to the moment it is not fully clear. An initial report suggested that HAX1 plays a role in maintaining the inner mitochondrial membrane potential, protecting myeloid cells from apoptosis [7]. Later, it was proposed that protein HCLS1 is activated by HAX1, and translocates to the nucleus, where it induces transcription of target genes of the transcription factor LEF1 (lymphoid-enhancer binding factor 1), which is essential for granulocyte colony-stimulating factor (G-CSF)–triggered granulopoiesis [8]. Recently, Fan et al. [9] suggested that HAX1 regulates mitochondrial proteostasis as a more probable molecular mechanism governing granulocyte differentiation. The authors proposed that the interaction of HAX1 with mitochondrial chaperone CLPB is crucial to maintaining mitochondrial proteostasis, which is critical to its functioning. The authors demonstrated that impaired mitochondrial metabolism, caused by HAX1 deficiency, affects neutrophil differentiation and function through a functional link with PRKD2 kinase and the small heat shock protein HSP27. This report is in line with some previous data, which highlighted the importance of functional mitochondria in maintaining hematopoietic stem cell function [10] and proper differentiation [11, 12].

Here, we present a different approach, which does not exclude these previous findings, but adds to a better understanding of the HAX1 role. Quantitative comparison of *HAX1* WT and *HAX1* KO proteomes in promyelocytic leukemia cells had led us to the conclusion that HAX1-deficient cells are affected in lipid metabolism. We verified and confirmed the differences between these cells, using specific metabolic assays and estimating the accumulation of lipid droplets (LDs). Subsequently, we established that lipid droplet accumulation detected in *HAX1* KO cells is caused by impaired autophagy of LDs, and that this process is affected in the late phase, in which the fusion with the lysosome takes place. Inhibition of LD degradation prevents the release of the free fatty acids, which impairs β-oxidation and energy production, required for differentiation. To further validate these findings, we have established a new, HL60-based model of HAX1-dependent differentiation arrest (functional model of Kostmann’s disease) and demonstrated that fatty acids are utilized differently in *HAX1* KO cells undergoing differentiation, which is also reflected in the rate of cellular ATP production. Additionally, we have observed for the first time, that HAX1 is involved in the regulation of malate-aspartate shuttle.

## Results

### Differences in proteomic profile between HAX1 WT and HAX1-deficient cells (iTRAQ)

In our previous report, we evaluated changes in the mRNA expression profile caused by *HAX1* knockout in the HL-60 cell line, finding that differentially expressed genes belong to GO terms involved in the processing of rRNA, ribosome biogenesis, translation, and mitochondrial respiration [13]. However, changes at the mRNA level may not directly translate into the protein level; therefore, we performed quantitative iTRAQ mass spectrometry analysis of *HAX1* WT and KO cells (Supplementary File S1, results in database: ProteomeXchange PXD064511). The principal component analysis (PCA) of the iTRAQ data shows a good separation of conditions for the *HAX1* WT and *HAX1* KO cell lines (Supplementary Fig. S1). Differences between datasets were analyzed using standard tools (GO Biological Process and Pathway analyzes), but also using QSM (Quantitative System Metabolism) platform, to evaluate metabolic changes at a deeper level.

#### Differential proteomic analysis indicates differences in amino acid metabolism, neutrophil degranulation and signaling by Rho GTP-ases

**Fig. 1:**
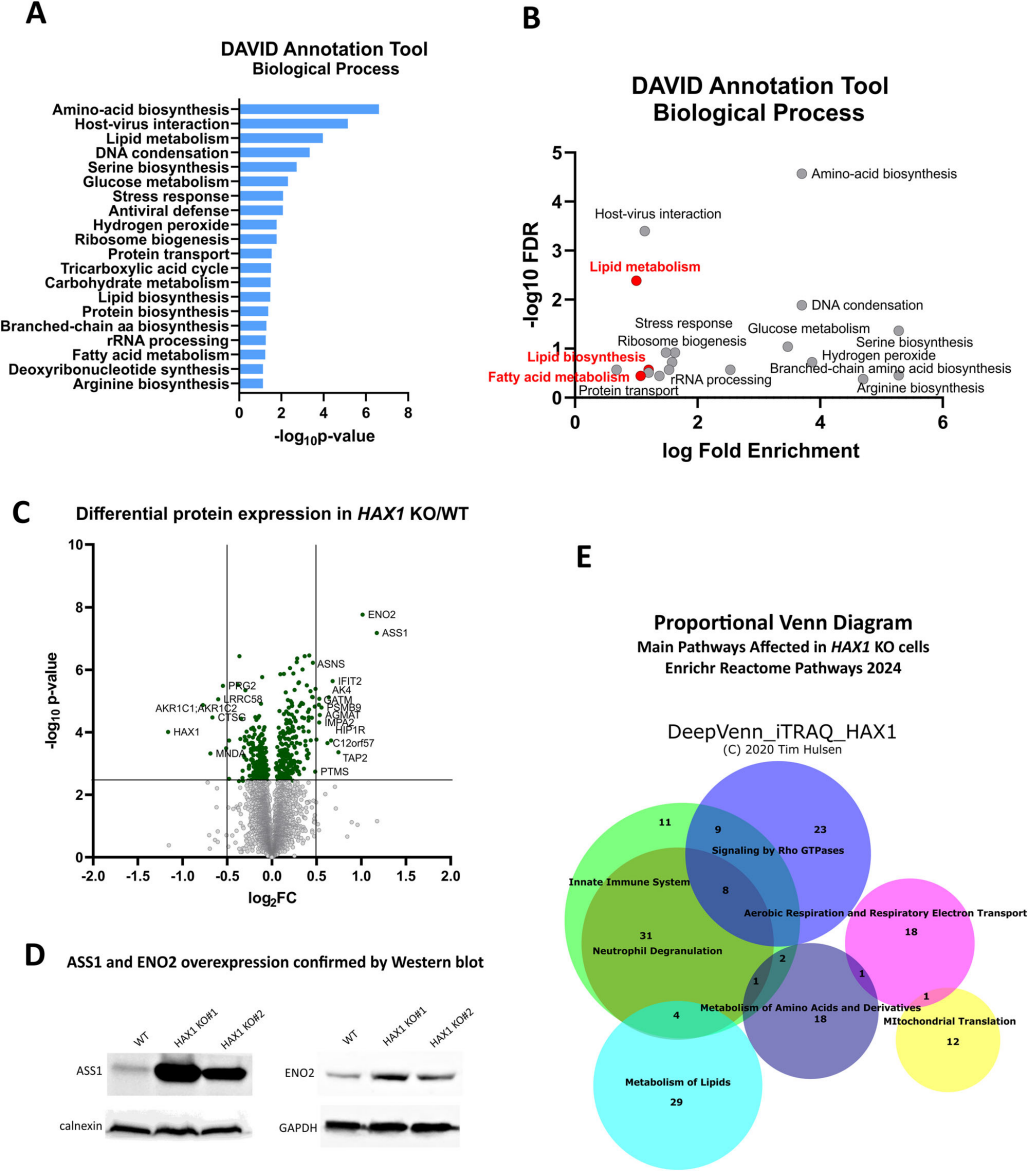
Quantitative proteomic data analysis reveals that HAX1-deficient cells are affected in amino acid metabolism, neutrophil degranulation, signaling by Rho GTP-ases, translation, energy metabolism and lipid metabolism. **A** Terms of biological process differentially regulated in HAX1-deficient cells. iTRAQ data analyzed by DAVID Functional Annotation Tool. Ranked by –log_10_(*p*-value). **B** Biological Process enrichment analysis (DAVID). Fold Enrichment plotted against False Discovery Rate. Categories linked to lipid metabolism marked in red. For clarity, some labels are omitted. **C** Differential protein expression scatter plot for *HAX1* KO(#1,#2)/WT cells. **D** ASS1 and ENO2 upregulation in *HAX1* KOs confirmed by Western blot. **E** Proportional Venn diagrams showing main pathways affected in HAX1-deficient cells. iTRAQ data analyzed using Enrichr. The overlap between categories is total with innate immunity and neutrophil degranulation and moderate with neutrophil degranulation and Rho GTPases activity, in other cases the overlap is minimal.

Quantitative mass spectrometry results of HL60 *HAX1* WT and *HAX1* KO#2 cells (Supplementary Fig. S2, cell line generation described in ref. [13]) were analyzed with standard ontology and pathway tools (DAVID Annotation tool, Enrichr, Supplementary File S2). The analysis of the terms of Biological Process points to differences in amino-acid biosynthesis, but also indicates differences in lipid metabolism, DNA condensation, ribosome biogenesis and energy metabolism (Fig. 1A, B). The differential protein plot (Fig. 1C) shows the differences in protein expression between *HAX1* KO/WT cells and indicates that the two proteins most upregulated in *HAX1* KO cells are argininosuccinate synthase (ASS1) and gamma-enolase (ENO2). Upregulation of both proteins was confirmed by Western blot (Fig. 1D, uncropped image in Supplementary Material). ASS1 is an enzyme of the arginine biosynthesis pathway, which is in line with the results of the biological process analysis performed for iTRAQ data, showing that arginine biosynthesis is one of the most affected pathways. ENO2 is an enzyme involved in glycolysis and gluconeogenesis, pointing to differences in energy metabolism.

Analysis of specific pathways (Reactome) affected by HAX1 deficiency revealed several main categories: proteins involved in neutrophil degranulation, partially overlapping with signaling by RhoGTPases, proteins involved in amino acid metabolism, respiratory electron transport, mitochondrial translation, and lipid metabolism (Fig. 1E, Supplementary Fig. S3, Supplementary File S2).

#### Bioanalysis of central metabolism performed using the QSM platform indicates differences in fatty acid metabolism and general energy metabolism in *HAX1* KO cells

Changes in proteome detected using standard pathway analysis indicated the possibility of HAX1 impact on cell metabolism, but QSM™ (Quantitative System Metabolism) data analysis represents a better and more precise tool to characterize metabolic changes. QSM data analysis uses quantitative proteomic information on the expression levels of enzymes to determine metabolic profiles, states, capacities and metabolic fluxes. iTRAQ data passed the quality control (QC) score and the QSM core was used to classify the suitability of proteomic data for QSM analysis (both above 100%, Doppelganger Biosystem Report, Supplementary File S3). The model includes not only the assessment of maximal metabolic capacities but also a more complex assessment of metabolic functions under physiological conditions in which concentrations are not independent of each other. It also provides a panel of significantly regulated functional markers.

**Fig. 2:**
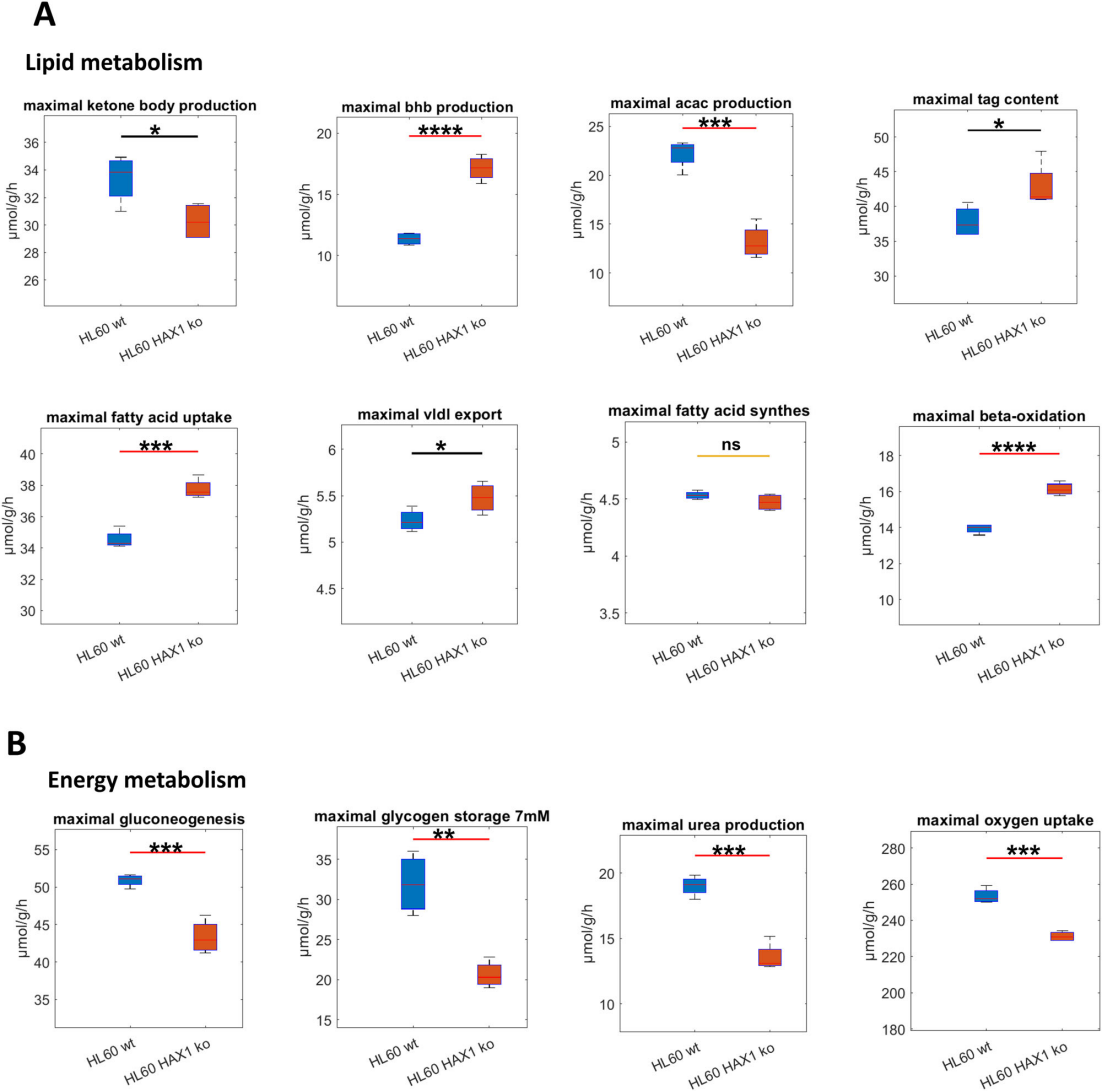
QSM (Quantitative System Metabolism) analysis of iTRAQ data shifts focus to lipid metabolism and energy metabolism. **A** Categories of lipid metabolism significantly changed in QSM analysis. **B** Categories of energy metabolism significantly changed in QSM analysis. Energy metabolism was highlighted as a second most changed category.

The analysis revealed pronounced shifts in metabolic capacities in *HAX1*-KO cells, most notably in fatty-acid metabolism. Key fatty-acid–related functions altered in *HAX1*-KO cells are shown in Fig. 2A and include capacities for ketone-body production, triacylglycerol (TAG) content, fatty-acid synthesis and uptake, VLDL secretion, and β-oxidation. The second most changed category in QSM analysis (after lipid metabolism) was energy metabolism. Statistically significant changes were also detected in carbohydrate and amino-acid metabolism. Affected processes include gluconeogenesis, urea production, glycogen storage, and oxygen consumption capacity, indicating broader alterations in central metabolism (Fig. 2B). A proportional Venn diagram summarizes significantly regulated functional protein markers by pathway membership (Supplementary Fig. S3). Plots of linear-regression relationships between protein abundances and metabolic capacities for all specific markers are provided in the supplement (Doppelganger Biosystems Data Analysis Report, Supplementary File S3, statistical details: Supplementary File S4). Consistent with the functional results, most markers map to fatty-acid utilization.

### Differences in the metabolic profile of fatty acids established by MS LC indicate increased accumulation in *HAX1* KO cells

**Fig. 3:**
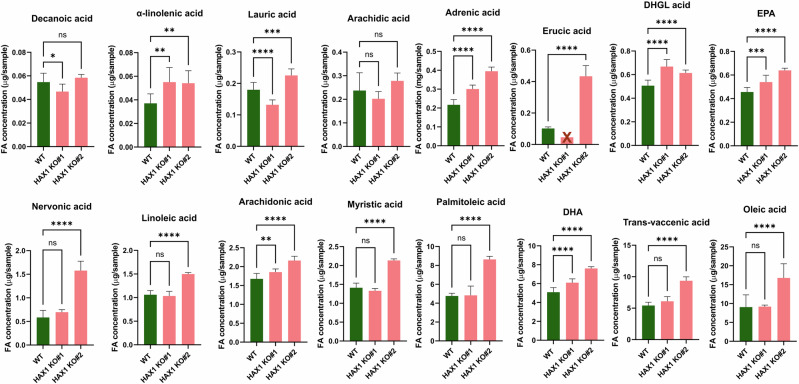
Metabolic mass spectrometry analysis of a panel of fatty acids in *HAX1* WT/KO cells. A comparison of fatty acid content in *HAX1* WT/KO(#1 and #2) cells. From the 18 FAs in the panel, 16 with a detectable signal are shown. FAs concentration is presented as μg/sample of 8 × 10^6^ cells. Each bar represents (in most cases) a mean of *n* = 9 measurements from 3 biological replicates. Statistical significance assessed by One-way ANOVA and Tukey, all statistical details and specific *p*-values at Supplementary Table S1.

QSM data analysis of the iTRAQ data pointed clearly to differences in lipid metabolism and fatty acid utilization. To verify this results, the fatty acid content in *HAX1* WT and KO (#1 and #2) cells was analyzed by mass spectrometry for the specific fatty acid panel. The results reveal a significantly higher concentration of most FAs in *HAX1*-deficient cells, especially for *HAX1* KO#2, which is more stringent (Fig. 3, Supplementary File S5). The results indicate the possibility that fatty acids accumulate in *HAX1* KO cells in the form of lipid droplets.

### Specific metabolic tests reveal that HAX1 deficiency results in significant metabolic changes, including differences in fatty acid metabolism and levels of urea, aspartate, and lactate

**Fig. 4:**
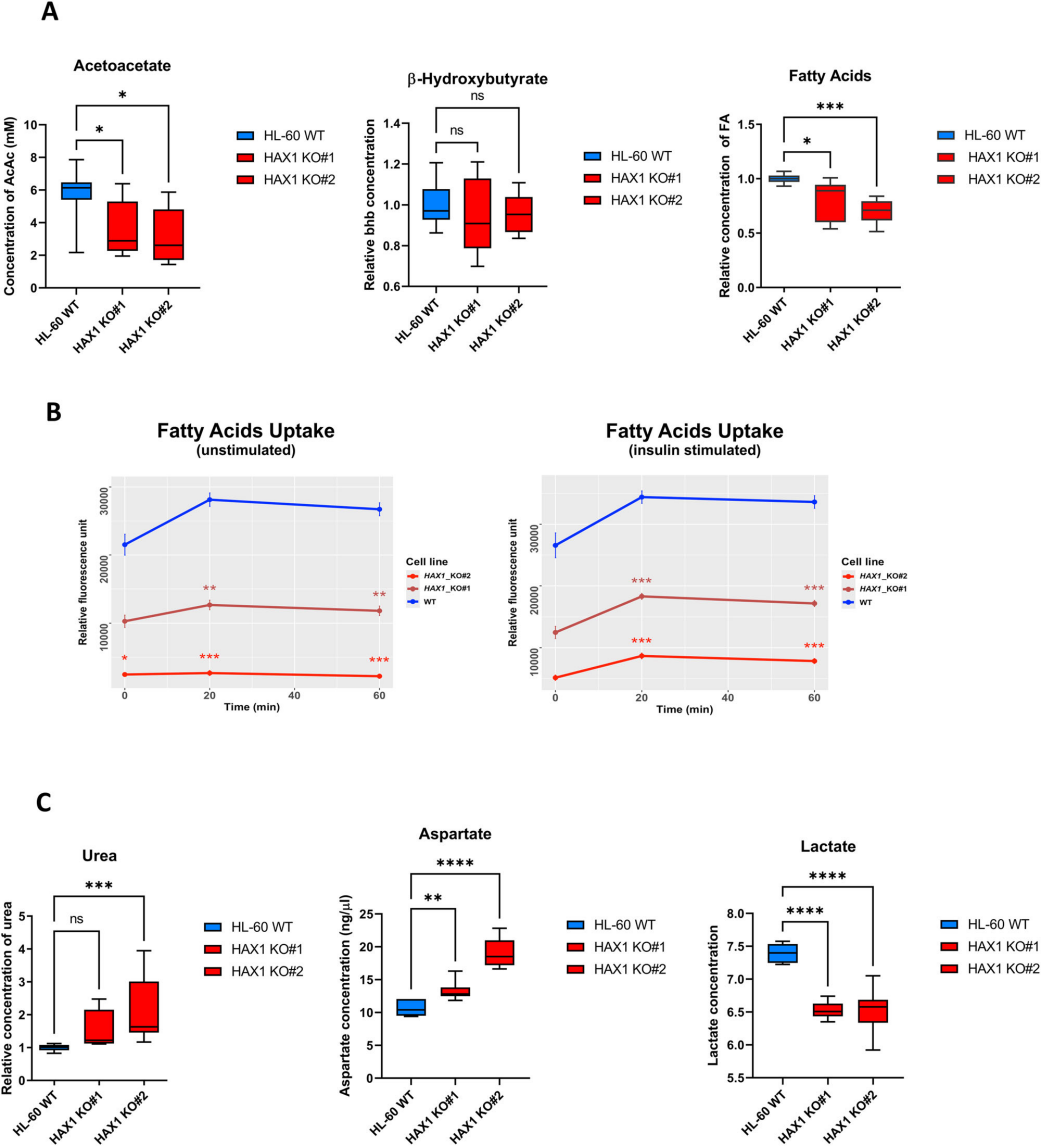
Direct metabolic assays confirm that HAX1 deficiency affects fatty acid metabolism, energy metabolism and the urea cycle. **A** Metabolic assays for ketone bodies and fatty acids. HAX1 deficiency significantly affects ketone bodies (acetoacetate, β-hydroxybutyrate) and fatty acid content, with lower levels in *HAX1* KO cells. Statistical analysis: One-way ANOVA and post-hoc Tukey, *n* = 6–9, 3 biological replicates, statistical details: Supplementary Table S1. **B** Fatty acids uptake assays. The rate of fatty acid uptake is significantly lower in HAX1-deficient cells, both unstimulated and after insulin treatment. Statistical analysis: Wilcoxon rank sum test, statistical details: Supplementary Table S2. **C** Urea-cycle related assays. HAX1-deficient cells display significantly higher levels of urea and aspartate and lower levels of lactate, which is consistent with upregulated malate-aspartate shuttle. Statistical analysis: One-way ANOVA and post-hoc Tukey, *n* = 7–12, 3 biological replicates, statistical details: Supplementary Table S1.

To verify and evaluate possible significant changes in fatty acid metabolism in *HAX1* KO cells, the levels of ketone bodies and free fatty acids were assessed by specific tests. The results confirmed a decrease in acetoacetate levels in *HAX1* KO, as predicted by the model, while the difference in β-hydroxybutyrate levels was not significant (Fig. 4A). Free fatty acid levels were reduced in *HAX1* KO cells, as well as FA uptake levels, both unstimulated and stimulated by insulin (Fig. 4A, B). Although metabolic tests in general confirmed significant differences in fatty acid metabolism between *HAX1* WT and KO cells, FAs uptake results contradict model predictions.

Furthermore, specific tests revealed significant differences between *HAX1* WT and *HAX1* KO cells in urea, aspartate and lactate, pointing to metabolic changes that affect energy production and amino acid biosynthesis. Urea levels are increased in *HAX1* KO cells, contradicting the predictions of the model, while changes in aspartate and lactate levels in *HAX1* KO (increased and decreased, respectively) may be related to the functioning of the malate-aspartate shuttle (MAS).

### HAX1 deficiency affects MAS cycle by blocking aspartate-glutamate carrier (AGC)

MAS transports reducing equivalents across the inner mitochondrial membrane generating NADH in the mitochondrial matrix and recycling cytosolic NADH to NAD+. HAX1 interactions with shuttle proteins SLC25A13, SLC25A12, and SLC25A11 have already been reported by our group [14]. These proteins belong to a family of mitochondrial carriers (solute carrier family 25 members) and execute an exchange of amino acids across the inner mitochondrial membrane. SLC25A13 and SLC25A12 (aspartate-glutamate carriers: Citrin/Aralar) represent homologs that exchange aspartate for glutamate and a proton. SLC125A11 transports 2-oxoglutarate across the inner membrane in an electroneutral exchange for malate. MAS deficiency is known to affect serine biosynthesis, aspartate levels and increase the level of lactate [15]. Our analysis revealed differences in serine and arginine biosynthesis (Fig. 1A, B, D), an increase in the level of aspartate and a decrease in the level of lactate in *HAX1* KO cells (Fig. 4C), suggesting that HAX1 binding inhibits the carriers, however a direct evidence is still lacking. Aspartate exported by citrin is converted to argininosuccinate by argininosuccinate synthase (ASS1), which was found to be upregulated in *HAX1* KOs in mass spectrometry analysis and Western blot (Fig. 1C, D, uncropped image in Supplementary Material).

**Fig. 5:**
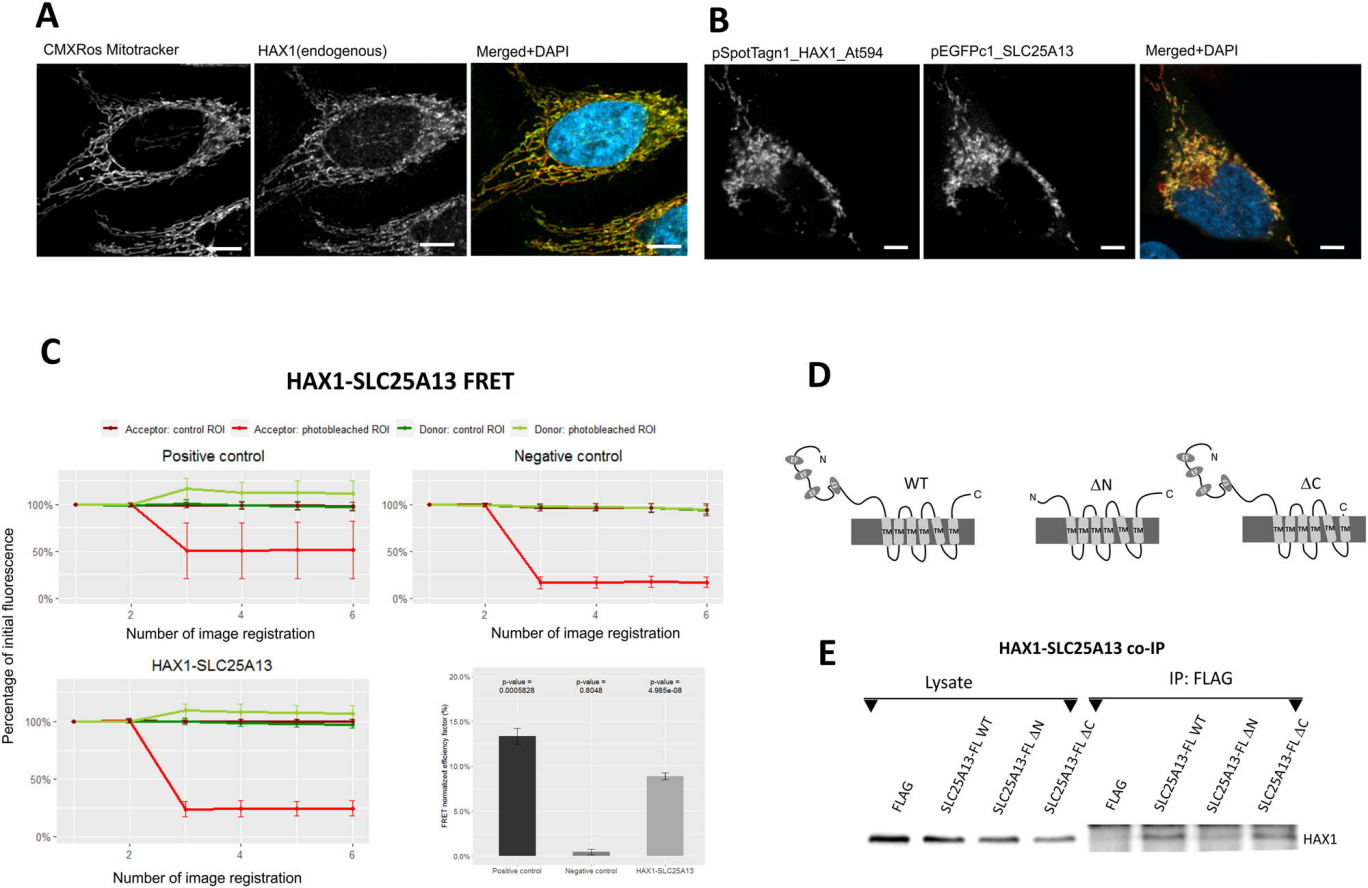
HAX1 interacts with N-terminal part of a malate-aspartate shuttle component SLC25A13. **A** Co-localization of HAX1 with CMXRos Mitotracker. Endogenous HAX1 detected with anti-HAX1 antibody (11266-1-AP, Proteintech), HeLa cells, scale bar: 10 μm. **B** Co-localization of HAX1 with mitochondrial transporter SLC25A13. Configuration used for FRET (pSpotTagn1_HAX1_ATTO594 / pEGFPc1_SLC25A13), HeLa cells, scale bar: 5 μm. **C** FRET signal quantification. Positive control: : pMitoEGFPc1_SpotTagn1_ATTO594, negative control: pSpotTagn1HAX1 / pMitoEGFPc1. Bar plot shows signal quantification. **D** Representation of the placement of SLC25A13 transporter and its deletion mutants (ΔN and ΔC)in relation to the mitochondrial inner membrane. **E** Co-immunoprecipitation of HAX1 with SLC25A13 and its mutants indicates interaction on N-terminus of SLC25A13. Detection: anti-HAX1 antibody (11266-1-AP, Proteintech).

The interaction of HAX1 with the aspartate-glutamate carrier SLC25A13 (citrin) was confirmed and quantified by FRET analysis of cells co-transfected with EGFP-SLC25A13 and HAX1-SpotTag/ATTO594 (one step antibody), co-localizing in mitochondria (Fig. 5A, B). The interaction was also analyzed by co-immunoprecipitation with the intact protein and its truncated isoforms, which lacked the N-terminal part (containing EF-hand motifs, ΔN) and the C-terminal part (ΔC) (Fig. 5C, D). The results indicate a weaker interaction with the ΔΝ construct, which contains a calcium-dependent regulatory region.

### Autophagy of lipid droplets is altered in *HAX1* KO cells at the stage of lysosome fusion

**Fig. 6:**
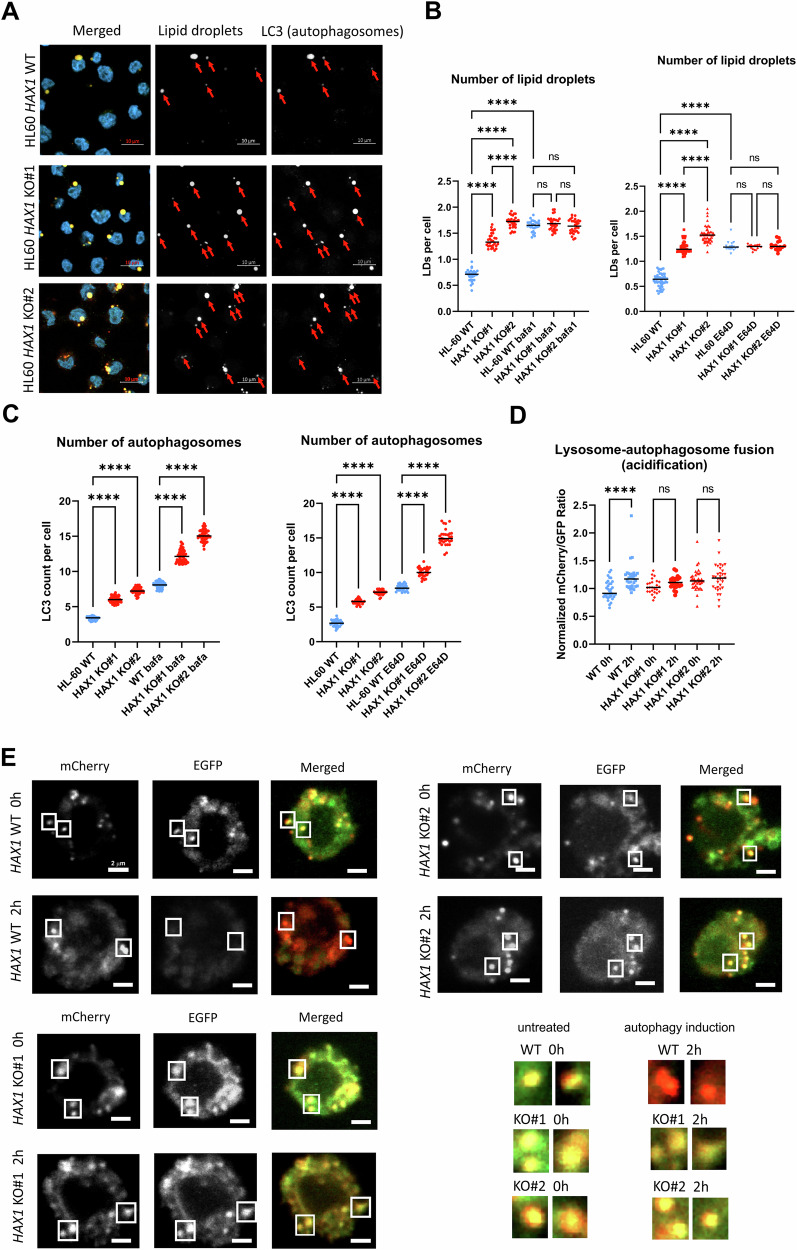
HAX1-deficient cells are blocked in lipid droplet autophagy at the stage of the fusion with lysosomes. **A** Representative images of *HAX1* WT and *HAX1* KO cells showing co-localization of lipid droplets (red) and LC3 (green) staining. LDs and LC3 marked by red arrows. **B** Lipid droplet quantification. The number of lipid droplets per cell is significantly higher in HAX1-deficient cells. The inhibitors of late autophagy (bafilomycin and E64D) cause the increase of LD numbers in the wild type, while the number of LDs in *HAX1* KO cells is unchanged. Statistical analysis: One-way ANOVA and Tukey, *n* = 16–52, biological replicates: 3–8, statistical details: Supplementary Tables S1, S3. **C** Autophagosome quantification. The number of autophagosomes (LC3A/B foci count) per cell is significantly higher in *HAX1* KO cells and this effect remains after the treatment with late autophagy inhibitors (bafilomycin and E64D). Statistical analysis: One-way ANOVA and Tukey, n = 30–82, biological replicates: 5–13, statistical details: Supplementary Tables S1, S3. **D**, **E** Autophagosome acidification assay. Autophagosome acidification is significant after the induction of autophagy by glucose starvation only in *HAX1* WT, but not in *HAX1* KO cells. **D** Quantification of the effect by establishing mCherry/GFP ratio using GFP-mCherry-LC3 vector, calculation from separate channels, statistical analysis One-way ANOVA and Tukey, *n* = 25–36, biological replicates: 2–3, statistical details: Supplementary Tables S1, S3. **E** Representative images of autophagosome acidification after 2 h glucose starvation. Insets: two randomly selected regions with autophagosomes from larger images.

Differences in fatty acid metabolism detected in iTRAQ analysis, metabolic mass spectrometry analysis and subsequent metabolic tests suggested that HAX1-deficient cells may exhibit differences in lipid droplet accumulation and processing. Autophagy of lipid droplets is a key process during neutrophil maturation, providing free fatty acids for β-oxidation and energy production [3]. To examine the differences in lipid droplets accumulation and autophagy, the number of LDs and autophagosomes was assessed by immunofluorescence. Lipid droplets were stained with Droplite™ Red and counted in *HAX1* WT and *HAX1* KO cells (#1 and #2). The results show a significantly higher number of lipid droplets (LDs) in HAX1-deficient cells (Fig. 6A, B), indicating their abnormal accumulation. Interestingly, after treatment with autophagy inhibitors, bafilomycin A1 and E64D, the difference in the number of lipid droplets disappears (Fig. 6B), reaching the same amount in *HAX1* WT as in *HAX1* KOs (with and without treatment), indicating that the autophagy of LDs is disrupted in *HAX1* KO cells. The number of autophagosomes, calculated from the staining with the LC3 autophagy marker, is significantly higher in *HAX1* KO cells, which may be a simple consequence of the higher number of LDs in these cells (Fig. 6A, C). Upon treatment with the same autophagy inhibitors, the number of autophagosomes increases proportionally in *HAX1* WT and *HAX1* KO, indicating that at that level both types of cells react in the same way (Fig. 6C). This is expected, because both autophagy inhibitors used in these experiments act in late stages and do not interfere with autophagosome formation. These results suggest that LD autophagy is blocked at the later stage of fusion with the lysosome.

To verify this hypothesis, we used the FUW mCherry-GFP-LC3 construct [16] which enables visualization of free autophagosomes (GFP and mCherry fluorescence, yellow) and autophagosomes that have fused with the lysosome (autolysosomes; mCherry fluorescence only, due to the acid sensitivity of GFP). We have calculated the mCherry/GFP ratio in *HAX1* WT and *HAX1* KO cells at time 0 and after 2 h of induction of autophagy by glucose starvation. The results clearly show that while in *HAX1* WT there is a significant difference in the mCherry/GFP ratio before and after autophagy induction, indicating the disappearance of the GFP signal due to acidification, in *HAX1* KOs (#1 and #2), glucose starvation does not induce changes in the ratio (Fig. 5D, E). This indicates that in *HAX1* KO cells the fusion of autophagosome with lysosome is inhibited, resulting in the accumulation of LD and preventing proper degradation of LDs to free FAs.

### Deficiency in *HAX1* KO cell differentiation demonstrated in a new, HL-60-based model reproducing certain aspects of the phenotype of Kostmann disease

**Fig. 7:**
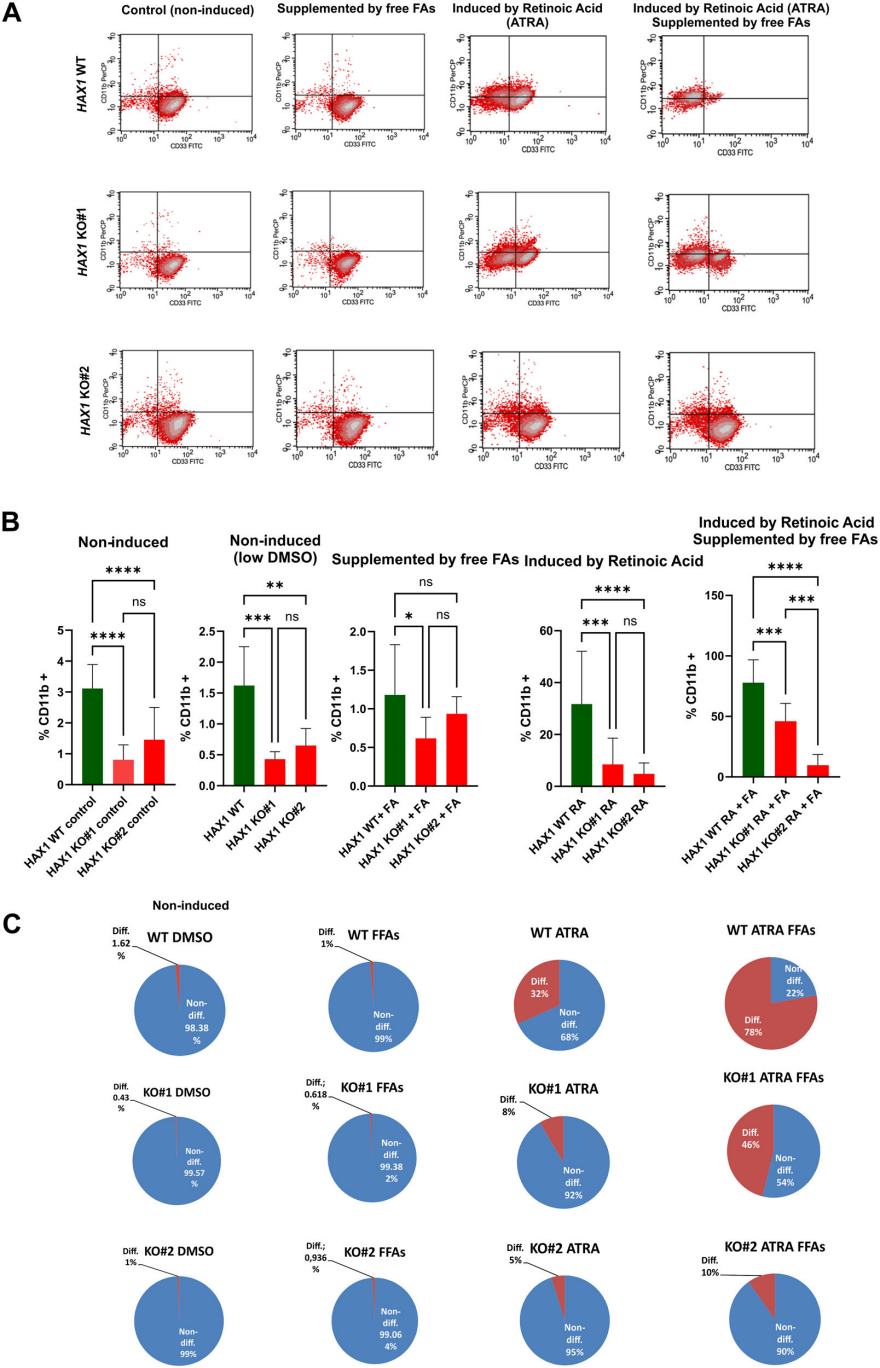
Differentiation of HL-60 WT and HL-60 *HAX1* KO cells in presence of ATRA and ATRA/FAs. **A** Differentiation assessed by CD11b expression. Flow cytometry results showing CD11b and CD33 expression in HL-60 *HAX1* WT /KO cells, non-induced (with control DMSO in low concentration as in ATRA and FAs mix) and induced to differentiation after 72 h incubation with 58 μM ATRA and ATRA+FAs mix. Quadrants illustrate differences in marker expression of differentiating neutrophils in HL-60 variants. Experiment was conducted in three independent replicates. Representative data are shown. Gating strategy is described in Supplementary Fig. S5. **B** Quantification of CD11b expression in HL-60 *HAX1* WT and HL-60 *HAX1* KO cells in all studied conditions. Statistical analysis: One-way ANOVA, Tukey, *n* = 5–18, biological replicates: 1(undifferentiated control)-5, statistical details: Supplementary Table S1. **C** Pie charts showing the proportion of differentiating HL-60 *HAX1* WT and HL-60 *HAX1* KO cells in all conditions (excluding non-induced cells without traces of DMSO, which was the same as non-induced cells with low DMSO).

To date, the effect of HAX1 on neutrophil differentiation has been demonstrated by analysis of patient neutrophil mutations and in an in vitro differentiation system of induced pluripotent stem cell (iPSC) [9, 17]. We used a much simpler approach in which we induced the differentiation of the promyelocytic HL-60 cell line by retinoic acid (ATRA), as described [18] and used our WT control and *HAX1* KO cell lines to estimate the differentiation rate in both types of cells. As expected, we observed a lower ratio of differentiated cells in *HAX1* KO cells (Fig. 7). CD11b was used as a marker for differentiation, as a specific surface marker of neutrophils. CD11b was chosen, because it is the most common marker used in the majority of analyzes, and it appears at the myelocyte stage. CD33 was used as a reference marker. CD33 is present in initial myeloblasts and its expression declines during differentiation. In non-induced cells the number of CD11b positive cells is minimal, compared to the total, but it is statistically lower in *HAX1* KO cells (up to 3X lower, Fig. 7A, B). After induction with retinoic acid (58 μM ATRA), WT cells display differentiation up to 32%, while in *HAX1* KO cells the numbers do not exceed 8 and 5% for KO#1 and KO#2, respectively (Fig. 7B, C).

### Differentiation is not restored in *HAX1* KO cells upon free fatty acid supplementation

Riffelmacher et al. [3] reported that free fatty acids are sufficient to restore differentiation in autophagy-deficient neutrophil precursors, by providing an energy source that cannot be acquired by lipid droplet degradation. We used the same approach to check whether free FAs would be able to restore differentiation in *HAX1* KO mutants, which are also deficient in LD degradation. Indeed, for quiescent cells, supplemented with free fatty acids (FAs) the difference between WT and *HAX1* KO becomes less important (KO#1) or not important (KO#2), indicating that despite a block in the degradation of lipid droplets, these cells can still benefit from the supply of free FAs, to support β-oxidation. However, for cells induced with ATRA and supplemented with free FAs, the difference becomes huge, especially for *HAX1* KO#2 (almost 8 times more differentiated cells in WT, Fig. 7B, C), indicating that *HAX1* KO cells cannot support their differentiation using free FA supply.

### Morphological and functional assessments of neutrophil differentiation

#### Morphological evaluation

**Fig. 8:**
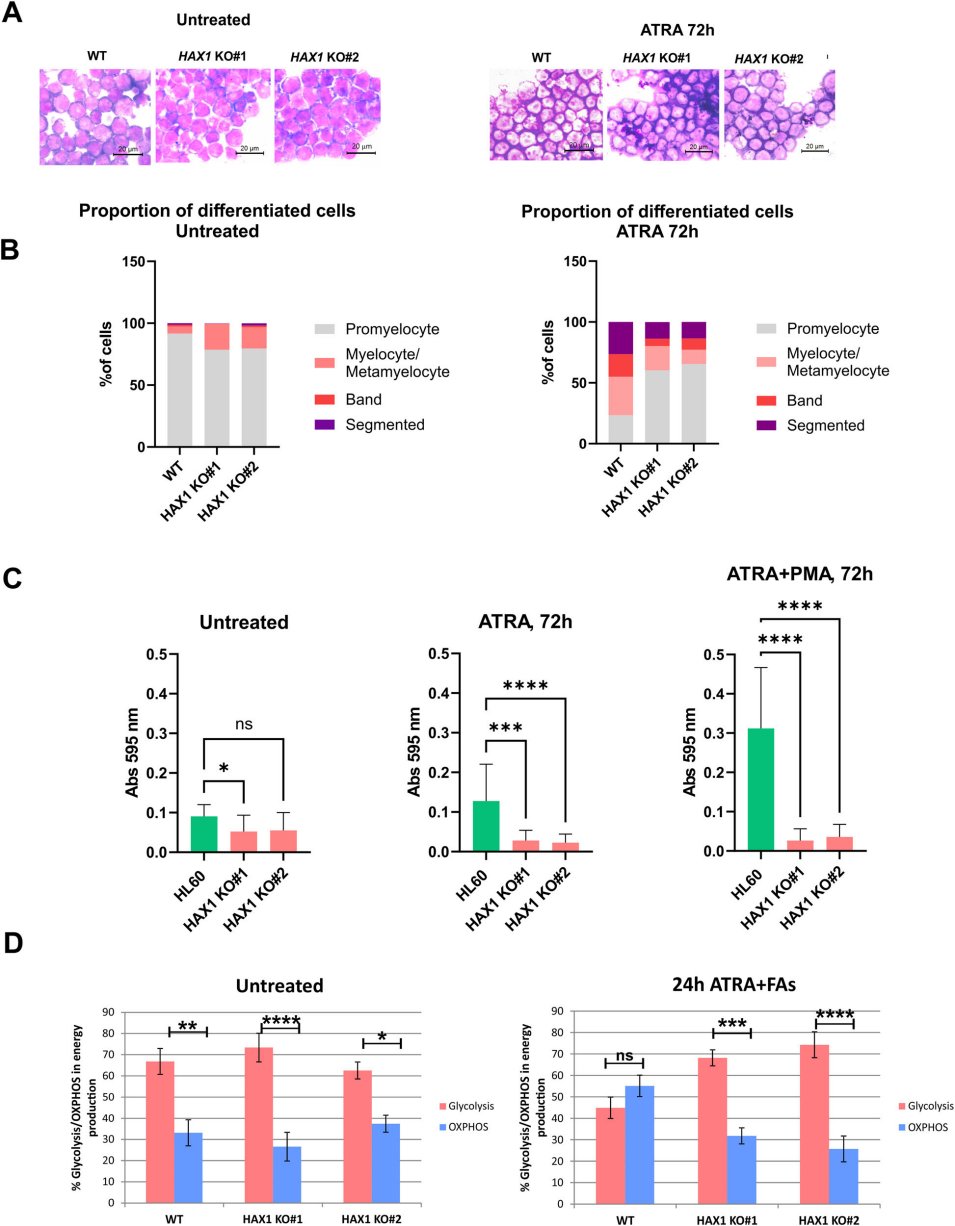
Morphological and functional assessment of *HAX1* WT/KO cells differentiation. **A**, **B** MGG staining. Differentiation assessed by morphological changes of the nucleus. MGG staining of the nuclei of the untreated and induced cells (58 μM ATRA, 72 h). **A** Representative images, **B** Quantification of different stages of differentiation for HL-60 WT and *HAX1* KO cells after ATRA treatment. WT/*HAX1* KO#1/*HAX1* KO#2, Untreated: *n* = 8/7/7, ATRA treatment: *n* = 10/10/10. **C** NBT Assay. WT/*HAX1* KO#1/*HAX1* KO#2, 3 biological replicates of each experiment, Untreated: *n* = 12/13/14, ATRA treatment: *n* = 14/13/14, ATRA + PMA treatment: *n* = 15/14/14. Statistical analysis: One-way ANOVA, Dunnett, statistical details Supplementary Table S1. **D** The assessment of mitochondrial respiration vs. glycolysis. A ratio of oxidative phosphorylation (OXPHOS) to glycolysis evaluated using Seahorse XF Cell Mito Stress Test Kit for *HAX1* WT/KO cells non-induced and induced by ATRA+FAs. Values calculated from basic ATP production rate. Statistical analysis: One-way ANOVA, Tukey, *n* = 8–9, biological replicates: 2–4, statistical details: Supplementary Table S1. Error bars: SEM.

To evaluate neutrophil differentiation independently of CD11b we performed May-Grunwald-Giemsa (MGG) staining of differentiated and non-differentiated cells, to observe the occurrence of gradual changes in the shape of the nuclei. Untreated and ATRA-treated (after 5 days of treatment) *HAX1* WT/*HAX1* KO cells were compared. The percentage of promyelocyte, myelocyte/metamyelocyte, band cells and segmented neutrophils was calculated in all samples, indicating intense differentiation in WT cells and very limited differentiation in *HAX1* KO cells (Fig. 8A, B, Supplementary Fig. S7).

#### NBT assay

To additionally strengthen and confirm the analysis we conducted NBT assay, which measures the superoxide-dismutase activity in the terminally differentiated granulocytes after their activation. The assay was performed for non-induced cells, ATRA-induced cells and ATRA-PMA induced cells (Fig. 8C). The results indicate significant increase in enzyme activity with and even without PMA induction, but observed only for WT cells, indicating their complete differentiation.

#### Differences in metabolic status of *HAX1* WT/*HAX1* KO cells upon ATRA+FAs treatment

Differentiating neutrophil precursors undergo metabolic shift towards mitochondrial respiration and thus, the metabolic shift represents independent functional verification of the differentiation process. Metabolic shift can be traced by the assessment of the respective share of glycolysis and oxidative phosphorylation (OXPHOS) in total energy production in the differentiating cells. To compare the level of glycolysis and mitochondrial respiration in *HAX1* WT and *HAX1* KO (#1 and #2) cells, the rate of ATP production from glycolysis and OXPHOS was simultaneously assessed using the Seahorse XF Cell Mito Stress Test. The analysis was performed in untreated, quiescent cells and ATRA+FAs induced cells. Treatment with ATRA alone was tested and evaluated as not sufficient, since it induces only about 30% of cells in the wild type and the assay is not sensitive enough to detect the difference (data not shown). The experiment was performed after 24 h of ATRA treatment because the metabolic shift spikes only at the first stages of differentiation, at the myeloblast-promyelocyte transition, further differentiation to myelocyte, metamyelocyte, band cell and segmented neutrophil is accompanied by reverting to glycolysis as the energy source. In non-induced cells, no differences in the respiration between *HAX1* WT and KO cells were observed (Fig. 8D, left panel), with significant dominance of glycolysis in all cases. However, after treatment with ATRA+FAs, under which conditions the difference between the cell lines in the ratio of differentiated vs. non-differentiated cells is huge (as evidenced in Fig. 7C), we have observed a shift towards oxidative phosphorylation for wild-type cells, which was not present in *HAX1* KO cells (Fig. 8D, right panel, Supplementary Fig. S4). Metabolic shift observed in control cells confirms that the differentiation is induced.

## Discussion

The involvement of HAX1 in granulopoiesis is known for years, and several possible explanations of this involvement have been presented [7–9], but it seems that this issue still requires clarification, probably because the molecular functions of HAX1 are hard to define; it is classified as intrinsically disordered protein (lacking 3D structure) and these proteins are known for multiple, tunable interactions, high adaptability and dynamics, but also their functionality is not as strictly defined as for the well-structured proteins [6]. Here we report our discovery of the previously unknown impact of HAX1 on lipid metabolism, and, in consequence, on neutrophil differentiation.

In the previous report [13], we have analyzed differences in expression profiles between *HAX1* WT and HAX1-deficient cells in the HL-60 promyelocytic cell line and we have found that there are two main groups of differentially expressed genes: (1) encoding proteins involved in ribosome biogenesis and translation and (2) encoding factors involved in energy generation in mitochondria, mainly respiratory electron transport chain and oxidative phosphorylation. The role in energy metabolism and RNA processing is also suggested by our HAX1 protein interactome studies [14].

Our starting point in this report was a quantitative proteomic analysis (iTRAQ) of the differences between *HAX1* WT and HAX1-deficient cells in the HL-60 promyelocytic cell line. It should be noted that in all subsequent experiments we have used two independently generated CRISPR/Cas9 *HAX1* knockouts (obtained with different RNA guides). As can be observed in Supplementary Fig. S2, *HAX1* KO#1 contains traces of the HAX1 protein and therefore it was not used in the initial iTRAQ analysis. The presence of the traces of HAX1 in *HAX1* KO#1 is reflected in practically all presented assays, including fatty acid tests and lipid metabolic tests, amino acid tests, differentiation assays and respiration analysis, with *HAX1* KO#1 always presenting a less stringent phenotype.

The initial standard analysis of the results (Gene Ontology analysis using the DAVID Annotation Tool and the Reactome Pathway Database) basically confirmed involvement in ribosome biogenesis, translation and energy metabolism, but also revealed differences in amino acid biosynthesis (mainly arginine and serine) and in lipid metabolism. Pathway analysis indicated changes in neutrophil degranulation, presumably through modulation of Rho GTPase activity, which is consistent with the fact that HAX1 interactions with different members of the Rho family of small GTP-ases have been previously reported [19–21]. Neutrophil degranulation may also be related to irregularities in calcium influx [22], which, as discussed further, could also be HAX1-dependent. The protein most up-regulated in *HAX1* KO cells is argininosuccinate synthetase encoded by the *ASS1* gene, an enzyme involved in arginine biosynthesis and the urea cycle. ASS1 catalyzes the penultimate step of arginine biosynthesis: the formation of arginosuccinate from aspartate and citrulline. This is in line with the general results of proteome analysis, which indicated differences in arginine and serine biosynthesis. ASS1 deficiency results in urea cycle disorder: citrullinaemia. The performance of ASS1 depends on the availability of aspartate, which is one of its substrates. Cytoplasmic aspartate is provided by the malate-aspartate shuttle and one of its transporters from mitochondria, citrin (SLC25A13). Previously [14], we have reported the interaction of HAX1 with citrin and also with Aralar (SLC25A12), the second component of the shuttle. Here, we confirmed and mapped the interaction to the N-terminal domain of citrin, which contains the calcium-binding EF-hand domain. As observed by Thangaratnarajah et al. [23], calcium-induced conformational changes in the carrier protein result in the opening (calcium-bound) or closing (not bound) of the transporting vestibule, which regulates carrier activity. Thus, HAX1 binding to this regulatory N-terminal part may interfere with proper regulation of carrier capacity. This would result in inhibition of the malate-aspartate shuttle, with specific metabolic consequences, including a decrease in serine and aspartate levels and an increase in lactate. Indeed, such differences have been observed for HAX1-rich and HAX1-deficient cells, indicating that HAX1 affects the malate-aspartate shuttle. HAX1 interaction with SLC25A13 (and related carriers) may perturb Ca^2+^-dependent regulation, contributing to MAS dysfunction; the direction and magnitude of flux changes require further investigation. We report here for the first time the involvement of HAX1 in the regulation of the shuttle. Future experiments should focus on direct quantification of these changes (lysosomal pH, MAS flux) and further analysis of the interaction with carriers and the role of calcium ions.

Comparison of the results of RNA-seq/iTRAQ indicate roughly the same categories of genes/proteins differentially expressed in *HAX1* KO cells. However, we have performed an additional, deeper analysis of the iTRAQ proteomic data, designed to recognize metabolic differences by tracking changes in the abundance of metabolic enzymes. The analysis of proteomic data using the QSM platform shifted the focus to lipid metabolism and energy metabolism. The analysis revealed that the most significant differences concern lipid metabolism (fatty acid utilization and triglyceride [TAG] storage), with the second most important group related to energy metabolism. To verify the predictions of this model, several physiological tests were performed, including the content and uptake of fatty acids and ketone bodies. Although metabolic tests verified significant differences in fatty acid metabolism between *HAX1* WT and KO cells, some tests (FAs uptake, urea production), while still showing significant differences between WT and KO cells, contradicted the predictions of the model. The model is also not consistent with our interpretation of β-oxidation levels. However, this is understandable, if we assume that the model correctly predicts higher fatty acid storage in *HAX1* KO cells, but cannot predict inhibition of the degradation of stored lipids. This fact, together with an incorrect prediction of the increase in fatty acid uptake, leads to the faulty conclusion that β-oxidation is increased in cells deficient in HAX1, due to the higher lipid content. The discrepancy in urea production can probably be explained by MAS upregulation, resulting in higher urea levels in cells deficient in HAX1, for which the model does not predict.

Interestingly, mass spectrometry for a specific fatty acid panel revealed a higher content of most FAs tested in *HAX1* KO cells compared to the wild type, but the free fatty acid concentration assay showed significantly lower FFAs in *HAX1* KOs. The explanation of this discrepancy may be that these results are method-dependent: while mass spectrometry detects both, free FAs and FAs stored in a form of TAG (esterified), the assay (due to enzymatic constraints) detects only non-esterified, free FAs. This interpretation suggests that fatty acids are stored in HAX1-deficient cells as triglycerides in the form of lipid droplets (LDs). We confirmed this assumption by analyzing the number of LDs accumulated in *HAX1* WT vs. *HAX1* KO cells. Interestingly, a significantly higher number of LDs in *HAX1* KO cells becomes non-significant after treatment with the autophagy inhibitors bafilomycin A1 and E64D. Both of these inhibitors act in the late stage of autophagy and neither of them prevents the formation of autophagosomes. Our explanation is that lipid droplet autophagy is blocked in HAX1-deficient cells at the stage of fusion of the autophagosome with the lysosome. Therefore, autophagy inhibitors, which act at the same stage, inhibit the degradation of LDs in the wild type, increasing the number of LDs per cell, but this effect cannot be observed in *HAX1* KO cells, since the degradation of LDs in these cells is already blocked. This hypothesis was also confirmed by the autophagosome acidification experiment, in which we traced the fusion of the autophagosome with the lysosome, which demonstrated that this fusion can be observed in *HAX1* WT, but to much less extent in *HAX1* KO cells. While the molecular explanation for this inhibition is not completely clear at the moment, it should be noted that bafilomycinA1 has been demonstrated to prevent autophagosome-lysosome fusion and this effect was phenocopied by genetic depletion of the Ca2+ pump SERCA [24], a protein known to be regulated by HAX1 [25]. The SERCA2 inhibitor thapsigargin has also been shown to prevent autophagosome-lysosome fusion [26]. Accordingly, it has been proposed [24] that an abnormal calcium gradient prevents lysosomal acidification and fusion. The effect of HAX1 on SERCA2 and its involvement in the regulation of calcium homeostasis is known for a long time [25, 27–29] and thus, the effect of HAX1 on intracellular calcium concentration may contribute to the explanation why LD autophagy cannot be completed in *HAX1* KO cells.

Interestingly, autophagosome numbers are also elevated in HAX1-deficient cells, and after inhibition of autophagy with bafilomycin or E64D their number remains elevated. Our explanation is that autophagy of LDs represents only a fraction of the total autophagy, which increases in cells deficient in HAX1, possibly not only because of accumulation of LDs, but also some other factors, for example, protein aggregation. Our team has already reported an increase in protein aggregation in HeLa cells deficient in HAX1 [14]. Thus, increased total autophagy causes an increase in the number of autophagosomes, and, when their degradation is blocked after inhibitor treatment, the numbers simply reflect the difference in total autophagy burden. The conclusion is that, although LD autophagy appears to be completely blocked in HAX1-deficient cells, other types of autophagy are still ongoing at some level.

Inhibition of lipid droplet autophagy should have a profound effect on neutrophil differentiation. It has been demonstrated that early during neutrophil differentiation from hematopoietic stem cells a metabolic shift is observed, from mainly glycolytic precursors towards FAO-OXPHOS in differentiating cells and back to mainly glycolytic mature neutrophils [30]. This metabolic shift depends on autophagy. As described by Riffelmacher et al. [3], autophagy-dependent generation of free fatty acids from lipid droplets is critical for neutrophil differentiation, because it fuels β-oxidation, supplying ATP for this process, which is very energy-demanding. Therefore, inhibition of LD degradation, and, subsequently, lower energy supply in HAX1-deficient cells should explain differentiation arrest observed in HAX1-deficient neutrophil precursors. To test this hypothesis, we have established a new model of HAX1-depended differentiation arrest using HL-60 cell line and *HAX1* knockouts derived from this cell line. *HAX1* mutations are known to cause severe congenital neutropenia (Kostmann disease) due to inhibition of neutrophil granulocyte differentiation [7]. This effect was replicated by Fan et al. [9] using an induced pluripotent stem cell (iPSC) in vitro differentiation system. The authors observed that HAX1-deficient iPSCs had a significantly reduced capacity to differentiate into neutrophil granulocytes. Here we report our model of HAX1-dependent differentiation arrest in a simpler HL-60-based system. The HL-60 cell line is an established model of neutrophil differentiation, usually with retinoic acid or DMSO as an inducer [31–34]. We have observed that there is a significant difference in retinoic acid-induced differentiation between HL-60 WT and the two HL-60 *HAX1* KO cell lines. Subsequently, we supplemented differentiating cells with free fatty acids to test whether this supplementation would restore the capacity of HAX1-deficient cells to differentiate. Interestingly, although this approach boosted the process in all cell lines, it did not eliminate a significant difference in differentiation, despite the fact that *HAX1* KO cells were directly supplied with a potential energy source that they lacked due to inhibited lipid droplet autophagy. However, this can be explained by the fact that, according to our results, fatty acid uptake is also impaired in HAX1-deficient cells. Therefore, these cells are dually affected: in fatty acid uptake and in lipid droplet degradation, resulting in low free fatty acid content in the cytoplasm and insufficient energy source to fuel β-oxidation.

Consistent with this line of reasoning, early in neutrophil differentiation (24 h after induction) we have observed increased mitochondrial respiration (an increased ratio of oxidative phosphorylation to glycolysis) in wild type cells, but not HAX1-deficient cells. This may signify that a shift toward β-oxidation, which usually accompany differentiation, is blocked in HAX1-deficient cells. This assay was especially difficult because in cells induced by ATRA+FAs, we have observed increased apoptosis, which disrupts the ratio of live/dead cells in the assay.

In general, in this report we present new results that indicate for the first time the role of HAX1 in the regulation of lipid metabolism and the mitochondrial malate-aspartate shuttle. We have observed two different effects of *HAX1* knockout on lipid metabolism: reduced FA uptake and blocked lipophagy. These combined effects limit the rescue by exogenous FAs under ATRA-induced differentiation creating the constraints at both uptake and mobilization, explaining insufficient differentiation in *HAX1* KOs. These results allow to take a new angle in describing the role of HAX1 in the regulation of neutrophil differentiation and, generally, in energy metabolism. Additionally, we have established a new, simple model of HAX1-mediated differentiation arrest, which mimics certain phenotypic aspects of Kostmann disease and is based on the HL-60 promyelocytic cell line.

## Materials and methods

### Cell lines and culture conditions

HL-60 (ACC 3, DSMZ, Germany), acute myeloid leukemia cell line, and the two independent *HAX1* CRISPR knockout cell lines*: HAX1* KO#1 and *HAX1* KO#2, Supplementary Fig. S2 (knockout procedure described in ref. [13]), HeLa (CCL-2, ATCC, USA), cervical adenocarcinoma. Cells were cultured in RPMI supplemented with L-glutamine (Biowest) and 10% FBS (ThermoFisher Scientific) at 37 °C in a 5% CO_2_. Transfection: TurboFect or Lipofectamine2000 (ThermoFisher Scientific), according to the manufacturer’s instructions.

### Vectors and constructs

FUW mCherry-GFP-LC3 was a gift from Anne Brunet (Addgene plasmid # 110060; http://n2t.net/addgene:110060; RRID:Addgene_110060).

Constructs: pcDNAFLAG: backbone: pcDNA3.1 (ThermoFisher Scientific), Flag tag generated by the annealing of complementary primers (5’- CGAGGGCGGCGGCTCTAGAATGGACTACAAGGACGACGACGACAAGGCTAGCGGCGGCGGCAA-3’, 5’- AGCTTTGCCGCCGCCGCTAGCCTTGTCGTCGTCGTCCTTGTAGTCCATTCTAGAGCCGCCGCCC-3’) and cloned on XbaI/NheI, HindIII.

pcDNAFLAG C SLC25A13, backbone: pcDNAFLAG, PCR product encoding SLC25A13 (primers: 5’- GAATCCAGGATCCATCAACCGGGGCGAATCATGGC-3’, 5’- GCAGTACTCTAGACGGAATTCTGTATGGGCCTCCACCAATAGC-3’) cloned using BamHI, XbaI. pcDNAFLAG C SLC25A13 (ΔN), backbone: pcDNAFLAG C SLC25A13, PCR product encoding SLC25A13 ΔN (primers: 5’- GGCGAGCAGGATCCGTATGGTTGCAGAGTCGGCCTACAG-3’, 5’- TAGAAGGCACAGTCGAGG-3’), cloned using BamHI, XbaI. pcDNAFLAG C SLC25A13 (ΔC), backbone: pcDNAFLAG C SLC25A13, PCR product encoding SLC25A13 ΔC (primers: 5’- CGCAAATGGGCGGTAGGCGTGTACG-3’, 5’- GACGCCTCTAGACATGTAGAACCATCGCTGTAG-3’), cloned using BamHI, XbaI. pEGFPc1SLC25A13, backbone: pEGFPc1, SLC25A13 cloned from pcDNAFLAG C SLC25A13 using HindIII (Klenow fill-in), BamHI. pSpotTagn1HAX1, backbone: pEGFPn1HAX1 (described in 10.1111/febs.12066), SpotTag generated by the annealing of complementary primers (5’-TCGAGGGACCGGTCGGCGGCGGACCAGACCGCGTGCGCGCCGTGAGCCATTGGAGCAGCTAAG-3’, 5’- CTAGCTTAGCTGCTCCAATGGCTCACGGCGCGCACGCGGTCTGGTCCGCCGCCGACCGGTCCC-3’) leaving the overhang for ligation with AgeI, XbaI. pMitoEGFPc1, backbone: pEGFPc1 (Clontech), MTS COX41 sequence cloned from pMitoTimer (a gift from Zhen Yan, Addgene plasmid # 52659; http://n2t.net/addgene:52659; RRID:Addgene_52659), AgeI, NheI. pMitoEGFPc1+SpotTagn1, backbone: pSpotTagn1HAX1, MitoEGFP cloned from pMitoEGFPc1 using XhoI/SalI, NheI.

### Mass spectrometry analysis

Four separate cultures of HL-60 and HL-60 *HAX1* KO #2 were prepared for each cell line. 8 × 10^6^ cells were collected from each sample. Mass spectrometry analysis was performed at the Mass Spectrometry Laboratory of the Institute of Biochemistry and Biophysics PAS (Warsaw).

#### Protein digestion

The samples were prepared according to Filter Aided Sample Preparation protocol with minor modifications [35]. Briefly, the protein sample was incubated for an hour with 10 mM tris(2- carboxyethyl)phosphine (TCEP) at 60 °C, transferred to a Vivacon 30 kDa molecular weight cut-off filter (Sartorius Stedim Biotech, Goettingen, Germany), spun at 14,500 × *g* for 15 min and washed with urea solution (8 M urea in 100 mM Triethylammonium bicarbonate buffer [TEAB]) before cysteine blocking by 5 min incubation at room temperature with 30 mM s-methylmethanethiosulfonate (MMTS). Proteins were washed three times with urea buffer and three times with 100 mM TAEB. After each addition, the samples were centrifuged for 15 min at 14,500 × *g*. Digestion was carried out overnight using 3 µg of trypsin (Promega GmbH, Mannheim, Germany) at 37 °C. Peptides were eluted from spin filters by two washes with 100 mM TAEB and one wash with 500 mM NaCl solution. Next, samples were vacuum-dried, reconstituted in 40 µl of 200 mM TEAB and peptide concentrations were measured using Pierce Quantitative Colorimetric Peptide Assay (Thermo Fisher Scientific, Waltham, MA, USA). Each sample was labeled with iTRAQ 8plex (SCIEX, Framingham, MA, USA) tags in isopropanol for 2 h on vortex. The reaction was quenched by the addition of 8 µL 5% hydroxylamine. The combined iTRAQ sample was desalted using three 30 mg Oasis HLB cartridges (Waters, Milford, MA, USA). Peptides were eluted from columns with 400 µl 75% acetonitrile (ACN) and 0.1% FA. Aliquots were dried and resuspended in 500 µl 10 mM ammonium hydroxide.

#### Reversed-phase peptide fractionation at high pH

iTRAQ labeled peptides were fractionated using high-pH reverse-phase chromatography on XBridge Peptide BEH C18 column at a flow rate of 0.8 ml/min for 27 min on Waters Acquity UPLC H-class system. The peptide elution profile was monitored at 214 nm by UV detector. 25 fractions were dried in Speedvac and reconstituted in 0.1% formic acid in water (Evosep solvent A) by 15 min sonication and 15 min vortexing.

#### Mass spectrometry

Fractions were analyzed using LC-MS system composed of Evosep One (Evosep Biosystems, Odense, Denmark) directly coupled to a Orbitrap Exploris 480 mass spectrometer (Thermo Fisher Scientific, Bremen, Germany) using Evotips C18 trap columns. Bound peptides were washed and covered with solvent A. Chromatography was carried out at a flow rate of 500 nl/min using the 44 min (30 samples per day) preformed gradient on EV1106 analytical column (Dr Maisch C18 AQ, 1.9 µm beads, 150 µm ID, 15 cm long, Evosep Biosystems, Odense, Denmark.

#### MS data analysis

Offline recalibration, as well as peptide and protein identification, was performed in the MaxQuant/Andromeda software suite (version 1.6.17.0) [36] using *Homo sapiens* a full Uniprot database (version 20210426). The search included tryptic-generated peptides, Metylthio (C) was set as a fixed modification and Oxidation (M) as a variable one. Reporter MS2 quantification was specified in order to obtain values for quantitative analysis. The reverse database was used for the validation of the target/decoy statistical results, peptide and protein FDR was set to 0.01. Protein groups along with quantitative data were further analyzed in Perseus (version 1.6.14.0) [37]. Proteins with less than 3 samples in each groups were filtered out. iTRAQ reporter values were normalized on average sum of intensity [38]. Reporter intensities were log2 transformed, missing values were replaced with data from normal distribution (width 0.3, down shift 1.8) separately for each column and PCA analysis and Volcano Plot, were performed. Subsequently, two-sample *t*-tests with Benjamini–Hochberg FDR were performed to compare expression changes between groups. The significance threshold for the resulting *q*-value was 0.05.

#### Pathway analysis

Standard analysis: DAVID Functional Annotation Tool [39, 40], Enrichr [41, 42].

QSM (Quantitative System Metabolism) Data Analysis (Doppelganger Biosystem GmbH, Germany):

QSM (Quantitative System Metabolism) Data Analysis (Doppelganger Biosystem GmbH, Germany): Metabolic functions were evaluated using a model of central carbon metabolism [43] coupled to a detailed lipid-droplet metabolism model [44]. The integrated model captures key metabolic pathways and mitochondrial processes and incorporates enzymatic regulation by substrates, allosteric effectors, and hormonal signaling [43–46]. For each sample, an individualized model parameterization based on quantitative proteomics (iTRAQ) was instantiated. Maximal activities of enzymes and transporters were scaled according to:

$${v}_{\max }^{{E}_{{sample}}}={v}_{\max }^{{E}_{{ctrl}}}\frac{{E}_{{sample}}}{{E}_{{ctrl}}}$$

where ${v}_{\max }^{{E}_{{ctrl}}}$ denotes the mean maximal activity of enzyme E in control (wild-type) tissue, *E*_*sample*_ denotes the enzyme abundance of E in the individual sample, *E*_*ref*_ denotes the mean abundance of E in the control (wild-type) samples. Simulations covered physiological conditions from fasted to fed as described in ref. [43], and metabolic functions were assessed after the system reached steady state. The analysis takes into account the regulation of metabolic enzymes and transporters by substrate affinities, allosteric regulations, and the short-term regulation by insulin and catecholamines. Hormone-dependent regulation of central energy metabolism by reversible enzyme phosphorylation was taken into account as described in ref. [46]. Individual model instantiations were generated based on proteomic profiles as described by Berndt et al. [43]. Metabolic capacities for the utilization of glucose, fatty acids, lactate, ketone bodies and branched chain amino acids were defined by the magnitude of flux changes in response to changes in the concentration of the respective plasma metabolite, while the non-varied plasma metabolites were kept constant. The external conditions for all simulations are given in ref. [45]. ATP production capacity was assessed in a physiological overnight fasted state, where plasma metabolite concentrations are not independent of each other, as glucose stimulates the release of insulin from beta cells and concomitantly reduces the release of glucagon from alpha cells in the pancreas and both hormones control the release of fatty acids from adipose tissue. The interdependence between plasma glucose, plasma hormone and plasma fatty acid concentration was taken into account by using a sigmoid Hill-type function describing the experimentally determined glucose-insulin and glucose-fatty acid relations [43, 44, 46]. Plasma profiles used are given in the Supplementary Table S4.

#### Fatty acid MS panel

Quantification of 18 fatty acids (Caprylic acid, Decanoic acid, Lauric acid, Myristic acid, Linoleic acid, α-Linolenic acid, γ-Linolenic acid, Trans-vaccenic acid, Arachidic acid, Eicosapentaenoic acid, Arachidonic acid, Dihomo-γ-linolenic acid, Adrenic acid, Erucic acid, Docosahexaenoic acid, Nervonic acid, Oleic acid and Palmitoleic acid; certified standards purchased from Sigma Aldrich) was achieved using Acquity UPLC system (Waters) coupled with Xevo TQ mass spectrometer (Waters). Chromatographic separation was achieved using BEH C18 (2.1 × 100 mm 1.7 µm) column (Waters). Column oven was kept at temperature of 50 °C. Mobile phase A consisted of 0.1% NH4OH in MQ water, mobile phase B consisted of pure LC-MS grade acetonitrile. Method started at 20% phase B and linearly increased to 95% B over next 2.8 min. The total run time of the method was 4.0 min.

Samples were prepared for protein precipitation by adding 150 µL of internal standard containing isotopically labeled analogues in HPLC grade acetonitrile (Decanoic acid – 1,2 C13, Myristic acid – 1,2 C13, Stearic acid - 17,17,18,18,18 - d5, Eicosapentaenoic acid - 19,19,20,20,20 - d5, Arachidonic acid - 5,6,8,9,11,12,14,15 - d8, Docosahexaenoic acid – 21,21,22,22,22 – d5 at concentration 5 µg/mL). Samples were vortexed for 1 min at 1 500 RPM and treated with 20 µL of 6 N HCl, followed by boiling at 104 °C for 45 min, resulting in cell lysis. Further, samples were submitted to saponification by adding of 20 µL of 10 N NaOH. Samples were boiled for the second time at 104 °C for 45 min., reacidified with 100 µL of 6 N HCl and vortexed for 1 min at 1500 RPM. Liquid-liquid extraction was performed by adding 700 µL of HPLC grade hexane, vortexing samples for 10 min at 1500 RPM and centrifuging for the next 10 min at 14,000 RPM. Next, 700 µL of non-polar upper phase was transferred to new polypropylene sample tube and evaporated to dryness under nitrogen flow at 50 °C. Lipid residue was reconstituted in 100 µL of 5% NH4OH in 65% HPLC grade methanol.

### Metabolic tests

Lactate Assay Kit, MAK064, Colorimetric& Fluorescence Assay (Sigma-Aldrich), Urea Colorimetric Assay Kit II, MAK410 (Sigma-Aldrich), Beta-Hydroxybutyrate Assay Kit, Colorimetric Assay, MAK041 (Sigma-Aldrich), Free Fatty Acid Quantitation Kit, Colorimetric& Fluorescence Assay, MAK044 (Sigma-Aldrich), Ketone Body Assay Kit, Colorimetric Assay, MAK134 (Sigma-Aldrich), Fatty Acid Uptake Kit, Fluorescence Assay, MAK156 (Sigma-Aldrich), insulin treatment: 30 min, 150 nM insulin (Sigma-Aldrich), Acetoacetate Colorimetric Assay Kit, MAK199 (Sigma-Aldrich). Test were performed according to the manufacturer’s instructions. Measurement reading: Victor 3 plate reader (Perkin-Elmer).

### Co-immunoprecipitation

HeLa cells were transfected with constructs encoding SLC25A13 or truncated SLC25A13 (constructs ΔN and ΔC) tagged with FLAG. pcDNA FLAG N plasmid was used as a negative control. Cells were harvested 24 h after transfection and lysed in NP-40 lysis buffer (10 mM Tris/Cl pH 7.5, 150 mM NaCl, 0.5 mM EDTA, 0.5% Nonidet P40) supplemented with Complete Protease Inhibitor Cocktail (Roche, 05056489001). Protein A-coated agarose beads (Dynabeads, Invitrogen) were preincubated with anti-FLAG antibody (MA1-91878, ThermoFisher Scientific) for 4 h at 4 °C and used for immunoprecipitation with cell lysate. Immunoprecipitated protein extracts were analyzed by Western blot using anti-HAX1 antibody (PA5-27592, ThermoFisher Scientific). Uncropped image in Supplementary Material.

### Immunofluorescence

All analyzes were done using a Zeiss LSM800 Axio Observer Z1 confocal microscope.

#### FRET assay

HeLa cells were co-transfected with constructs EGFP-SLC25A13 and pSpotTagn1HAX1. Spot tag was recognized by ATTO594 alpaca one-step antibody (anti-Spot-Tag VHH/ Nanobody conjugated to ATTO594, eba594, Chromotek). FRET was performed in the region of co-localization in mitochondria, using the acceptor photobleaching method (donor: EGFP, acceptor: ATTO594). Laser 561 nm was used for photobleaching. EGFP signal detection: excitation 488 nm, emission: 450–580 nm. Positive control: pMitoEGFPc1_SpotTagn1 (MTS [mitochondrial targeting signal] from COX41 [Cytochrome C Oxidase Subunit 4 Isoform 1] with ATTO594. Negative control: pSpotTagn1HAX1 and pMitoEGFPc1. Endogenous HAX1 IF :anti-HAX1 Proteintech 11266-1-AP.

#### Lipid droplet/autophagosome quantification

10^6^ HL-60 cells were placed on slides covered with poly-L-lysine (Sigma). In case of treated cells, autophagy inhibitors were used: E64d (POL-AURA PA-03-9790-L) at 10 µg/ml for 20 min., or bafilomycin A (Cell Signaling 54645S) at 100 nM, incubation time: 2 h. In the next step cells were stained for 20 min. with Droplite Red Staining Solution (Cell Navigator Fluorimetric Lipid Droplet Assay Kit Red Fluorescent AAT Bioquest 22725 kit protocol). After short spin (1000 rpm for 2 min.) and rinse with PBS cells were fixed with 4% formaldehyde (Chempur 114321734) for 15 min on ice, permeabilized in 0.5% Triton X-100 (Sigma) for 40 min. and incubated overnight at 4 °C with anti-LC3A/B (D3U4C) primary antibody (Cell Signaling #12741) followed by the incubation with secondary antibody (AlexaFluor 488 Cell Signaling 4412S) for 2 h and DAPI (Sigma D9542) for 10 min. Cells were observed using the Zeiss LSM800 Axio Observer Z1 microscope with a 63×/1.40 Oil DIC M27 objective. Images were analyzed using ImageJ software (converted to 8-bit, thresholded, transformed into a binary map, and analyzed using option ‘analyze particles’).

#### Autophagosome acidification assay

HL-60 cell lines (*HAX1* WT, *HAX1* KO#1 and #2) were transfected with the GFP-mCherry-LC3 vector (Addgene: Plasmid #110060) that allows visualizing free autophagosomes (GFP and mCherry fluorescence) and autophagosomes fused with the lysosome (only mCherry fluorescence, due to GFP destruction at low pH). After 16 h from transfection half of the samples were treated with glucose-free medium (RPMI Medium 1640 Ref: 11879-020, Gibco) for 2 h and fixed. The imaging was performed for untreated and treated cells using Zeiss LSM800 Axio observer Z1 microscope with a 63×/1.40 Oil DIC M27 objective (emission wave length of 488 nm and 594 nm, corresponding to the GFP and mCherry proteins, respectively). Fluorescence intensity was measured for each channel (ImageJ). A single measurement is represented by the ratio of the mCherry to the GFP fluorescence intensity value and corresponds to one (up to three) randomly selected autophagosome in one cell.

### Neutrophil differentiation assay

HL-60 cells WT and *HAX1* KO were cultured in RPMI 1640 medium (L0498-500, Biowest) supplemented with 10% FBS (S181H-500, Biowest) and antibiotic-antimycotic solution (Sigma-Aldrich) in 5% CO_2_, at 37 °C. Cells were passaged every 2 days and kept at confluence lower than 0.5mln/10 mL. To differentiate into neutrophils, fully viable (>90% viability) culture of HL-60 cells was incubated for 3 days with 58 μM ATRA (Sigma-Aldrich) or 58 μM ATRA supplemented with a mix of free fatty acids (Free Fatty Acid Lipid Mixture 1, Chemically Defined, a mix of 10 FAs, 10 μg/mL each, linoleic, linolenic, myristic, oleic, palmitic and stearic acids, 2 μg/ml arachidonic acid+ 0.22 mg/ml cholesterol, L0288, Sigma Aldrich, 10 μl/mL of the medium). After 3 days of differentiation, the viability of the cells was determined using trypan blue (Gibco) dye.

### Assessment of differentiation

#### Flow cytometry

Cell differentiation was estimated by verifying the expression of CD11b (ICRF44 PERCPEF710, 46-0118-42, ThermoFisher Scientific), CD33 (CoraLite® Plus 488 Anti-Human CD33, WM53, CL488-65272 Proteintech) using BD FACSCalibur flow cytometer (BD Biosciences). Data were analyzed with CellQuest software. Gating strategy and the details of data analysis are described in supplementary materials (Supplementary Fig. S5).

#### MGG staining

The procedure was performed as described [47]. Briefly, 2 × 10^5^ cells were deposited on a slide using CytoSpin, fixed with methanol and stained with May-Grunwald stain (2 min) and Giemsa (4 min). Slides were imaged using Leica DM 2000 Led microscope.

#### NBT assay

Colorimetric 4-nitroblue tetrazolium chloride (NBT) reduction activity assay (Sigma-Aldrich) was used to test the ability of the differentiated HL-60 cells to produce reactive oxygen species and reduce the colorless NBT to a deep blue, insoluble formazan crystals. A 96-well plate was coated with poly-L-lysine for 1 h, then washed with PBS and dried. 2 × 10⁴ cells were added per well, followed by the addition of 0.5 µg/ml PMA (Phorbol Myristate Acetate, Sigma-Aldrich) and 1 mg/ml NBT. The plate was incubated for 30 min at 37 °C in 5% CO_2_. After incubation, the wells were washed with warm 1 × PBS and methanol, and then left to air-dry. The formazan crystals were dissolved using 2 M KOH and DMSO. Absorbance was measured at 595 nm using microplate reader (Victor, Perkin-Elmer).

#### ATP production rate

The glyco/mito ATP Production Rate was assayed using an Agilent Seahorse XF HS Mini Analyzer, with XF Cell Mito Stress Test Kit (Agilent). Cells were seeded at a density of 6 × 10^4^ cells/well in 6-well XF microplates, cultured with DMEM containing 10% FBS (untreated, non-induced) and with the addition of 58 μM ATRA supplemented with a mix of free fatty acids (induced) for 24 h. One hour before starting measurements, the medium was replaced with XF base medium supplemented with 25 mM glucose and 2 mM pyruvate. After 1 h incubation in a CO_2_-free incubator at 37 °C to allow temperature and pH equilibration, baseline oxygen consumption rate (OCR) was measured, then followed by sequential injections with oligomycin (1.5 μM, Sigma-Aldrich) to measure the ATP linked OCR, oxidative phosphorylation uncoupler FCCP (1 μM, Sigma-Aldrich) to determine maximal respiration, and rotenone/antimycin A (0.5 μM, Sigma-Aldrich) to determine the non-mitochondrial respiration. Experimental treatments were performed in 2–5 wells of each plate as technical replicates. Normalization was performed with Hoechst (20 mM, Sigma-Aldrich) reading in each well.

### Statistical methods

Distribution was assessed using Shapiro–Wilk test. Results were analyzed using One-way ANOVA and post-hoc Tukey statistical tests, and the Wilcoxon rank-sum test (two-tailed). Statistics calculated using GraphPad Prism 9, Microsoft Excel and R.

## Supplementary information

Figures S1-S7

Tables S1-S5

Uncropped WB

Supplementary files legend

## Data Availability

Mass spectrometry proteomic data are available via ProteomeXchange with identifier PXD064511.

## References

[1] Hong CW. Current understanding in neutrophil differentiation and heterogeneity. Immune Netw. 2017;17:298–306.29093651 10.4110/in.2017.17.5.298PMC5662779

[2] Theilgaard-Monch K, Porse BT, Borregaard N. Systems biology of neutrophil differentiation and immune response. Curr Opin Immunol. 2006;18:54–60.16343884 10.1016/j.coi.2005.11.010

[3] Riffelmacher T, Clarke A, Richter FC, Stranks A, Pandey S, Danielli S, et al. Autophagy-dependent generation of free fatty acids is critical for normal neutrophil differentiation. Immunity. 2017;47:466–80. e465.28916263 10.1016/j.immuni.2017.08.005PMC5610174

[4] Suzuki Y, Demoliere C, Kitamura D, Takeshita H, Deuschle U, Watanabe T. HAX-1, a novel intracellular protein, localized on mitochondria, directly associates with HS1, a substrate of Src family tyrosine kinases. J Immunol. 1997;158:2736–44. 9058808

[5] Fadeel B, Grzybowska E. HAX-1: a multifunctional protein with emerging roles in human disease. Biochim Biophys Acta. 2009;1790:1139–48.19524642 10.1016/j.bbagen.2009.06.004

[6] Trebinska-Stryjewska A, Wakula M, Chmielarczyk M, Grzybowska EA. HAX1: a versatile, intrinsically disordered regulatory protein. Biochim Biophys Acta Mol Cell Res. 2023;1870:119538.37454914 10.1016/j.bbamcr.2023.119538

[7] Klein C, Grudzien M, Appaswamy G, Germeshausen M, Sandrock I, Schaffer AA, et al. HAX1 deficiency causes autosomal recessive severe congenital neutropenia (Kostmann disease). Nat Genet. 2007;39:86–92.17187068 10.1038/ng1940

[8] Skokowa J, Klimiankou M, Klimenkova O, Lan D, Gupta K, Hussein K, et al. Interactions among HCLS1, HAX1 and LEF-1 proteins are essential for G-CSF-triggered granulopoiesis. Nat Med. 2012;18:1550–9.23001182 10.1038/nm.2958PMC3941918

[9] Fan Y, Murgia M, Linder MI, Mizoguchi Y, Wang C, Lyszkiewicz M, et al. HAX1-dependent control of mitochondrial proteostasis governs neutrophil granulocyte differentiation. J Clin Invest. 2022;132:e153153.35499078 10.1172/JCI153153PMC9057593

[10] Anso E, Weinberg SE, Diebold LP, Thompson BJ, Malinge S, Schumacker PT, et al. The mitochondrial respiratory chain is essential for haematopoietic stem cell function. Nat Cell Biol. 2017;19:614–25.28504706 10.1038/ncb3529PMC5474760

[11] Tormos KV, Anso E, Hamanaka RB, Eisenbart J, Joseph J, Kalyanaraman B, et al. Mitochondrial complex III ROS regulate adipocyte differentiation. Cell Metab. 2011;14:537–44.21982713 10.1016/j.cmet.2011.08.007PMC3190168

[12] Zhang J, Khvorostov I, Hong JS, Oktay Y, Vergnes L, Nuebel E, et al. UCP2 regulates energy metabolism and differentiation potential of human pluripotent stem cells. EMBO J. 2011;30:4860–73.22085932 10.1038/emboj.2011.401PMC3243621

[13] Balcerak A, Macech-Klicka E, Wakula M, Tomecki R, Goryca K, Rydzanicz M, et al. The RNA-binding landscape of HAX1 protein indicates its involvement in translation and ribosome assembly. Cells. 2022;11:2943.36230905 10.3390/cells11192943PMC9564044

[14] Wakula M, Balcerak A, Rubel T, Chmielarczyk M, Konopinski R, Lyczek F, et al. The interactome of multifunctional HAX1 protein suggests its role in the regulation of energy metabolism, de-aggregation, cytoskeleton organization and RNA-processing. Biosci Rep. 2020;40:BSR20203094.33146709 10.1042/BSR20203094PMC7670567

[15] Broeks MH, van Karnebeek CDM, Wanders RJA, Jans JJM, Verhoeven-Duif NM. Inborn disorders of the malate aspartate shuttle. J Inherit Metab Dis. 2021;44:792–808.33990986 10.1002/jimd.12402PMC8362162

[16] Leeman DS, Hebestreit K, Ruetz T, Webb AE, McKay A, Pollina EA, et al. Lysosome activation clears aggregates and enhances quiescent neural stem cell activation during aging. Science. 2018;359:1277–83.29590078 10.1126/science.aag3048PMC5915358

[17] Fan Y, Mizoguchi Y, Tatematsu M, Linder MI, Frenz S, Choi J, et al. Analyzing mitochondrial respiration of human induced pluripotent stem cell-derived myeloid progenitors using Seahorse technology. STAR Protoc. 2023;4:102073.36853722 10.1016/j.xpro.2023.102073PMC9922929

[18] Sim SW, Jang Y, Park TS, Park BC, Lee YM, Jun HS. Molecular mechanisms of aberrant neutrophil differentiation in glycogen storage disease type Ib. Cell Mol Life Sci. 2022;79:246.35437689 10.1007/s00018-022-04267-5PMC11071875

[19] Cavnar PJ, Berthier E, Beebe DJ, Huttenlocher A. Hax1 regulates neutrophil adhesion and motility through RhoA. J Cell Biol. 2011;193:465–73.21518791 10.1083/jcb.201010143PMC3087009

[20] Radhika V, Onesime D, Ha JH, Dhanasekaran N. Galpha13 stimulates cell migration through cortactin-interacting protein Hax-1. J Biol Chem. 2004;279:49406–13.15339924 10.1074/jbc.M408836200

[21] Balcerak A, Trebinska-Stryjewska A, Wakula M, Chmielarczyk M, Smietanka U, Rubel T, et al. HAX1 impact on collective cell migration, cell adhesion, and cell shape is linked to the regulation of actomyosin contractility. Mol Biol Cell. 2019;30:3024–36.31644363 10.1091/mbc.E19-05-0304PMC6880882

[22] Grimes D, Johnson R, Pashos M, Cummings C, Kang C, Sampedro GR, et al. ORAI1 and ORAI2 modulate murine neutrophil calcium signaling, cellular activation, and host defense. Proc Natl Acad Sci USA. 2020;117:24403–14.32929002 10.1073/pnas.2008032117PMC7533673

[23] Thangaratnarajah C, Ruprecht JJ, Kunji ER. Calcium-induced conformational changes of the regulatory domain of human mitochondrial aspartate/glutamate carriers. Nat Commun. 2014;5:5491.25410934 10.1038/ncomms6491PMC4250520

[24] Mauvezin C, Nagy P, Juhasz G, Neufeld TP. Autophagosome-lysosome fusion is independent of V-ATPase-mediated acidification. Nat Commun. 2015;6:7007.25959678 10.1038/ncomms8007PMC4428688

[25] Vafiadaki E, Arvanitis DA, Pagakis SN, Papalouka V, Sanoudou D, Kontrogianni-Konstantopoulos A, et al. The anti-apoptotic protein HAX-1 interacts with SERCA2 and regulates its protein levels to promote cell survival. Mol Biol Cell. 2009;20:306–18.18971376 10.1091/mbc.E08-06-0587PMC2613088

[26] Ganley IG, Wong PM, Gammoh N, Jiang X. Distinct autophagosomal-lysosomal fusion mechanism revealed by thapsigargin-induced autophagy arrest. Mol Cell. 2011;42:731–43.21700220 10.1016/j.molcel.2011.04.024PMC3124681

[27] Vafiadaki E, Sanoudou D, Arvanitis DA, Catino DH, Kranias EG, Kontrogianni-Konstantopoulos A. Phospholamban interacts with HAX-1, a mitochondrial protein with anti-apoptotic function. J Mol Biol. 2007;367:65–79.17241641 10.1016/j.jmb.2006.10.057

[28] Zhao W, Waggoner JR, Zhang ZG, Lam CK, Han P, Qian J, et al. The anti-apoptotic protein HAX-1 is a regulator of cardiac function. Proc Natl Acad Sci USA. 2009;106:20776–81.19920172 10.1073/pnas.0906998106PMC2791603

[29] Balcerak A, Rowinski S, Szafron LM, Grzybowska EA. The calcium binding properties and structure prediction of the Hax-1 protein. Acta Biochim Pol. 2017;64:537–42.28859179 10.18388/abp.2017_1529

[30] Injarabian L, Devin A, Ransac S, Marteyn BS. Neutrophil metabolic shift during their lifecycle: impact on their survival and activation. Int J Mol Sci. 2019;21:287.31906243 10.3390/ijms21010287PMC6981538

[31] Dufer J, Biakou D, Joly P, Benoist H, Carpentier Y, Desplaces A. Quantitative morphological aspects of granulocytic differentiation induced in HL-60 cells by dimethylsulfoxide and retinoic acid. Leuk Res. 1989;13:621–7.2761293 10.1016/0145-2126(89)90131-8

[32] Martin SJ, Bradley JG, Cotter TG. HL-60 cells induced to differentiate towards neutrophils subsequently die via apoptosis. Clin Exp Immunol. 1990;79:448–53.2317949 10.1111/j.1365-2249.1990.tb08110.xPMC1534969

[33] Hu H, Shikama Y, Matsuoka I, Kimura J. Terminally differentiated neutrophils predominantly express Survivin-2 alpha, a dominant-negative isoform of survivin. J Leukoc Biol. 2008;83:393–400.17965335 10.1189/jlb.0507282

[34] Bhakta SB, Lundgren SM, Sesti BN, Flores BA, Akdogan E, Collins SR, et al. Neutrophil-like cells derived from the HL-60 cell-line as a genetically-tractable model for neutrophil degranulation. PLoS One. 2024;19:e0297758.38324578 10.1371/journal.pone.0297758PMC10849234

[35] Wisniewski JR. Filter aided sample preparation - a tutorial. Anal Chim Acta. 2019;1090:23–30.31655642 10.1016/j.aca.2019.08.032

[36] Tyanova S, Temu T, Cox J. The MaxQuant computational platform for mass spectrometry-based shotgun proteomics. Nat Protoc. 2016;11:2301–19.27809316 10.1038/nprot.2016.136

[37] Tyanova S, Temu T, Sinitcyn P, Carlson A, Hein MY, Geiger T, et al. The Perseus computational platform for comprehensive analysis of (prote)omics data. Nat Methods. 2016;13:731–40.27348712 10.1038/nmeth.3901

[38] Plubell DL, Wilmarth PA, Zhao Y, Fenton AM, Minnier J, Reddy AP, et al. Extended Multiplexing of Tandem Mass Tags (TMT) labeling reveals age and high fat diet specific proteome changes in mouse epididymal adipose tissue. Mol Cell Proteomics. 2017;16:873–90.28325852 10.1074/mcp.M116.065524PMC5417827

[39] Sherman BT, Hao M, Qiu J, Jiao X, Baseler MW, Lane HC, et al. DAVID: a web server for functional enrichment analysis and functional annotation of gene lists (2021 update). Nucleic Acids Res. 2022;50:W216–W221.35325185 10.1093/nar/gkac194PMC9252805

[40] Huang da W, Sherman BT, Lempicki RA. Systematic and integrative analysis of large gene lists using DAVID bioinformatics resources. Nat Protoc. 2009;4:44–57.19131956 10.1038/nprot.2008.211

[41] Chen EY, Tan CM, Kou Y, Duan Q, Wang Z, Meirelles GV, et al. Enrichr: interactive and collaborative HTML5 gene list enrichment analysis tool. BMC Bioinformatics. 2013;14:128.23586463 10.1186/1471-2105-14-128PMC3637064

[42] Xie Z, Bailey A, Kuleshov MV, Clarke DJB, Evangelista JE, Jenkins SL, et al. Gene set knowledge discovery with Enrichr. Curr Protoc. 2021;1:e90.33780170 10.1002/cpz1.90PMC8152575

[43] Berndt N, Bulik S, Wallach I, Wunsch T, Konig M, Stockmann M, et al. HEPATOKIN1 is a biochemistry-based model of liver metabolism for applications in medicine and pharmacology. Nat Commun. 2018;9:2386.29921957 10.1038/s41467-018-04720-9PMC6008457

[44] Wallstab C, Eleftheriadou D, Schulz T, Damm G, Seehofer D, Borlak J, et al. A unifying mathematical model of lipid droplet metabolism reveals key molecular players in the development of hepatic steatosis. FEBS J. 2017;284:3245–61.28763157 10.1111/febs.14189

[45] Berndt N, Eckstein J, Wallach I, Nordmeyer S, Kelm M, Kirchner M, et al. CARDIOKIN1: computational assessment of myocardial metabolic capability in healthy controls and patients with valve diseases. Circulation. 2021;144:1926–39.34762513 10.1161/CIRCULATIONAHA.121.055646PMC8663543

[46] Bulik S, Holzhutter HG, Berndt N. The relative importance of kinetic mechanisms and variable enzyme abundances for the regulation of hepatic glucose metabolism–insights from mathematical modeling. BMC Biol. 2016;14:15.26935066 10.1186/s12915-016-0237-6PMC4774192

[47] Hornstein T, Unfried K. Protocol for the differentiation of HL-60 cells into a neutrophil-like state. STAR Protoc. 2025;6:104135.41075254 10.1016/j.xpro.2025.104135PMC12547236

