# Supplementary material for: Granulocyte differentiation arrest in HAX1-deficient cells, demonstrated in a new in vitro model of a certain phenotypic aspects of Kostmann disease, is caused by ineffective lipid droplet autophagy and fatty acids uptake
Source: Cell Death Dis. 2026 May 5;17(1):594. doi: 10.1038/s41419-026-08805-y (PMC13287692; doi:10.1038/s41419-026-08805-y)

Figure S1. PCA Analysis of iTRAQ results from the wild type MCF7 cells (green) and *HAX1* KO#2 cells (red), each dot representing one biological repeat.

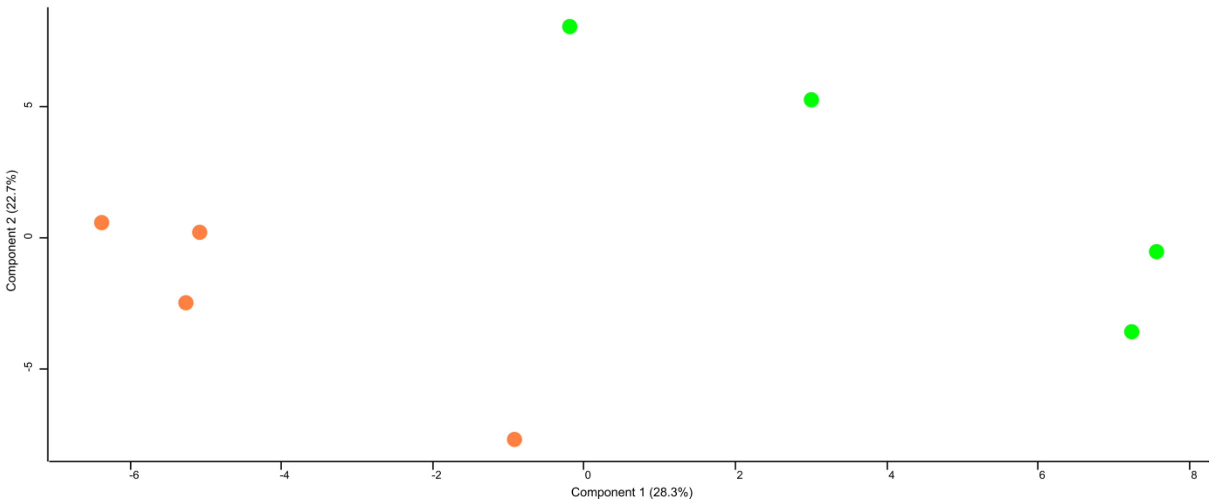

Figure S2. Western blot showing HAX1 protein expression level in WT and the two CRISPR/Cas9 HAX1 knockout cell lines (KO#1 and #2). Cell line generation was described elsewhere (13). Reference: α-tubulin.

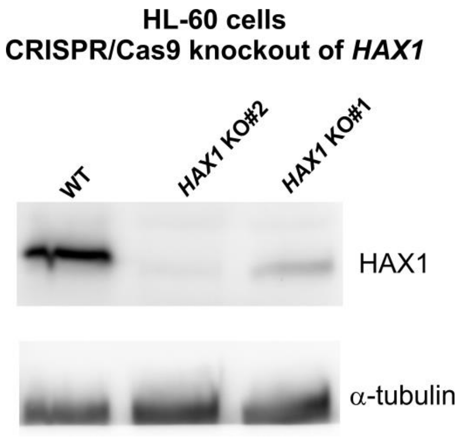

Figure S3. Pathway analysis of proteomic data reveals differences in pathway ranking depending on the method of analysis, with QSM analysis pointing decisively to lipid metabolism as the main group.

A. Reactome (Enrichr) pathway analysis reveals differences in neutrophil degranulation, Rho GTPases activity, metabolism of amino acids, mitochondrial translation, mitochondrial respiration and lipid metabolism. Ranked by –log10(p-value). B. QSM analysis. Venn diagram showing significantly regulated functional markers in HAX1 WT/KOs, with numbers and category, revealing changes in fatty acid utilization and TAG synthesis and storage.

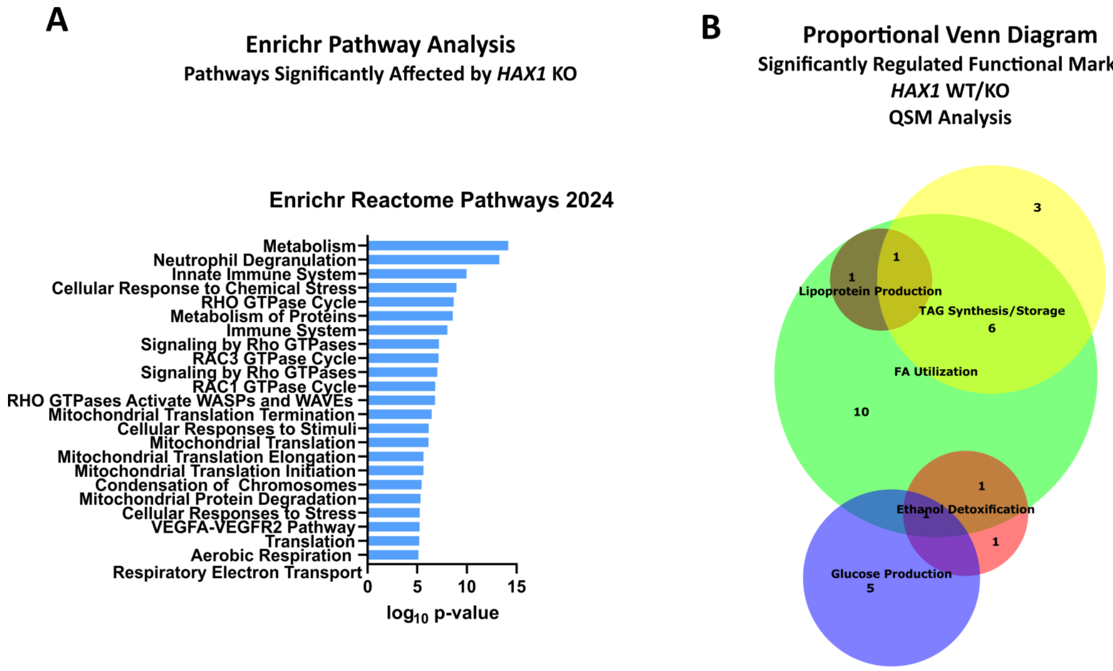

Figure S4. Flow cytometry results showing CD11b and CD33 expression in HL-60 *HAX1* WT and HL-60 *HAX1* KO cells in consecutive biological repeats for non-induced cells and cells induced to differentiation after 72h incubation with ATRA and ATRA+FAs mix.

A. HL-60 WT , B. HL-60 *HAX1* KO#1 C.HL-60 *HAX1* KO#2

1. Untreated
2. low DMSO
3. FAs
4. ATRA
5. ATRA+FAs

Figure S4A1

HL-60 WT

Untreated 1

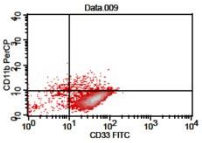

Quadrant Statistics

File: Data.009 | Log Data Units: Linear Values
Sample ID: | Patient ID:
Tube: Untitled | Panel: Untitled Acquisition Tube List
Acquisition Date: 11-Apr-24 | Gate: G1
Gated Events: 46728 | Total Events: 50000
X Parameter: CD33 FITC (Log) | Y Parameter: CD11b PerCP (Log)
Quad Location: 10, 8

Quad	Events	% Gated	% Total	X Mean	X Geo Mean	Y Mean	Y Geo Mean
UL	137	0.29	0.27	6.95	6.44	14.95	13.44
UR	1015	2.17	2.03	60.87	52.53	11.63	10.74
LL	425	0.91	0.85	2.59	1.74	2.51	1.98
LR	45151	96.63	90.30	43.04	41.41	4.81	4.64

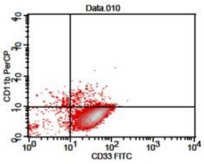

Quadrant Statistics

File: Data.010 | Log Data Units: Linear Values
Sample ID: | Patient ID:
Tube: Untitled | Panel: Untitled Acquisition Tube List
Acquisition Date: 11-Apr-24 | Gate: G1
Gated Events: 45479 | Total Events: 50000
X Parameter: CD33 FITC (Log) | Y Parameter: CD11b PerCP (Log)
Quad Location: 10, 8

Quad	Events	% Gated	% Total	X Mean	X Geo Mean	Y Mean	Y Geo Mean
UL	113	0.25	0.23	6.20	5.55	15.88	14.69
UR	1217	2.68	2.43	56.41	50.45	11.83	10.63
LL	577	1.27	1.15	2.14	1.47	1.95	1.63
LR	43572	95.81	87.14	40.79	39.20	5.02	4.85

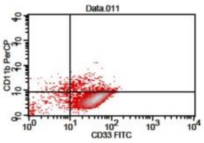

Quadrant Statistics

File: Data.011 | Log Data Units: Linear Values
Sample ID: | Patient ID:
Tube: Untitled | Panel: Untitled Acquisition Tube List
Acquisition Date: 11-Apr-24 | Gate: G1
Gated Events: 46262 | Total Events: 50000
X Parameter: CD33 FITC (Log) | Y Parameter: CD11b PerCP (Log)
Quad Location: 10, 8

Quad	Events	% Gated	% Total	X Mean	X Geo Mean	Y Mean	Y Geo Mean
UL	125	0.27	0.25	6.28	5.63	14.91	13.74
UR	1339	2.89	2.68	58.53	51.56	11.73	10.70
LL	572	1.24	1.14	2.36	1.60	2.35	1.90
LR	44226	95.60	88.45	42.22	40.64	4.96	4.79

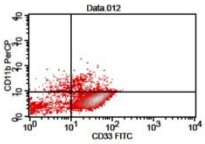

Quadrant Statistics

File: Data.012 | Log Data Units: Linear Values
Sample ID: | Patient ID:
Tube: Untitled | Panel: Untitled Acquisition Tube List
Acquisition Date: 11-Apr-24 | Gate: G1
Gated Events: 43647 | Total Events: 50000
X Parameter: CD33 FITC (Log) | Y Parameter: CD11b PerCP (Log)
Quad Location: 10, 8

Quad	Events	% Gated	% Total	X Mean	X Geo Mean	Y Mean	Y Geo Mean
UL	218	0.50	0.44	6.12	5.54	17.02	15.11
UR	1499	3.43	3.00	52.91	45.44	12.75	11.23
LL	1938	4.44	3.88	1.73	1.35	1.97	1.76
LR	39992	91.63	79.98	42.40	40.78	5.14	4.97

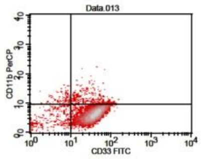

Quadrant Statistics **1**

File: Data.013 | Log Data Units: Linear Values
Sample ID: | Patient ID:
Tube: Untitled | Panel: Untitled Acquisition Tube List
Acquisition Date: 11-Apr-24 | Gate: G1
Gated Events: 45497 | Total Events: 50000
X Parameter: CD33 FITC (Log) | Y Parameter: CD11b PerCP (Log)
Quad Location: 10, 8

Quad	Events	% Gated	% Total	X Mean	X Geo Mean	Y Mean	Y Geo Mean
UL	161	0.35	0.32	6.78	6.27	14.71	13.68
UR	754	1.66	1.51	54.37	46.38	12.88	11.26
LL	925	2.03	1.85	2.06	1.46	1.93	1.61
LR	43657	95.96	87.31	40.12	38.46	4.61	4.43

Untreated 2

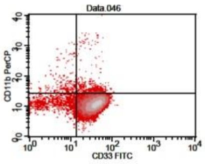

Quadrant Statistics

File: Data.046 | Log Data Units: Linear Values
Sample ID: | Patient ID:
Tube: Untitled | Panel: Untitled Acquisition Tube List
Acquisition Date: 11-Oct-24 | Gate: G1
Gated Events: 40655 | Total Events: 50000
X Parameter: CD33 FITC (Log) | Y Parameter: CD11b PerCP (Log)
Quad Location: 13, 25

Quad	Events	% Gated	% Total	X Mean	X Geo Mean	Y Mean	Y Geo Mean
UL	267	0.66	0.53	7.99	7.07	97.69	53.92
UR	883	2.17	1.77	38.34	35.21	103.50	39.30
LL	1154	2.84	2.31	6.48	4.67	12.98	12.12
LR	38351	94.33	76.70	39.43	37.65	12.53	11.96

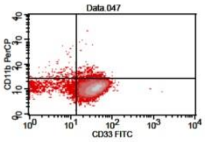

Quadrant Statistics

File: Data.047 | Log Data Units: Linear Values
Sample ID: | Patient ID:
Tube: Untitled | Panel: Untitled Acquisition Tube List
Acquisition Date: 11-Oct-24 | Gate: G1
Gated Events: 40019 | Total Events: 50000
X Parameter: CD33 FITC (Log) | Y Parameter: CD11b PerCP (Log)
Quad Location: 13, 25

Quad	Events	% Gated	% Total	X Mean	X Geo Mean	Y Mean	Y Geo Mean
UL	184	0.46	0.37	7.27	6.20	56.47	43.16
UR	875	2.19	1.75	36.68	33.79	40.34	33.39
LL	1139	2.85	2.28	6.32	4.35	12.99	12.09
LR	37821	94.51	75.64	38.80	36.93	12.35	11.77

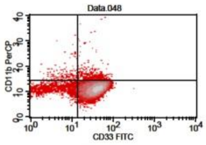

Quadrant Statistics

File: Data.048 | Log Data Units: Linear Values
Sample ID: | Patient ID:
Tube: Untitled | Panel: Untitled Acquisition Tube List
Acquisition Date: 11-Oct-24 | Gate: G1
Gated Events: 40586 | Total Events: 50000
X Parameter: CD33 FITC (Log) | Y Parameter: CD11b PerCP (Log)
Quad Location: 13, 25

Quad	Events	% Gated	% Total	X Mean	X Geo Mean	Y Mean	Y Geo Mean
UL	235	0.58	0.47	7.46	6.55	65.34	43.84
UR	1286	3.17	2.57	40.78	37.47	58.80	33.43
LL	1337	3.29	2.67	6.46	4.82	13.92	13.19
LR	37728	92.96	75.46	38.99	37.18	13.55	12.93

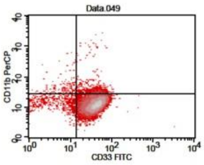

Quadrant Statistics

File: Data.049 | Log Data Units: Linear Values
Sample ID: | Patient ID:
Tube: Untitled | Panel: Untitled Acquisition Tube List
Acquisition Date: 11-Oct-24 | Gate: G1
Gated Events: 33205 | Total Events: 39840
X Parameter: CD33 FITC (Log) | Y Parameter: CD11b PerCP (Log)
Quad Location: 13, 25

Quad	Events	% Gated	% Total	X Mean	X Geo Mean	Y Mean	Y Geo Mean
UL	178	0.54	0.45	7.44	6.38	59.72	44.86
UR	1184	3.57	2.97	44.55	40.91	54.81	34.56
LL	598	1.80	1.50	6.66	4.69	13.68	12.69
LR	31245	94.10	78.43	41.47	39.46	12.84	12.22

Untreated 3

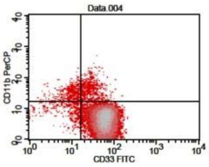

Quadrant Statistics

File: Data.004 | Log Data Units: Linear Values
Sample ID: | Patient ID:
Tube: Untitled | Panel: Untitled Acquisition Tube List
Acquisition Date: 12-Sep-24 | Gate: G1
Gated Events: 32161 | Total Events: 41250
X Parameter: CD33 FITC (Log) | Y Parameter: CD11b PerCP (Log)
Quad Location: 16, 16

Quad	Events	% Gated	% Total	X Mean	X Geo Mean	Y Mean	Y Geo Mean
UL	574	1.78	1.39	10.20	9.42	35.89	32.35
UR	752	2.34	1.82	31.59	28.72	49.34	40.90
LL	331	1.03	0.80	9.21	7.49	8.74	7.73
LR	30504	94.85	73.95	61.20	57.98	4.93	4.18

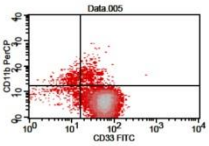

Quadrant Statistics

File: Data.005 | Log Data Units: Linear Values
Sample ID: | Patient ID:
Tube: Untitled | Panel: Untitled Acquisition Tube List
Acquisition Date: 12-Sep-24 | Gate: G1
Gated Events: 28357 | Total Events: 37665
X Parameter: CD33 FITC (Log) | Y Parameter: CD11b PerCP (Log)
Quad Location: 16, 16

Quad	Events	% Gated	% Total	X Mean	X Geo Mean	Y Mean	Y Geo Mean
UL	521	1.84	1.38	10.46	9.77	38.89	33.91
UR	720	2.54	1.91	32.39	28.74	57.16	42.92
LL	391	1.38	1.04	9.25	7.53	8.68	7.67
LR	26725	94.24	70.95	60.26	56.68	4.68	4.01

Untreated 4

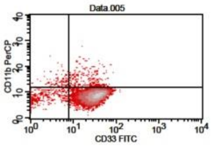

Quadrant Statistics

File: Data.005 | Log Data Units: Linear Values
Sample ID: | Patient ID:
Tube: Untitled | Panel: Untitled Acquisition Tube List
Acquisition Date: 02-Jul-24 | Gate: G1
Gated Events: 26800 | Total Events: 32205
X Parameter: CD33 FITC (Log) | Y Parameter: CD11b PerCP (Log)
Quad Location: 8, 14

Quad	Events	% Gated	% Total	X Mean	X Geo Mean	Y Mean	Y Geo Mean
UL	108	0.40	0.34	4.61	4.14	32.60	27.15
UR	619	2.31	1.92	32.31	28.34	27.64	21.44
LL	603	2.25	1.87	1.80	1.43	6.14	5.69
LR	25470	95.04	79.09	34.81	33.11	8.15	7.85

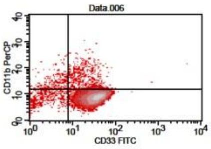

Quadrant Statistics

File: Data.006 | Log Data Units: Linear Values
Sample ID: | Patient ID:
Tube: Untitled | Panel: Untitled Acquisition Tube List
Acquisition Date: 02-Jul-24 | Gate: G1
Gated Events: 41278 | Total Events: 50000
X Parameter: CD33 FITC (Log) | Y Parameter: CD11b PerCP (Log)
Quad Location: 8, 14

Quad	Events	% Gated	% Total	X Mean	X Geo Mean	Y Mean	Y Geo Mean
UL	332	0.80	0.66	4.34	3.89	36.82	29.12
UR	966	2.34	1.93	34.32	25.08	43.79	28.37
LL	1000	2.42	2.00	1.81	1.43	5.62	5.05
LR	38980	94.43	77.96	36.05	34.37	8.07	7.80

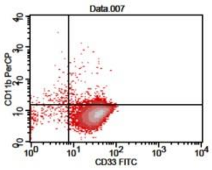

Quadrant Statistics

File: Data.007 | Log Data Units: Linear Values
Sample ID: | Patient ID:
Tube: Untitled | Panel: Untitled Acquisition Tube List
Acquisition Date: 02-Jul-24 | Gate: G1
Gated Events: 41334 | Total Events: 47865
X Parameter: CD33 FITC (Log) | Y Parameter: CD11b PerCP (Log)
Quad Location: 8, 14

Quad	Events	% Gated	% Total	X Mean	X Geo Mean	Y Mean	Y Geo Mean
UL	114	0.28	0.24	4.48	3.96	71.92	33.66
UR	939	2.27	1.96	34.75	31.39	29.74	19.78
LL	456	1.10	0.95	1.74	1.37	6.07	5.47
LR	39825	96.35	83.20	36.34	34.79	7.94	7.65

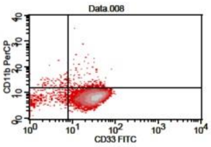

Quadrant Statistics

File: Data.008 | Log Data Units: Linear Values
Sample ID: | Patient ID:
Tube: Untitled | Panel: Untitled Acquisition Tube List
Acquisition Date: 02-Jul-24 | Gate: G1
Gated Events: 28662 | Total Events: 34920
X Parameter: CD33 FITC (Log) | Y Parameter: CD11b PerCP (Log)
Quad Location: 8, 14

Quad	Events	% Gated	% Total	X Mean	X Geo Mean	Y Mean	Y Geo Mean
UL	79	0.28	0.23	4.55	4.05	33.26	28.45
UR	482	1.68	1.38	30.20	27.10	31.95	19.87
LL	694	2.42	1.99	1.86	1.43	5.33	4.88
LR	27407	95.62	78.49	33.58	31.88	7.47	7.19

Figure S4A2

HL-60 WT

DMSO, non-induced (1)

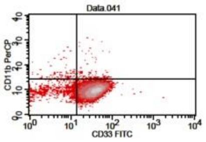

Quadrant Statistics

File: Data.041
Sample ID:
Tube: Untitled
Acquisition Date: 11-Oct-24
Gated Events: 35581
X Parameter: CD33 FITC (Log)
Quad Location: 13, 25

Log Data Units: Linear Values
Patient ID:
Panel: Untitled Acquisition Tube List
Gate: G1
Total Events: 40020
Y Parameter: CD11b PerCP (Log)

Quad	Events	% Gated	% Total	X Mean	X Geo Mean	Y Mean	Y Geo Mean
UL	104	0.29	0.26	6.99	6.03	64.03	45.29
UR	206	0.58	0.51	40.24	36.23	68.96	39.03
LL	968	2.72	2.42	6.07	3.94	9.57	8.80
LR	34303	96.41	85.71	38.98	36.90	10.28	9.80

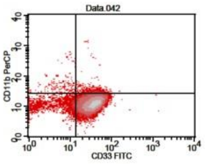

Quadrant Statistics

File: Data.042
Sample ID:
Tube: Untitled
Acquisition Date: 11-Oct-24
Gated Events: 43973
X Parameter: CD33 FITC (Log)
Quad Location: 13, 25

Log Data Units: Linear Values
Patient ID:
Panel: Untitled Acquisition Tube List
Gate: G1
Total Events: 50000
Y Parameter: CD11b PerCP (Log)

Quad	Events	% Gated	% Total	X Mean	X Geo Mean	Y Mean	Y Geo Mean
UL	189	0.43	0.38	7.15	6.12	71.61	48.97
UR	928	2.11	1.86	41.51	38.19	40.81	31.11
LL	1586	3.61	3.17	6.50	4.36	11.52	10.74
LR	41270	93.85	82.54	36.61	34.81	12.72	12.13

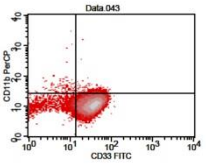

Quadrant Statistics

File: Data.043
Sample ID:
Tube: Untitled
Acquisition Date: 11-Oct-24
Gated Events: 40226
X Parameter: CD33 FITC (Log)
Quad Location: 13, 25

Log Data Units: Linear Values
Patient ID:
Panel: Untitled Acquisition Tube List
Gate: G1
Total Events: 45270
Y Parameter: CD11b PerCP (Log)

Quad	Events	% Gated	% Total	X Mean	X Geo Mean	Y Mean	Y Geo Mean
UL	143	0.36	0.32	7.28	6.47	85.49	45.32
UR	578	1.44	1.28	40.44	37.61	34.11	30.60
LL	1723	4.28	3.81	6.08	4.07	11.44	10.70
LR	37782	93.92	83.46	36.44	34.79	11.83	11.29

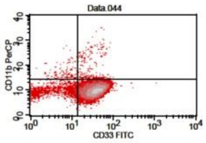

Quadrant Statistics

File: Data.044
Sample ID:
Tube: Untitled
Acquisition Date: 11-Oct-24
Gated Events: 38454
X Parameter: CD33 FITC (Log)
Quad Location: 13, 25

Log Data Units: Linear Values
Patient ID:
Panel: Untitled Acquisition Tube List
Gate: G1
Total Events: 43605
Y Parameter: CD11b PerCP (Log)

Quad	Events	% Gated	% Total	X Mean	X Geo Mean	Y Mean	Y Geo Mean
UL	128	0.33	0.29	7.06	6.28	68.28	51.76
UR	354	0.92	0.81	39.59	35.43	131.70	54.34
LL	1256	3.27	2.88	5.74	3.77	9.86	9.13
LR	36716	95.48	84.20	37.90	36.17	10.61	10.12

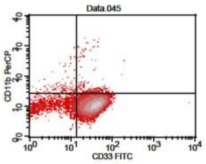

Quadrant Statistics

File: Data.045
Sample ID:
Tube: Untitled
Acquisition Date: 11-Oct-24
Gated Events: 43603
X Parameter: CD33 FITC (Log)
Quad Location: 13, 25

Log Data Units: Linear Values
Patient ID:
Panel: Untitled Acquisition Tube List
Gate: G1
Total Events: 50000
Y Parameter: CD11b PerCP (Log)

Quad	Events	% Gated	% Total	X Mean	X Geo Mean	Y Mean	Y Geo Mean
UL	155	0.36	0.31	7.24	6.28	67.20	45.99
UR	561	1.29	1.12	41.70	37.94	73.90	36.24
LL	1693	3.88	3.39	5.72	3.87	11.10	10.38
LR	41194	94.48	82.39	37.50	35.59	11.83	11.30

Figure S4A3

HL-60 WT

FAs (1)

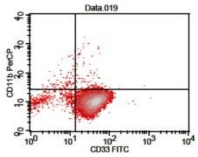

Quadrant Statistics

File: Data.019
Sample ID:
Tube: Untitled
Acquisition Date: 11-Oct-24
Gated Events: 45301
X Parameter: CD33 FITC (Log)
Quad Location: 13, 25

Log Data Units: Linear Values
Patient ID:
Panel: Untitled Acquisition Tube List
Gate: G1
Total Events: 50000
Y Parameter: CD11b PerCP (Log)

Quad	Events	% Gated	% Total	X Mean	X Geo Mean	Y Mean	Y Geo Mean
UL	111	0.25	0.22	7.53	6.68	67.22	48.58
UR	270	0.60	0.54	43.14	37.23	79.78	41.50
LL	490	1.08	0.98	5.54	3.57	11.55	10.69
LR	44430	98.08	88.86	44.25	42.32	10.86	10.39

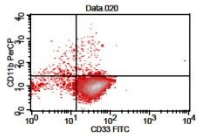

Quadrant Statistics

File: Data.020
Sample ID:
Tube: Untitled
Acquisition Date: 11-Oct-24
Gated Events: 44855
X Parameter: CD33 FITC (Log)
Quad Location: 13, 25

Log Data Units: Linear Values
Patient ID:
Panel: Untitled Acquisition Tube List
Gate: G1
Total Events: 50000
Y Parameter: CD11b PerCP (Log)

Quad	Events	% Gated	% Total	X Mean	X Geo Mean	Y Mean	Y Geo Mean
UL	189	0.42	0.38	6.63	5.73	101.27	62.59
UR	358	0.80	0.72	57.98	34.82	146.98	57.79
LL	531	1.18	1.06	5.67	3.58	12.11	11.22
LR	43777	97.60	87.55	43.59	41.65	10.62	10.14

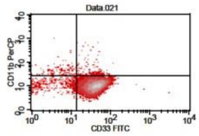

Quadrant Statistics

File: Data.021
Sample ID:
Tube: Untitled
Acquisition Date: 11-Oct-24
Gated Events: 45335
X Parameter: CD33 FITC (Log)
Quad Location: 13, 25

Log Data Units: Linear Values
Patient ID:
Panel: Untitled Acquisition Tube List
Gate: G1
Total Events: 50000
Y Parameter: CD11b PerCP (Log)

Quad	Events	% Gated	% Total	X Mean	X Geo Mean	Y Mean	Y Geo Mean
UL	158	0.35	0.32	6.64	5.59	120.05	47.81
UR	459	1.01	0.92	40.51	37.36	43.62	33.96
LL	525	1.16	1.05	6.03	3.98	12.32	11.44
LR	44193	97.48	88.39	43.86	41.94	11.21	10.74

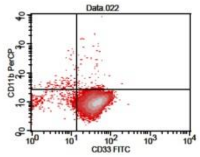

Quadrant Statistics

File: Data.022
Sample ID:
Tube: Untitled
Acquisition Date: 11-Oct-24
Gated Events: 45348
X Parameter: CD33 FITC (Log)
Quad Location: 13, 25

Log Data Units: Linear Values
Patient ID:
Panel: Untitled Acquisition Tube List
Gate: G1
Total Events: 50000
Y Parameter: CD11b PerCP (Log)

Quad	Events	% Gated	% Total	X Mean	X Geo Mean	Y Mean	Y Geo Mean
UL	115	0.25	0.23	6.70	5.71	75.64	50.53
UR	206	0.45	0.41	38.15	34.96	143.51	45.58
LL	433	0.95	0.87	5.83	3.60	11.33	10.41
LR	44594	98.34	89.19	43.95	42.09	9.88	9.46

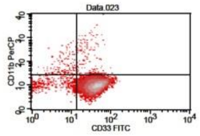

Quadrant Statistics

File: Data.023
Sample ID:
Tube: Untitled
Acquisition Date: 11-Oct-24
Gated Events: 44894
X Parameter: CD33 FITC (Log)
Quad Location: 13, 25

Log Data Units: Linear Values
Patient ID:
Panel: Untitled Acquisition Tube List
Gate: G1
Total Events: 50000
Y Parameter: CD11b PerCP (Log)

Quad	Events	% Gated	% Total	X Mean	X Geo Mean	Y Mean	Y Geo Mean
UL	168	0.37	0.34	6.73	5.78	114.96	71.16
UR	358	0.80	0.72	38.15	34.95	188.42	57.00
LL	520	1.16	1.04	5.65	3.55	11.88	11.10
LR	43848	97.67	87.70	44.01	42.13	10.70	10.22

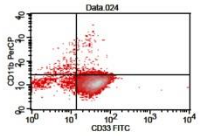

Quadrant Statistics

File: Data.024
Sample ID:
Tube: Untitled
Acquisition Date: 11-Oct-24
Gated Events: 44869
X Parameter: CD33 FITC (Log)
Quad Location: 13, 25

Log Data Units: Linear Values
Patient ID:
Panel: Untitled Acquisition Tube List
Gate: G1
Total Events: 50000
Y Parameter: CD11b PerCP (Log)

Quad	Events	% Gated	% Total	X Mean	X Geo Mean	Y Mean	Y Geo Mean
UL	219	0.49	0.44	6.85	6.01	90.99	58.64
UR	946	2.11	1.89	41.78	37.82	76.63	37.22
LL	655	1.46	1.31	6.19	4.41	13.92	13.11
LR	43049	95.94	86.10	44.42	42.21	13.02	12.52

Figure S4A4

HL-60 WT
ATRA 58μM 72h

ATRA (1)

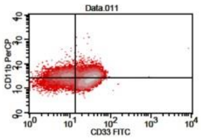

Quadrant Statistics

File: Data.011 | Log Data Units: Linear Values
Sample ID: | Patient ID:
Tube: Untitled | Panel: Untitled Acquisition Tube List
Acquisition Date: 11-Oct-24 | Gate: G1
Gated Events: 39513 | Total Events: 50000
X Parameter: CD33 FITC (Log) | Y Parameter: CD11b PerCP (Log)
Quad Location: 13, 25

Quad	Events	% Gated	% Total	X Mean	X Geo Mean	Y Mean	Y Geo Mean
UL	6822	17.27	13.64	7.90	7.38	39.19	37.48
UR	15338	38.82	30.68	34.91	32.72	36.32	34.52
LL	2652	6.71	5.30	7.26	6.30	18.44	17.78
LR	14701	37.21	29.40	27.14	26.10	19.13	18.70

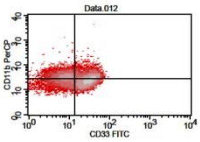

Quadrant Statistics

File: Data.012 | Log Data Units: Linear Values
Sample ID: | Patient ID:
Tube: Untitled | Panel: Untitled Acquisition Tube List
Acquisition Date: 11-Oct-24 | Gate: G1
Gated Events: 38861 | Total Events: 50000
X Parameter: CD33 FITC (Log) | Y Parameter: CD11b PerCP (Log)
Quad Location: 13, 25

Quad	Events	% Gated	% Total	X Mean	X Geo Mean	Y Mean	Y Geo Mean
UL	7619	19.61	15.24	7.91	7.37	39.16	37.20
UR	14245	36.66	28.49	33.67	31.92	36.31	34.49
LL	3484	8.97	6.97	7.51	6.53	18.29	17.65
LR	13513	34.77	27.03	26.41	25.36	19.22	18.81

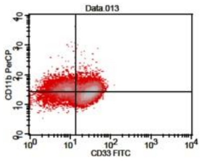

Quadrant Statistics

File: Data.013 | Log Data Units: Linear Values
Sample ID: | Patient ID:
Tube: Untitled | Panel: Untitled Acquisition Tube List
Acquisition Date: 11-Oct-24 | Gate: G1
Gated Events: 39607 | Total Events: 50000
X Parameter: CD33 FITC (Log) | Y Parameter: CD11b PerCP (Log)
Quad Location: 13, 25

Quad	Events	% Gated	% Total	X Mean	X Geo Mean	Y Mean	Y Geo Mean
UL	7858	19.84	15.72	7.81	7.28	41.10	38.07
UR	15115	38.16	30.23	33.35	31.76	36.53	34.84
LL	2806	7.08	5.61	7.15	6.15	18.65	18.00
LR	13828	34.91	27.66	26.50	25.49	19.31	18.91

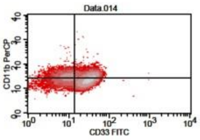

Quadrant Statistics

File: Data.014 | Log Data Units: Linear Values
Sample ID: | Patient ID:
Tube: Untitled | Panel: Untitled Acquisition Tube List
Acquisition Date: 11-Oct-24 | Gate: G1
Gated Events: 40802 | Total Events: 50000
X Parameter: CD33 FITC (Log) | Y Parameter: CD11b PerCP (Log)
Quad Location: 13, 25

Quad	Events	% Gated	% Total	X Mean	X Geo Mean	Y Mean	Y Geo Mean
UL	6281	15.39	12.56	8.41	7.88	37.84	36.13
UR	14721	36.08	29.44	34.26	32.36	35.48	33.98
LL	3666	8.98	7.33	7.74	6.84	18.45	17.84
LR	16134	39.54	32.27	27.54	26.36	19.02	18.58

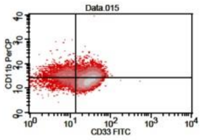

Quadrant Statistics

File: Data.015 | Log Data Units: Linear Values
Sample ID: | Patient ID:
Tube: Untitled | Panel: Untitled Acquisition Tube List
Acquisition Date: 11-Oct-24 | Gate: G1
Gated Events: 42012 | Total Events: 50000
X Parameter: CD33 FITC (Log) | Y Parameter: CD11b PerCP (Log)
Quad Location: 13, 25

Quad	Events	% Gated	% Total	X Mean	X Geo Mean	Y Mean	Y Geo Mean
UL	4529	10.78	9.06	7.79	7.18	43.97	40.70
UR	15580	37.08	31.16	33.65	31.97	37.20	34.90
LL	2051	4.88	4.10	8.49	7.51	17.80	17.14
LR	19852	47.25	39.70	27.21	26.09	18.55	18.09

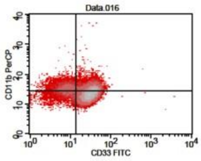

Quadrant Statistics

File: Data.016 | Log Data Units: Linear Values
Sample ID: | Patient ID:
Tube: Untitled | Panel: Untitled Acquisition Tube List
Acquisition Date: 11-Oct-24 | Gate: G1
Gated Events: 41305 | Total Events: 50000
X Parameter: CD33 FITC (Log) | Y Parameter: CD11b PerCP (Log)
Quad Location: 13, 25

Quad	Events	% Gated	% Total	X Mean	X Geo Mean	Y Mean	Y Geo Mean
UL	4733	11.46	9.47	7.89	7.31	40.32	38.03
UR	15108	36.58	30.22	34.62	32.92	37.79	34.77
LL	2178	5.27	4.36	7.76	6.74	18.40	17.73
LR	19286	46.69	38.57	28.58	27.05	18.29	17.80

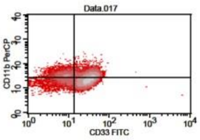

Quadrant Statistics

File: Data.017 | Log Data Units: Linear Values
Sample ID: | Patient ID:
Tube: Untitled | Panel: Untitled Acquisition Tube List
Acquisition Date: 11-Oct-24 | Gate: G1
Gated Events: 41893 | Total Events: 50000
X Parameter: CD33 FITC (Log) | Y Parameter: CD11b PerCP (Log)
Quad Location: 13, 25

Quad	Events	% Gated	% Total	X Mean	X Geo Mean	Y Mean	Y Geo Mean
UL	4918	11.74	9.84	8.03	7.50	39.28	37.58
UR	17400	41.53	34.80	34.47	32.84	35.75	34.38
LL	2732	6.52	5.46	7.64	6.58	18.09	17.45
LR	16843	40.20	33.69	27.28	25.92	19.22	18.81

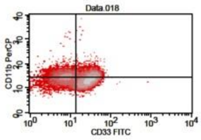

Quadrant Statistics

File: Data.018 | Log Data Units: Linear Values
Sample ID: | Patient ID:
Tube: Untitled | Panel: Untitled Acquisition Tube List
Acquisition Date: 11-Oct-24 | Gate: G1
Gated Events: 40123 | Total Events: 50000
X Parameter: CD33 FITC (Log) | Y Parameter: CD11b PerCP (Log)
Quad Location: 13, 25

Quad	Events	% Gated	% Total	X Mean	X Geo Mean	Y Mean	Y Geo Mean
UL	6309	15.72	12.62	7.61	7.07	39.42	36.09
UR	12434	30.99	24.87	33.16	31.66	36.26	33.62
LL	3913	9.75	7.83	7.33	6.44	18.06	17.35
LR	17467	43.53	34.93	26.56	25.11	18.55	18.07

ATRA (2)

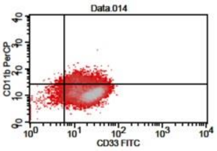

Quadrant Statistics

File: Data.014 | Log Data Units: Linear Values
Sample ID: | Patient ID:
Tube: Untitled | Panel: Untitled Acquisition Tube List
Acquisition Date: 11-Apr-24 | Gate: G1
Gated Events: 23219 | Total Events: 28425
X Parameter: CD33 FITC (Log) | Y Parameter: CD11b PerCP (Log)
Quad Location: 6, 24

Quad	Events	% Gated	% Total	X Mean	X Geo Mean	Y Mean	Y Geo Mean
UL	122	0.53	0.43	4.73	4.61	33.62	32.51
UR	3140	13.52	11.05	19.79	16.41	38.52	36.25
LL	635	2.73	2.23	4.03	3.65	11.04	9.49
LR	19322	83.22	67.98	24.08	21.61	12.67	11.89

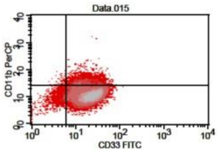

Quadrant Statistics

File: Data.015 | Log Data Units: Linear Values
Sample ID: | Patient ID:
Tube: Untitled | Panel: Untitled Acquisition Tube List
Acquisition Date: 11-Apr-24 | Gate: G1
Gated Events: 43609 | Total Events: 50000
X Parameter: CD33 FITC (Log) | Y Parameter: CD11b PerCP (Log)
Quad Location: 6, 24

Quad	Events	% Gated	% Total	X Mean	X Geo Mean	Y Mean	Y Geo Mean
UL	63	0.14	0.13	4.68	4.58	38.23	35.98
UR	3867	8.87	7.73	21.23	17.96	37.66	34.77
LL	949	2.18	1.90	4.14	3.86	10.27	9.02
LR	38730	88.81	77.46	22.06	20.00	12.12	11.36

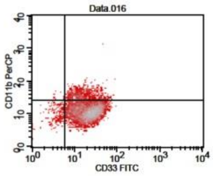

Quadrant Statistics

File: Data.016 | Log Data Units: Linear Values
Sample ID: | Patient ID:
Tube: Untitled | Panel: Untitled Acquisition Tube List
Acquisition Date: 11-Apr-24 | Gate: G1
Gated Events: 7615 | Total Events: 8865
X Parameter: CD33 FITC (Log) | Y Parameter: CD11b PerCP (Log)
Quad Location: 6, 24

Quad	Events	% Gated	% Total	X Mean	X Geo Mean	Y Mean	Y Geo Mean
UL	23	0.30	0.26	4.68	4.53	32.70	32.05
UR	962	12.63	10.85	18.31	15.38	39.22	36.04
LL	210	2.76	2.37	4.13	3.91	11.07	9.80
LR	6420	84.31	72.42	22.95	20.59	12.18	11.39

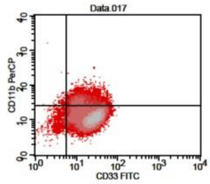

Quadrant Statistics

File: Data.017 | Log Data Units: Linear Values
Sample ID: | Patient ID:
Tube: Untitled | Panel: Untitled Acquisition Tube List
Acquisition Date: 11-Apr-24 | Gate: G1
Gated Events: 43515 | Total Events: 50000
X Parameter: CD33 FITC (Log) | Y Parameter: CD11b PerCP (Log)
Quad Location: 6, 24

Quad	Events	% Gated	% Total	X Mean	X Geo Mean	Y Mean	Y Geo Mean
UL	186	0.43	0.37	4.73	4.65	45.02	35.29
UR	5534	12.72	11.07	20.76	17.17	38.50	36.21
LL	1011	2.32	2.02	4.23	4.02	11.44	10.29
LR	36784	84.53	73.57	24.24	21.84	12.78	12.03

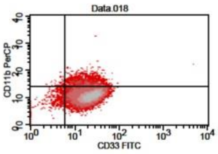

Quadrant Statistics

File: Data.018 | Log Data Units: Linear Values
Sample ID: | Patient ID:
Tube: Untitled | Panel: Untitled Acquisition Tube List
Acquisition Date: 11-Apr-24 | Gate: G1
Gated Events: 28177 | Total Events: 32490
X Parameter: CD33 FITC (Log) | Y Parameter: CD11b PerCP (Log)
Quad Location: 6, 24

Quad	Events	% Gated	% Total	X Mean	X Geo Mean	Y Mean	Y Geo Mean
UL	74	0.26	0.23	4.63	4.53	33.87	32.49
UR	2512	8.92	7.73	24.32	19.20	37.11	34.39
LL	478	1.70	1.47	4.19	3.88	10.65	9.32
LR	25113	89.13	77.29	22.34	20.37	12.36	11.61

ATRA (3)

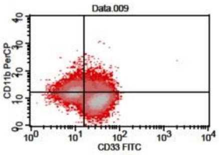

Quadrant Statistics

File: Data.009 | Log Data Units: Linear Values
Sample ID: | Patient ID:
Tube: Untitled | Panel: Untitled Acquisition Tube List
Acquisition Date: 12-Sep-24 | Gate: G1
Gated Events: 40275 | Total Events: 50000
X Parameter: CD33 FITC (Log) | Y Parameter: CD11b PerCP (Log)
Quad Location: 16, 16

Quad	Events	% Gated	% Total	X Mean	X Geo Mean	Y Mean	Y Geo Mean
UL	8453	20.99	16.91	9.72	9.25	25.95	23.65
UR	5165	12.82	10.33	34.53	31.32	31.60	26.75
LL	5824	14.46	11.65	9.14	8.62	11.82	11.38
LR	20833	51.73	41.67	34.47	32.52	8.40	7.79

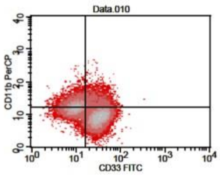

Quadrant Statistics

File: Data.010 | Log Data Units: Linear Values
Sample ID: | Patient ID:
Tube: Untitled | Panel: Untitled Acquisition Tube List
Acquisition Date: 12-Sep-24 | Gate: G1
Gated Events: 39737 | Total Events: 50000
X Parameter: CD33 FITC (Log) | Y Parameter: CD11b PerCP (Log)
Quad Location: 16, 16

Quad	Events	% Gated	% Total	X Mean	X Geo Mean	Y Mean	Y Geo Mean
UL	8337	20.98	16.67	9.84	9.35	24.68	22.94
UR	4726	11.89	9.45	33.15	30.28	30.28	26.56
LL	6709	16.88	13.42	9.16	8.64	11.78	11.36
LR	19965	50.24	39.93	34.07	32.09	8.38	7.79

Figure S4A4 (continued)

HL-60 WT
ATRA 58μM 72h

ATRA (4)

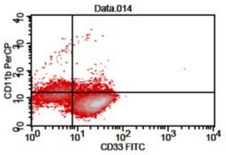

Quadrant Statistics

File: Data.014
Sample ID:
Tube: Untitled
Acquisition Date: 02-Jul-24
Gated Events: 39857
X Parameter: CD33 FITC (Log)
Quad Location: 8, 14

Log Data Units: Linear Values
Patient ID:
Panel: Untitled Acquisition Tube List
Gate: G1
Total Events: 50000
Y Parameter: CD11b PerCP (Log)

Quad	Events	% Gated	% Total	X Mean	X Geo Mean	Y Mean	Y Geo Mean
UL	2005	5.03	4.01	4.05	3.70	29.83	21.94
UR	1086	2.72	2.17	25.05	19.40	49.02	23.08
LL	2067	5.19	4.13	3.41	2.89	10.08	9.51
LR	34699	87.06	69.40	26.64	25.03	7.01	6.70

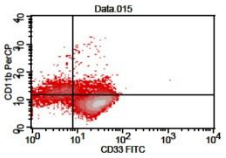

Quadrant Statistics

File: Data.015
Sample ID:
Tube: Untitled
Acquisition Date: 02-Jul-24
Gated Events: 40271
X Parameter: CD33 FITC (Log)
Quad Location: 8, 14

Log Data Units: Linear Values
Patient ID:
Panel: Untitled Acquisition Tube List
Gate: G1
Total Events: 50000
Y Parameter: CD11b PerCP (Log)

Quad	Events	% Gated	% Total	X Mean	X Geo Mean	Y Mean	Y Geo Mean
UL	2377	5.90	4.75	4.13	3.75	26.45	23.42
UR	2382	5.91	4.76	29.46	24.53	35.82	22.45
LL	1111	2.76	2.22	3.24	2.70	10.50	10.05
LR	34401	85.42	68.80	28.76	27.21	8.29	7.97

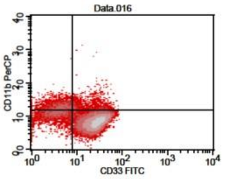

Quadrant Statistics

File: Data.016
Sample ID:
Tube: Untitled
Acquisition Date: 02-Jul-24
Gated Events: 39807
X Parameter: CD33 FITC (Log)
Quad Location: 8, 14

Log Data Units: Linear Values
Patient ID:
Panel: Untitled Acquisition Tube List
Gate: G1
Total Events: 50000
Y Parameter: CD11b PerCP (Log)

Quad	Events	% Gated	% Total	X Mean	X Geo Mean	Y Mean	Y Geo Mean
UL	2082	5.23	4.16	4.14	3.77	21.78	20.65
UR	1276	3.21	2.55	24.49	20.24	26.83	22.14
LL	2193	5.51	4.39	3.44	2.93	9.92	9.32
LR	34256	86.06	68.51	28.00	26.34	7.32	7.00

Figure S4A5

HL-60 WT

ATRA + FAs (1)

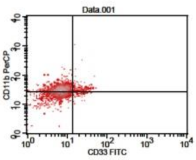

Quadrant Statistics

File: Data.001 | Log Data Units: Linear Values
Sample ID: | Patient ID:
Tube: Untitled | Panel: Untitled Acquisition Tube List
Acquisition Date: 11-Oct-24 | Gate: G1
Gated Events: 2599 | Total Events: 4740
X Parameter: CD33 FITC (Log) | Y Parameter: CD11b PerCP (Log)
Quad Location: 13, 25

Quad	Events	% Gated	% Total	X Mean	X Geo Mean	Y Mean	Y Geo Mean
UL	1647	63.37	34.75	7.10	6.63	36.41	35.25
UR	224	8.62	4.73	24.41	23.31	42.25	35.65
LL	691	26.59	14.58	5.81	5.17	19.11	18.26
LR	37	1.42	0.78	20.44	19.76	20.90	20.44

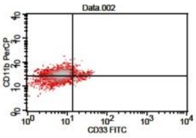

Quadrant Statistics

File: Data.002 | Log Data Units: Linear Values
Sample ID: | Patient ID:
Tube: Untitled | Panel: Untitled Acquisition Tube List
Acquisition Date: 11-Oct-24 | Gate: G1
Gated Events: 4411 | Total Events: 7740
X Parameter: CD33 FITC (Log) | Y Parameter: CD11b PerCP (Log)
Quad Location: 13, 25

Quad	Events	% Gated	% Total	X Mean	X Geo Mean	Y Mean	Y Geo Mean
UL	3077	69.76	39.75	7.18	6.74	37.85	36.68
UR	318	7.21	4.11	24.13	22.87	37.15	35.88
LL	970	21.99	12.53	5.59	4.97	19.43	18.77
LR	46	1.04	0.59	20.18	19.67	21.13	20.87

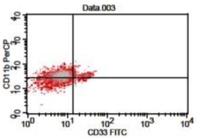

Quadrant Statistics

File: Data.003 | Log Data Units: Linear Values
Sample ID: | Patient ID:
Tube: Untitled | Panel: Untitled Acquisition Tube List
Acquisition Date: 11-Oct-24 | Gate: G1
Gated Events: 4109 | Total Events: 7260
X Parameter: CD33 FITC (Log) | Y Parameter: CD11b PerCP (Log)
Quad Location: 13, 25

Quad	Events	% Gated	% Total	X Mean	X Geo Mean	Y Mean	Y Geo Mean
UL	2875	69.97	39.60	7.15	6.68	38.40	37.26
UR	343	8.35	4.72	24.55	23.22	38.22	36.83
LL	859	20.91	11.83	5.55	4.88	19.13	18.44
LR	32	0.78	0.44	20.00	19.49	21.81	21.60

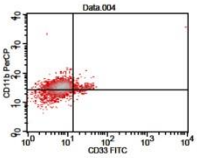

Quadrant Statistics

File: Data.004 | Log Data Units: Linear Values
Sample ID: | Patient ID:
Tube: Untitled | Panel: Untitled Acquisition Tube List
Acquisition Date: 11-Oct-24 | Gate: G1
Gated Events: 3658 | Total Events: 6645
X Parameter: CD33 FITC (Log) | Y Parameter: CD11b PerCP (Log)
Quad Location: 13, 25

Quad	Events	% Gated	% Total	X Mean	X Geo Mean	Y Mean	Y Geo Mean
UL	2570	70.26	38.68	7.16	6.71	39.24	37.18
UR	315	8.61	4.74	51.56	24.91	46.92	35.61
LL	736	20.12	11.08	5.52	4.89	19.12	18.38
LR	37	1.01	0.56	20.01	19.54	21.81	21.55

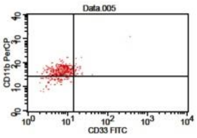

Quadrant Statistics

File: Data.005 | Log Data Units: Linear Values
Sample ID: | Patient ID:
Tube: Untitled | Panel: Untitled Acquisition Tube List
Acquisition Date: 11-Oct-24 | Gate: G1
Gated Events: 513 | Total Events: 885
X Parameter: CD33 FITC (Log) | Y Parameter: CD11b PerCP (Log)
Quad Location: 13, 25

Quad	Events	% Gated	% Total	X Mean	X Geo Mean	Y Mean	Y Geo Mean
UL	437	85.19	49.38	7.31	6.86	54.89	49.29
UR	31	6.04	3.50	29.36	19.30	93.97	57.45
LL	45	8.77	5.08	4.94	4.45	18.64	17.69
LR	0	0.00	0.00	***	***	***	***

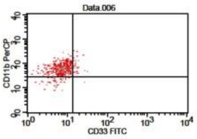

Quadrant Statistics

File: Data.006 | Log Data Units: Linear Values
Sample ID: | Patient ID:
Tube: Untitled | Panel: Untitled Acquisition Tube List
Acquisition Date: 11-Oct-24 | Gate: G1
Gated Events: 691 | Total Events: 1140
X Parameter: CD33 FITC (Log) | Y Parameter: CD11b PerCP (Log)
Quad Location: 13, 25

Quad	Events	% Gated	% Total	X Mean	X Geo Mean	Y Mean	Y Geo Mean
UL	626	90.59	54.91	7.27	6.80	65.53	60.96
UR	29	4.20	2.54	16.85	16.49	104.78	87.03
LL	35	5.07	3.07	5.18	4.77	17.96	17.19
LR	1	0.14	0.09	25.48	25.48	17.31	17.31

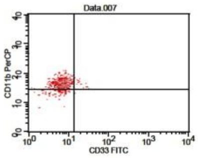

Quadrant Statistics

File: Data.007 | Log Data Units: Linear Values
Sample ID: | Patient ID:
Tube: Untitled | Panel: Untitled Acquisition Tube List
Acquisition Date: 11-Oct-24 | Gate: G1
Gated Events: 525 | Total Events: 900
X Parameter: CD33 FITC (Log) | Y Parameter: CD11b PerCP (Log)
Quad Location: 13, 25

Quad	Events	% Gated	% Total	X Mean	X Geo Mean	Y Mean	Y Geo Mean
UL	470	89.52	52.22	7.17	6.78	47.57	44.70
UR	13	2.48	1.44	20.85	20.20	48.30	46.46
LL	42	8.00	4.67	5.70	5.04	19.74	19.14
LR	0	0.00	0.00	***	***	***	***

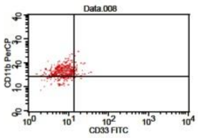

Quadrant Statistics

File: Data.008 | Log Data Units: Linear Values
Sample ID: | Patient ID:
Tube: Untitled | Panel: Untitled Acquisition Tube List
Acquisition Date: 11-Oct-24 | Gate: G1
Gated Events: 662 | Total Events: 1080
X Parameter: CD33 FITC (Log) | Y Parameter: CD11b PerCP (Log)
Quad Location: 13, 25

Quad	Events	% Gated	% Total	X Mean	X Geo Mean	Y Mean	Y Geo Mean
UL	575	86.86	53.24	7.66	7.21	52.35	48.72
UR	43	6.50	3.98	16.78	16.31	65.35	55.58
LL	43	6.50	3.98	6.22	5.65	18.89	18.24
LR	1	0.15	0.09	19.99	19.99	23.71	23.71

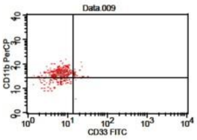

Quadrant Statistics

File: Data.009 | Log Data Units: Linear Values
Sample ID: | Patient ID:
Tube: Untitled | Panel: Untitled Acquisition Tube List
Acquisition Date: 11-Oct-24 | Gate: G1
Gated Events: 581 | Total Events: 975
X Parameter: CD33 FITC (Log) | Y Parameter: CD11b PerCP (Log)
Quad Location: 13, 25

Quad	Events	% Gated	% Total	X Mean	X Geo Mean	Y Mean	Y Geo Mean
UL	481	82.79	49.33	7.07	6.67	46.38	44.37
UR	32	5.51	3.28	17.93	17.23	42.28	39.79
LL	65	11.19	6.67	5.43	4.75	18.20	17.46
LR	3	0.52	0.31	16.01	16.01	16.87	12.79

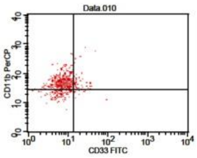

Quadrant Statistics

File: Data.010 | Log Data Units: Linear Values
Sample ID: | Patient ID:
Tube: Untitled | Panel: Untitled Acquisition Tube List
Acquisition Date: 11-Oct-24 | Gate: G1
Gated Events: 625 | Total Events: 1680
X Parameter: CD33 FITC (Log) | Y Parameter: CD11b PerCP (Log)
Quad Location: 13, 25

Quad	Events	% Gated	% Total	X Mean	X Geo Mean	Y Mean	Y Geo Mean
UL	496	79.36	29.52	7.67	7.21	57.12	51.06
UR	52	8.32	3.10	20.18	19.09	91.60	57.52
LL	70	11.20	4.17	6.02	5.56	18.63	18.03
LR	7	1.12	0.42	26.85	20.72	20.36	19.78

ATRA + FAs (2)

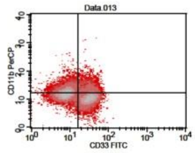

Quadrant Statistics

File: Data.013 | Log Data Units: Linear Values
Sample ID: | Patient ID:
Tube: Untitled | Panel: Untitled Acquisition Tube List
Acquisition Date: 12-Sep-24 | Gate: G1
Gated Events: 34183 | Total Events: 48735
X Parameter: CD33 FITC (Log) | Y Parameter: CD11b PerCP (Log)
Quad Location: 16, 16

Quad	Events	% Gated	% Total	X Mean	X Geo Mean	Y Mean	Y Geo Mean
UL	9877	28.89	20.27	8.50	8.00	23.94	22.25
UR	4339	12.69	8.90	32.52	30.72	28.69	24.82
LL	8189	23.96	16.80	7.72	7.22	12.20	11.91
LR	11778	34.46	24.17	32.44	30.42	10.11	9.61

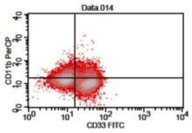

Quadrant Statistics

File: Data.014 | Log Data Units: Linear Values
Sample ID: | Patient ID:
Tube: Untitled | Panel: Untitled Acquisition Tube List
Acquisition Date: 12-Sep-24 | Gate: G1
Gated Events: 35171 | Total Events: 50000
X Parameter: CD33 FITC (Log) | Y Parameter: CD11b PerCP (Log)
Quad Location: 16, 16

Quad	Events	% Gated	% Total	X Mean	X Geo Mean	Y Mean	Y Geo Mean
UL	8991	25.56	17.98	9.52	9.09	24.09	22.36
UR	4884	13.89	9.77	36.11	33.56	29.54	25.21
LL	7587	21.57	15.17	8.69	8.25	12.25	11.98
LR	13709	38.98	27.42	36.04	34.32	9.84	9.33

Figure S4B1

HL-60 *HAX1* KO#1

Untreated 1

2024 07 02 96h

1

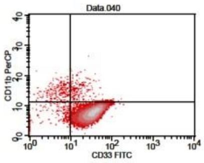

Quadrant Statistics

File: Data.040 | Log Data Units: Linear Values
Sample ID: | Patient ID:
Tube: Untitled | Panel: Untitled Acquisition Tube List
Acquisition Date: 11-Apr-24 | Gate: G1
Gated Events: 47822 | Total Events: 50000
X Parameter: CD33 FITC (Log) | Y Parameter: CD11b PerCP (Log)
Quad Location: 10, 13

Quad	Events	% Gated	% Total	X Mean	X Geo Mean	Y Mean	Y Geo Mean
UL	247	0.52	0.49	5.55	5.01	34.81	30.61
UR	342	0.72	0.68	41.67	31.68	28.90	22.32
LL	203	0.42	0.41	5.46	4.12	5.39	4.70
LR	47030	98.34	94.06	35.43	33.83	6.29	6.06

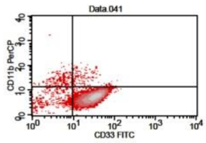

Quadrant Statistics

File: Data.041 | Log Data Units: Linear Values
Sample ID: | Patient ID:
Tube: Untitled | Panel: Untitled Acquisition Tube List
Acquisition Date: 11-Apr-24 | Gate: G1
Gated Events: 44960 | Total Events: 48135
X Parameter: CD33 FITC (Log) | Y Parameter: CD11b PerCP (Log)
Quad Location: 10, 13

Quad	Events	% Gated	% Total	X Mean	X Geo Mean	Y Mean	Y Geo Mean
UL	247	0.55	0.51	5.45	4.96	36.52	26.46
UR	260	0.58	0.54	38.77	29.91	27.92	22.59
LL	586	1.30	1.22	4.15	2.84	3.86	3.24
LR	43867	97.57	91.13	32.32	30.65	6.00	5.73

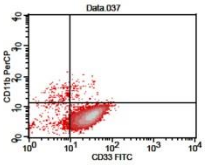

Quadrant Statistics

File: Data.037 | Log Data Units: Linear Values
Sample ID: | Patient ID:
Tube: Untitled | Panel: Untitled Acquisition Tube List
Acquisition Date: 11-Apr-24 | Gate: G1
Gated Events: 44324 | Total Events: 46830
X Parameter: CD33 FITC (Log) | Y Parameter: CD11b PerCP (Log)
Quad Location: 10, 13

Quad	Events	% Gated	% Total	X Mean	X Geo Mean	Y Mean	Y Geo Mean
UL	167	0.38	0.36	5.64	5.11	29.87	26.55
UR	109	0.25	0.23	31.23	23.74	33.89	27.43
LL	445	1.00	0.95	5.31	3.94	3.78	3.13
LR	43603	98.37	93.11	31.37	29.78	5.29	5.08

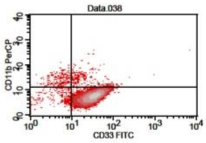

Quadrant Statistics

File: Data.038 | Log Data Units: Linear Values
Sample ID: | Patient ID:
Tube: Untitled | Panel: Untitled Acquisition Tube List
Acquisition Date: 11-Apr-24 | Gate: G1
Gated Events: 47691 | Total Events: 50000
X Parameter: CD33 FITC (Log) | Y Parameter: CD11b PerCP (Log)
Quad Location: 10, 13

Quad	Events	% Gated	% Total	X Mean	X Geo Mean	Y Mean	Y Geo Mean
UL	245	0.51	0.49	5.69	5.25	31.83	28.42
UR	473	0.99	0.95	62.85	38.79	26.11	19.85
LL	285	0.60	0.57	5.10	3.67	4.82	4.16
LR	46688	97.90	93.38	33.99	32.38	6.40	6.14

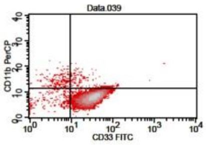

Quadrant Statistics

File: Data.039 | Log Data Units: Linear Values
Sample ID: | Patient ID:
Tube: Untitled | Panel: Untitled Acquisition Tube List
Acquisition Date: 11-Apr-24 | Gate: G1
Gated Events: 34414 | Total Events: 36495
X Parameter: CD33 FITC (Log) | Y Parameter: CD11b PerCP (Log)
Quad Location: 10, 13

Quad	Events	% Gated	% Total	X Mean	X Geo Mean	Y Mean	Y Geo Mean
UL	211	0.61	0.58	5.45	4.93	32.38	27.91
UR	398	1.16	1.09	56.86	43.18	20.50	17.63
LL	242	0.70	0.66	4.92	3.59	4.95	4.32
LR	33563	97.53	91.97	34.33	32.69	6.63	6.36

Untreated 2

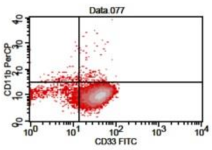

Quadrant Statistics

File: Data.077 | Log Data Units: Linear Values
Sample ID: | Patient ID:
Tube: Untitled | Panel: Untitled Acquisition Tube List
Acquisition Date: 11-Oct-24 | Gate: G1
Gated Events: 33363 | Total Events: 38625
X Parameter: CD33 FITC (Log) | Y Parameter: CD11b PerCP (Log)
Quad Location: 13, 29

Quad	Events	% Gated	% Total	X Mean	X Geo Mean	Y Mean	Y Geo Mean
UL	122	0.37	0.32	7.31	6.39	77.95	51.21
UR	221	0.66	0.57	35.07	31.14	178.29	59.85
LL	846	2.54	2.19	7.39	5.44	12.09	10.84
LR	32174	96.44	83.30	40.60	38.68	10.67	10.05

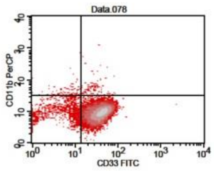

Quadrant Statistics

File: Data.078 | Log Data Units: Linear Values
Sample ID: | Patient ID:
Tube: Untitled | Panel: Untitled Acquisition Tube List
Acquisition Date: 11-Oct-24 | Gate: G1
Gated Events: 35119 | Total Events: 40905
X Parameter: CD33 FITC (Log) | Y Parameter: CD11b PerCP (Log)
Quad Location: 13, 29

Quad	Events	% Gated	% Total	X Mean	X Geo Mean	Y Mean	Y Geo Mean
UL	112	0.32	0.27	7.13	6.30	50.54	41.93
UR	115	0.33	0.28	32.75	29.03	73.22	52.00
LL	1110	3.16	2.71	6.64	4.72	9.87	8.66
LR	33782	96.19	82.59	40.00	38.11	9.50	9.04

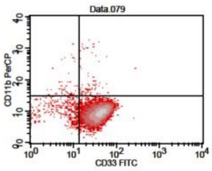

Quadrant Statistics

File: Data.079 | Log Data Units: Linear Values
Sample ID: | Patient ID:
Tube: Untitled | Panel: Untitled Acquisition Tube List
Acquisition Date: 11-Oct-24 | Gate: G1
Gated Events: 21106 | Total Events: 24315
X Parameter: CD33 FITC (Log) | Y Parameter: CD11b PerCP (Log)
Quad Location: 13, 29

Quad	Events	% Gated	% Total	X Mean	X Geo Mean	Y Mean	Y Geo Mean
UL	61	0.29	0.25	7.04	6.40	119.06	62.68
UR	99	0.47	0.41	30.68	26.24	196.39	75.46
LL	426	2.02	1.75	7.85	6.10	10.86	9.24
LR	20520	97.22	84.39	41.38	39.51	9.51	9.00

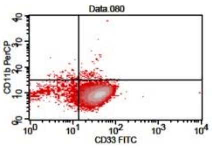

Quadrant Statistics

File: Data.080 | Log Data Units: Linear Values
Sample ID: | Patient ID:
Tube: Untitled | Panel: Untitled Acquisition Tube List
Acquisition Date: 11-Oct-24 | Gate: G1
Gated Events: 36875 | Total Events: 42600
X Parameter: CD33 FITC (Log) | Y Parameter: CD11b PerCP (Log)
Quad Location: 13, 29

Quad	Events	% Gated	% Total	X Mean	X Geo Mean	Y Mean	Y Geo Mean
UL	105	0.28	0.25	7.28	6.51	47.93	43.89
UR	185	0.50	0.43	34.07	30.46	98.91	50.35
LL	958	2.60	2.25	7.20	5.32	11.45	10.11
LR	35627	96.62	83.63	40.75	38.59	10.14	9.59

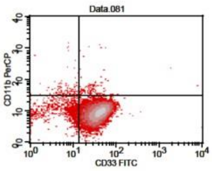

Quadrant Statistics

File: Data.081 | Log Data Units: Linear Values
Sample ID: | Patient ID:
Tube: Untitled | Panel: Untitled Acquisition Tube List
Acquisition Date: 11-Oct-24 | Gate: G1
Gated Events: 40281 | Total Events: 48705
X Parameter: CD33 FITC (Log) | Y Parameter: CD11b PerCP (Log)
Quad Location: 13, 29

Quad	Events	% Gated	% Total	X Mean	X Geo Mean	Y Mean	Y Geo Mean
UL	143	0.36	0.29	7.04	6.16	83.87	49.15
UR	168	0.42	0.34	83.79	29.27	82.68	50.10
LL	982	2.44	2.02	7.12	5.11	10.08	8.27
LR	38988	96.79	80.05	40.83	38.73	9.55	9.00

Untreated 3

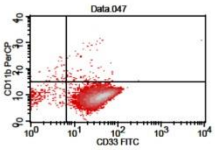

Quadrant Statistics

File: Data.047 | Log Data Units: Linear Values
Sample ID: | Patient ID:
Tube: Untitled | Panel: Untitled Acquisition Tube List
Acquisition Date: 26-Aug-24 | Gate: G1
Gated Events: 43496 | Total Events: 50000
X Parameter: CD33 FITC (Log) | Y Parameter: CD11b PerCP (Log)
Quad Location: 6, 30

Quad	Events	% Gated	% Total	X Mean	X Geo Mean	Y Mean	Y Geo Mean
UL	45	0.10	0.09	4.20	3.87	78.30	63.48
UR	109	0.25	0.22	103.43	23.64	188.47	80.84
LL	276	0.63	0.55	1.86	1.57	9.88	8.77
LR	43066	99.01	86.13	42.37	39.81	9.70	9.27

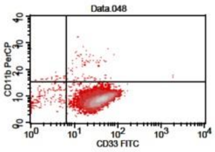

Quadrant Statistics

File: Data.048 | Log Data Units: Linear Values
Sample ID: | Patient ID:
Tube: Untitled | Panel: Untitled Acquisition Tube List
Acquisition Date: 26-Aug-24 | Gate: G1
Gated Events: 40885 | Total Events: 45855
X Parameter: CD33 FITC (Log) | Y Parameter: CD11b PerCP (Log)
Quad Location: 6, 30

Quad	Events	% Gated	% Total	X Mean	X Geo Mean	Y Mean	Y Geo Mean
UL	25	0.06	0.05	3.48	3.17	69.17	49.46
UR	85	0.21	0.19	63.05	22.06	167.29	102.89
LL	160	0.39	0.35	2.26	1.85	9.36	7.92
LR	40615	99.34	88.57	41.22	38.82	8.07	7.70

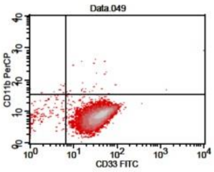

Quadrant Statistics

File: Data.049 | Log Data Units: Linear Values
Sample ID: | Patient ID:
Tube: Untitled | Panel: Untitled Acquisition Tube List
Acquisition Date: 26-Aug-24 | Gate: G1
Gated Events: 36733 | Total Events: 42690
X Parameter: CD33 FITC (Log) | Y Parameter: CD11b PerCP (Log)
Quad Location: 6, 30

Quad	Events	% Gated	% Total	X Mean	X Geo Mean	Y Mean	Y Geo Mean
UL	14	0.04	0.03	4.15	3.79	47.47	45.23
UR	39	0.11	0.09	291.73	28.44	113.81	67.41
LL	196	0.53	0.46	1.92	1.58	7.80	6.54
LR	36484	99.32	85.46	40.81	38.35	8.00	7.60

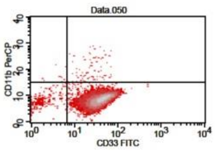

Quadrant Statistics

File: Data.050 | Log Data Units: Linear Values
Sample ID: | Patient ID:
Tube: Untitled | Panel: Untitled Acquisition Tube List
Acquisition Date: 26-Aug-24 | Gate: G1
Gated Events: 43584 | Total Events: 50000
X Parameter: CD33 FITC (Log) | Y Parameter: CD11b PerCP (Log)
Quad Location: 6, 30

Quad	Events	% Gated	% Total	X Mean	X Geo Mean	Y Mean	Y Geo Mean
UL	16	0.04	0.03	4.18	3.78	53.47	46.29
UR	81	0.19	0.16	26.71	22.43	162.55	101.52
LL	290	0.67	0.58	2.01	1.72	7.47	6.42
LR	43197	99.11	86.39	42.99	40.38	8.21	7.83

Figure S4B2

HL-60 *HAX1* KO#1
DMSO, non-induced

DMSO, non-induced (1)

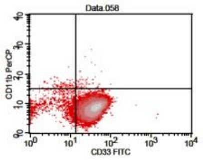

Quadrant Statistics

File: Data.058 | Log Data Units: Linear Values
Sample ID: | Patient ID:
Tube: Untitled | Panel: Untitled Acquisition Tube List
Acquisition Date: 11-Oct-24 | Gate: G1
Gated Events: 41347 | Total Events: 46470
X Parameter: CD33 FITC (Log) | Y Parameter: CD11b PerCP (Log)
Quad Location: 13, 29

Quad	Events	% Gated	% Total	X Mean	X Geo Mean	Y Mean	Y Geo Mean
UL	107	0.26	0.23	6.86	6.00	42.13	40.38
UR	66	0.16	0.14	28.87	24.99	66.96	49.77
LL	1055	2.55	2.27	6.44	4.27	8.59	7.25
LR	40119	97.03	86.33	40.40	38.37	7.65	7.19

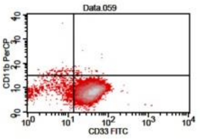

Quadrant Statistics

File: Data.059 | Log Data Units: Linear Values
Sample ID: | Patient ID:
Tube: Untitled | Panel: Untitled Acquisition Tube List
Acquisition Date: 11-Oct-24 | Gate: G1
Gated Events: 41461 | Total Events: 46425
X Parameter: CD33 FITC (Log) | Y Parameter: CD11b PerCP (Log)
Quad Location: 13, 29

Quad	Events	% Gated	% Total	X Mean	X Geo Mean	Y Mean	Y Geo Mean
UL	111	0.27	0.24	6.90	6.23	84.13	44.40
UR	54	0.13	0.12	29.85	25.70	56.36	47.46
LL	860	2.07	1.85	6.55	4.55	9.18	7.73
LR	40436	97.53	87.10	41.89	39.90	7.47	7.03

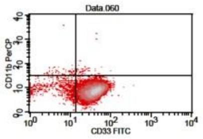

Quadrant Statistics

File: Data.060 | Log Data Units: Linear Values
Sample ID: | Patient ID:
Tube: Untitled | Panel: Untitled Acquisition Tube List
Acquisition Date: 11-Oct-24 | Gate: G1
Gated Events: 38679 | Total Events: 42900
X Parameter: CD33 FITC (Log) | Y Parameter: CD11b PerCP (Log)
Quad Location: 13, 29

Quad	Events	% Gated	% Total	X Mean	X Geo Mean	Y Mean	Y Geo Mean
UL	81	0.21	0.19	6.89	5.90	88.12	44.12
UR	72	0.19	0.17	33.43	29.16	81.93	46.83
LL	775	2.00	1.81	6.41	4.29	9.95	8.64
LR	37751	97.60	88.00	41.04	39.14	8.22	7.77

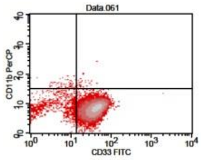

Quadrant Statistics

File: Data.061 | Log Data Units: Linear Values
Sample ID: | Patient ID:
Tube: Untitled | Panel: Untitled Acquisition Tube List
Acquisition Date: 11-Oct-24 | Gate: G1
Gated Events: 40503 | Total Events: 45375
X Parameter: CD33 FITC (Log) | Y Parameter: CD11b PerCP (Log)
Quad Location: 13, 29

Quad	Events	% Gated	% Total	X Mean	X Geo Mean	Y Mean	Y Geo Mean
UL	80	0.20	0.18	7.46	6.70	44.38	42.57
UR	42	0.10	0.09	26.17	23.28	50.36	44.80
LL	1008	2.49	2.22	5.99	3.94	8.53	7.21
LR	39373	97.21	86.77	41.23	39.21	7.30	6.88

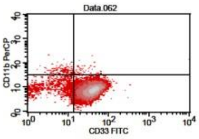

Quadrant Statistics

File: Data.062 | Log Data Units: Linear Values
Sample ID: | Patient ID:
Tube: Untitled | Panel: Untitled Acquisition Tube List
Acquisition Date: 11-Oct-24 | Gate: G1
Gated Events: 43803 | Total Events: 49080
X Parameter: CD33 FITC (Log) | Y Parameter: CD11b PerCP (Log)
Quad Location: 13, 29

Quad	Events	% Gated	% Total	X Mean	X Geo Mean	Y Mean	Y Geo Mean
UL	183	0.42	0.37	7.51	6.79	48.94	45.72
UR	93	0.21	0.19	126.33	30.54	46.63	42.64
LL	1133	2.59	2.31	6.34	4.34	10.14	8.76
LR	42394	96.78	86.38	40.67	38.67	8.62	8.10

Figure S4B3

HL-60 *HAX1* KO#1

FAs (1)

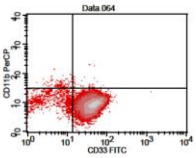

Quadrant Statistics

File: Data.064
Sample ID:
Tube: Untitled
Acquisition Date: 11-Oct-24
Gated Events: 39493
X Parameter: CD33 FITC (Log)
Quad Location: 13, 29

Log Data Units: Linear Values
Patient ID:
Panel: Untitled Acquisition Tube List
Gate: G1
Total Events: 45165
Y Parameter: CD11b PerCP (Log)

Quad	Events	% Gated	% Total	X Mean	X Geo Mean	Y Mean	Y Geo Mean
UL	136	0.34	0.30	6.73	6.05	44.20	42.20
UR	57	0.14	0.13	37.30	32.20	46.30	41.65
LL	873	2.21	1.93	5.88	4.07	12.07	10.61
LR	38427	97.30	85.08	44.29	41.99	9.12	8.58

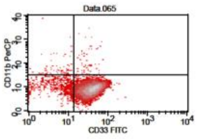

Quadrant Statistics

File: Data.065
Sample ID:
Tube: Untitled
Acquisition Date: 11-Oct-24
Gated Events: 44834
X Parameter: CD33 FITC (Log)
Quad Location: 13, 29

Log Data Units: Linear Values
Patient ID:
Panel: Untitled Acquisition Tube List
Gate: G1
Total Events: 50000
Y Parameter: CD11b PerCP (Log)

Quad	Events	% Gated	% Total	X Mean	X Geo Mean	Y Mean	Y Geo Mean
UL	103	0.23	0.21	6.46	5.57	146.47	54.36
UR	75	0.17	0.15	34.40	31.22	222.84	94.30
LL	865	1.93	1.73	7.35	5.49	10.99	9.57
LR	43791	97.67	87.58	41.79	39.81	9.05	8.61

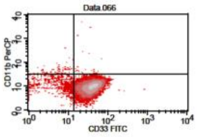

Quadrant Statistics

File: Data.066
Sample ID:
Tube: Untitled
Acquisition Date: 11-Oct-24
Gated Events: 41196
X Parameter: CD33 FITC (Log)
Quad Location: 13, 29

Log Data Units: Linear Values
Patient ID:
Panel: Untitled Acquisition Tube List
Gate: G1
Total Events: 46800
Y Parameter: CD11b PerCP (Log)

Quad	Events	% Gated	% Total	X Mean	X Geo Mean	Y Mean	Y Geo Mean
UL	109	0.26	0.23	5.46	4.58	47.49	42.42
UR	77	0.19	0.16	38.07	34.73	154.64	44.45
LL	941	2.28	2.01	6.82	4.72	11.25	9.86
LR	40069	97.26	85.62	40.27	38.27	10.47	9.84

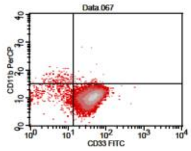

Quadrant Statistics

File: Data.067
Sample ID:
Tube: Untitled
Acquisition Date: 11-Oct-24
Gated Events: 40922
X Parameter: CD33 FITC (Log)
Quad Location: 13, 29

Log Data Units: Linear Values
Patient ID:
Panel: Untitled Acquisition Tube List
Gate: G1
Total Events: 46020
Y Parameter: CD11b PerCP (Log)

Quad	Events	% Gated	% Total	X Mean	X Geo Mean	Y Mean	Y Geo Mean
UL	175	0.43	0.38	6.32	5.57	49.88	46.83
UR	118	0.29	0.26	41.62	38.29	47.16	37.27
LL	621	1.52	1.35	7.11	5.15	12.99	11.45
LR	40008	97.77	86.94	44.24	42.11	11.11	10.52

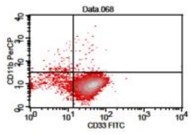

Quadrant Statistics

File: Data.068
Sample ID:
Tube: Untitled
Acquisition Date: 11-Oct-24
Gated Events: 44319
X Parameter: CD33 FITC (Log)
Quad Location: 13, 29

Log Data Units: Linear Values
Patient ID:
Panel: Untitled Acquisition Tube List
Gate: G1
Total Events: 50000
Y Parameter: CD11b PerCP (Log)

Quad	Events	% Gated	% Total	X Mean	X Geo Mean	Y Mean	Y Geo Mean
UL	154	0.35	0.31	6.55	5.75	78.80	55.07
UR	129	0.29	0.26	106.17	36.59	159.25	59.38
LL	773	1.74	1.55	7.00	5.05	11.72	10.29
LR	43263	97.62	86.53	43.42	41.35	9.85	9.34

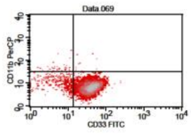

Quadrant Statistics

File: Data.069
Sample ID:
Tube: Untitled
Acquisition Date: 11-Oct-24
Gated Events: 22782
X Parameter: CD33 FITC (Log)
Quad Location: 13, 29

Log Data Units: Linear Values
Patient ID:
Panel: Untitled Acquisition Tube List
Gate: G1
Total Events: 25395
Y Parameter: CD11b PerCP (Log)

Quad	Events	% Gated	% Total	X Mean	X Geo Mean	Y Mean	Y Geo Mean
UL	61	0.27	0.24	5.99	5.09	50.08	44.12
UR	20	0.09	0.08	36.18	31.99	59.03	47.00
LL	435	1.91	1.71	7.70	6.20	11.04	9.47
LR	22266	97.74	87.68	41.90	39.99	7.52	7.09

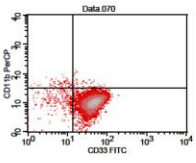

Quadrant Statistics

File: Data.070
Sample ID:
Tube: Untitled
Acquisition Date: 11-Oct-24
Gated Events: 19521
X Parameter: CD33 FITC (Log)
Quad Location: 13, 29

Log Data Units: Linear Values
Patient ID:
Panel: Untitled Acquisition Tube List
Gate: G1
Total Events: 25950
Y Parameter: CD11b PerCP (Log)

Quad	Events	% Gated	% Total	X Mean	X Geo Mean	Y Mean	Y Geo Mean
UL	80	0.41	0.31	7.07	6.16	45.03	43.27
UR	81	0.41	0.31	39.52	35.04	41.04	38.66
LL	362	1.85	1.39	6.20	4.27	9.09	5.71
LR	18998	97.32	73.21	45.97	43.71	10.44	9.80

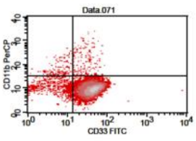

Quadrant Statistics

File: Data.071
Sample ID:
Tube: Untitled
Acquisition Date: 11-Oct-24
Gated Events: 40179
X Parameter: CD33 FITC (Log)
Quad Location: 13, 29

Log Data Units: Linear Values
Patient ID:
Panel: Untitled Acquisition Tube List
Gate: G1
Total Events: 46305
Y Parameter: CD11b PerCP (Log)

Quad	Events	% Gated	% Total	X Mean	X Geo Mean	Y Mean	Y Geo Mean
UL	228	0.57	0.49	6.64	5.88	76.35	57.98
UR	280	0.70	0.60	35.18	31.57	189.23	85.16
LL	776	1.93	1.68	6.71	4.88	11.82	10.49
LR	38895	96.80	84.00	43.93	41.66	9.99	9.46

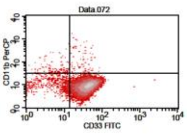

Quadrant Statistics

File: Data.072
Sample ID:
Tube: Untitled
Acquisition Date: 11-Oct-24
Gated Events: 44750
X Parameter: CD33 FITC (Log)
Quad Location: 13, 29

Log Data Units: Linear Values
Patient ID:
Panel: Untitled Acquisition Tube List
Gate: G1
Total Events: 50000
Y Parameter: CD11b PerCP (Log)

Quad	Events	% Gated	% Total	X Mean	X Geo Mean	Y Mean	Y Geo Mean
UL	155	0.35	0.31	6.44	5.46	62.51	48.86
UR	114	0.25	0.23	33.61	30.68	154.32	76.34
LL	763	1.71	1.53	7.08	5.10	10.92	9.52
LR	43718	97.69	87.44	42.60	40.52	9.75	9.22

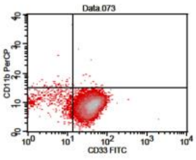

Quadrant Statistics

File: Data.073
Sample ID:
Tube: Untitled
Acquisition Date: 11-Oct-24
Gated Events: 23784
X Parameter: CD33 FITC (Log)
Quad Location: 13, 29

Log Data Units: Linear Values
Patient ID:
Panel: Untitled Acquisition Tube List
Gate: G1
Total Events: 27990
Y Parameter: CD11b PerCP (Log)

Quad	Events	% Gated	% Total	X Mean	X Geo Mean	Y Mean	Y Geo Mean
UL	68	0.29	0.24	5.59	4.65	108.31	44.75
UR	36	0.15	0.13	38.88	35.39	64.17	43.31
LL	604	2.54	2.16	5.80	3.79	10.97	9.47
LR	23076	97.02	82.44	41.74	39.66	8.44	7.80

Figure S4B4

HL-60 *HAX1* KO#1
ATRA 58μM 72h

ATRA (1)

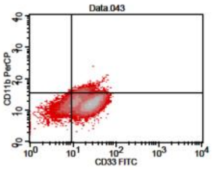

2024 07 02 96h uadrant Statistics **1**

File: Data.043 | Log Data Units: Linear Values
Sample ID: | Patient ID:
Tube: Untitled | Panel: Untitled Acquisition Tube List
Acquisition Date: 11-Apr-24 | Gate: G1
Gated Events: 42440 | Total Events: 50000
X Parameter: CD33 FITC (Log) | Y Parameter: CD11b PerCP (Log)
Quad Location: 9, 33

Quad	Events	% Gated	% Total	X Mean	X Geo Mean	Y Mean	Y Geo Mean
UL	41	0.10	0.08	8.03	7.99	36.90	36.77
UR	2478	5.84	4.96	23.35	20.71	41.73	40.91
LL	4496	10.59	8.99	6.64	6.31	14.32	13.31
LR	35425	83.47	70.85	24.41	22.22	17.87	16.85

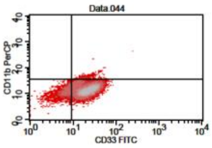

Quadrant Statistics

File: Data.044 | Log Data Units: Linear Values
Sample ID: | Patient ID:
Tube: Untitled | Panel: Untitled Acquisition Tube List
Acquisition Date: 11-Apr-24 | Gate: G1
Gated Events: 28132 | Total Events: 32745
X Parameter: CD33 FITC (Log) | Y Parameter: CD11b PerCP (Log)
Quad Location: 9, 33

Quad	Events	% Gated	% Total	X Mean	X Geo Mean	Y Mean	Y Geo Mean
UL	9	0.03	0.03	7.41	7.19	35.07	35.05
UR	559	1.99	1.71	21.75	19.59	42.56	40.96
LL	2968	10.55	9.06	6.51	6.18	13.19	12.23
LR	24596	87.43	75.11	22.53	20.89	15.43	14.55

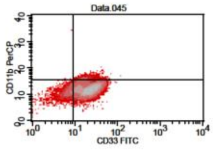

Quadrant Statistics

File: Data.045 | Log Data Units: Linear Values
Sample ID: | Patient ID:
Tube: Untitled | Panel: Untitled Acquisition Tube List
Acquisition Date: 11-Apr-24 | Gate: G1
Gated Events: 40694 | Total Events: 50000
X Parameter: CD33 FITC (Log) | Y Parameter: CD11b PerCP (Log)
Quad Location: 9, 33

Quad	Events	% Gated	% Total	X Mean	X Geo Mean	Y Mean	Y Geo Mean
UL	16	0.04	0.03	7.04	6.42	220.87	51.40
UR	1493	3.67	2.99	22.53	20.28	40.77	40.16
LL	4022	9.88	8.04	6.65	6.31	13.22	12.29
LR	35163	86.41	70.33	22.23	20.49	16.77	15.77

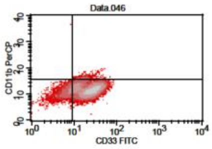

Quadrant Statistics

File: Data.046 | Log Data Units: Linear Values
Sample ID: | Patient ID:
Tube: Untitled | Panel: Untitled Acquisition Tube List
Acquisition Date: 11-Apr-24 | Gate: G1
Gated Events: 42853 | Total Events: 50000
X Parameter: CD33 FITC (Log) | Y Parameter: CD11b PerCP (Log)
Quad Location: 9, 33

Quad	Events	% Gated	% Total	X Mean	X Geo Mean	Y Mean	Y Geo Mean
UL	19	0.04	0.04	7.98	7.94	268.81	46.80
UR	1139	2.66	2.28	20.71	18.75	41.28	40.52
LL	3817	8.91	7.63	6.59	6.27	13.57	12.57
LR	37878	88.39	75.76	24.93	22.95	15.75	[illegible]

ATRA (2)

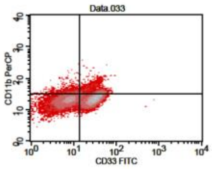

Quadrant Statistics

File: Data.033 | Log Data Units: Linear Values
Sample ID: | Patient ID:
Tube: Untitled | Panel: Untitled Acquisition Tube List
Acquisition Date: 11-Oct-24 | Gate: G1
Gated Events: 34076 | Total Events: 50000
X Parameter: CD33 FITC (Log) | Y Parameter: CD11b PerCP (Log)
Quad Location: 13, 29

Quad	Events	% Gated	% Total	X Mean	X Geo Mean	Y Mean	Y Geo Mean
UL	1760	5.16	3.52	8.34	7.73	36.78	35.68
UR	5147	15.10	10.29	36.38	34.48	38.36	36.77
LL	11432	33.55	22.86	7.01	6.41	19.19	18.40
LR	15737	46.18	31.47	28.73	27.59	20.87	20.32

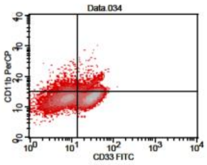

Quadrant Statistics

File: Data.034 | Log Data Units: Linear Values
Sample ID: | Patient ID:
Tube: Untitled | Panel: Untitled Acquisition Tube List
Acquisition Date: 11-Oct-24 | Gate: G1
Gated Events: 33399 | Total Events: 50000
X Parameter: CD33 FITC (Log) | Y Parameter: CD11b PerCP (Log)
Quad Location: 13, 29

Quad	Events	% Gated	% Total	X Mean	X Geo Mean	Y Mean	Y Geo Mean
UL	2218	6.64	4.44	7.99	7.44	43.80	40.42
UR	3661	10.96	7.32	34.97	32.82	41.91	38.35
LL	11950	35.78	23.90	6.96	6.31	18.17	17.35
LR	15570	46.62	31.14	28.45	27.28	19.97	19.37

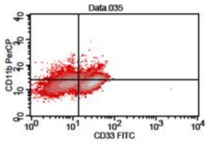

Quadrant Statistics

File: Data.035 | Log Data Units: Linear Values
Sample ID: | Patient ID:
Tube: Untitled | Panel: Untitled Acquisition Tube List
Acquisition Date: 11-Oct-24 | Gate: G1
Gated Events: 34225 | Total Events: 50000
X Parameter: CD33 FITC (Log) | Y Parameter: CD11b PerCP (Log)
Quad Location: 13, 25

Quad	Events	% Gated	% Total	X Mean	X Geo Mean	Y Mean	Y Geo Mean
UL	2942	8.60	5.88	8.16	7.62	37.24	34.66
UR	7054	20.61	14.11	34.89	33.22	34.04	32.34
LL	10037	29.33	20.07	6.93	6.31	17.14	16.48
LR	14192	41.47	28.38	27.44	26.36	18.77	18.35

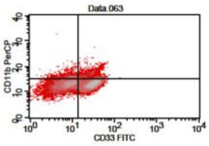

Quadrant Statistics

File: Data.063 | Log Data Units: Linear Values
Sample ID: | Patient ID:
Tube: Untitled | Panel: Untitled Acquisition Tube List
Acquisition Date: 11-Oct-24 | Gate: G1
Gated Events: 27013 | Total Events: 42870
X Parameter: CD33 FITC (Log) | Y Parameter: CD11b PerCP (Log)
Quad Location: 13, 29

Quad	Events	% Gated	% Total	X Mean	X Geo Mean	Y Mean	Y Geo Mean
UL	1869	6.92	4.36	8.37	7.81	39.58	37.07
UR	3176	11.76	7.41	33.76	31.51	40.73	38.16
LL	8642	31.99	20.16	7.37	6.70	19.17	18.34
LR	13326	49.33	31.08	28.54	27.37	20.30	19.73

ATRA (3)

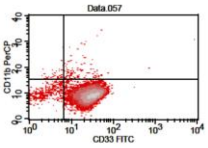

Quadrant Statistics

File: Data.057 | Log Data Units: Linear Values
Sample ID: | Patient ID:
Tube: Untitled | Panel: Untitled Acquisition Tube List
Acquisition Date: 26-Aug-24 | Gate: G1
Gated Events: 41114 | Total Events: 46965
X Parameter: CD33 FITC (Log) | Y Parameter: CD11b PerCP (Log)
Quad Location: 6, 30

Quad	Events	% Gated	% Total	X Mean	X Geo Mean	Y Mean	Y Geo Mean
UL	48	0.12	0.10	4.44	4.20	64.59	51.96
UR	154	0.37	0.33	77.64	18.52	120.81	61.77
LL	447	1.09	0.95	3.00	2.58	11.99	10.72
LR	40465	98.42	86.16	30.58	28.84	9.09	8.59

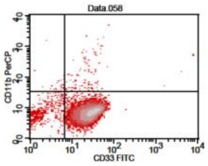

Quadrant Statistics

File: Data.058 | Log Data Units: Linear Values
Sample ID: | Patient ID:
Tube: Untitled | Panel: Untitled Acquisition Tube List
Acquisition Date: 26-Aug-24 | Gate: G1
Gated Events: 33228 | Total Events: 42030
X Parameter: CD33 FITC (Log) | Y Parameter: CD11b PerCP (Log)
Quad Location: 6, 30

Quad	Events	% Gated	% Total	X Mean	X Geo Mean	Y Mean	Y Geo Mean
UL	27	0.08	0.06	4.55	4.28	111.33	58.22
UR	112	0.34	0.27	206.52	24.38	275.92	112.83
LL	527	1.59	1.25	2.26	1.83	7.04	5.14
LR	32562	98.00	77.47	29.16	27.37	7.57	7.21

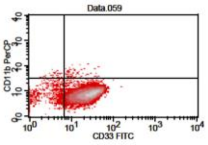

Quadrant Statistics

File: Data.059 | Log Data Units: Linear Values
Sample ID: | Patient ID:
Tube: Untitled | Panel: Untitled Acquisition Tube List
Acquisition Date: 26-Aug-24 | Gate: G1
Gated Events: 42622 | Total Events: 48885
X Parameter: CD33 FITC (Log) | Y Parameter: CD11b PerCP (Log)
Quad Location: 6, 30

Quad	Events	% Gated	% Total	X Mean	X Geo Mean	Y Mean	Y Geo Mean
UL	47	0.11	0.10	3.82	3.63	42.82	41.81
UR	75	0.18	0.15	16.75	14.06	52.71	49.62
LL	991	2.33	2.03	2.96	2.22	7.46	6.32
LR	41509	97.39	84.91	28.00	26.05	8.36	8.01

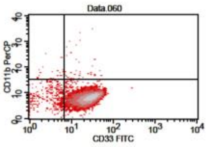

Quadrant Statistics

File: Data.060 | Log Data Units: Linear Values
Sample ID: | Patient ID:
Tube: Untitled | Panel: Untitled Acquisition Tube List
Acquisition Date: 26-Aug-24 | Gate: G1
Gated Events: 27534 | Total Events: 32520
X Parameter: CD33 FITC (Log) | Y Parameter: CD11b PerCP (Log)
Quad Location: 6, 30

Quad	Events	% Gated	% Total	X Mean	X Geo Mean	Y Mean	Y Geo Mean
UL	30	0.11	0.09	3.65	3.21	64.88	51.75
UR	48	0.17	0.15	17.18	14.02	237.10	80.42
LL	580	2.11	1.78	2.68	2.00	6.75	5.50
LR	26876	97.61	82.64	27.54	25.76	7.01	6.69

Figure S4B5

HL-60 *HAX1* KO#1

ATRA+FAs (1)

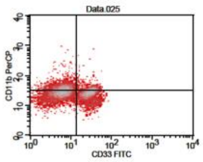

Quadrant Statistics

File: Data.025
Sample ID:
Tube: Untitled
Acquisition Date: 11-Oct-24
Gated Events: 13242
X Parameter: CD33 FITC (Log)
Quad Location: 13, 29

Log Data Units: Linear Values
Patient ID:
Panel: Untitled Acquisition Tube List
Gate: G1
Total Events: 24780
Y Parameter: CD11b PerCP (Log)

Quad	Events	% Gated	% Total	X Mean	X Geo Mean	Y Mean	Y Geo Mean
UL	4090	30.89	16.51	6.55	6.10	40.95	38.67
UR	1986	15.00	8.01	31.53	30.45	36.62	35.15
LL	4120	31.11	16.63	5.49	4.90	21.64	20.83
LR	3046	23.00	12.29	28.32	27.06	21.03	19.94

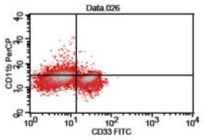

Quadrant Statistics

File: Data.026
Sample ID:
Tube: Untitled
Acquisition Date: 11-Oct-24
Gated Events: 13695
X Parameter: CD33 FITC (Log)
Quad Location: 13, 29

Log Data Units: Linear Values
Patient ID:
Panel: Untitled Acquisition Tube List
Gate: G1
Total Events: 25230
Y Parameter: CD11b PerCP (Log)

Quad	Events	% Gated	% Total	X Mean	X Geo Mean	Y Mean	Y Geo Mean
UL	4306	31.44	17.07	6.54	6.08	42.75	39.05
UR	1674	12.22	6.63	31.55	30.38	35.98	34.93
LL	4435	32.38	17.58	5.47	4.89	21.51	20.71
LR	3280	23.95	13.00	28.36	27.03	20.60	19.46

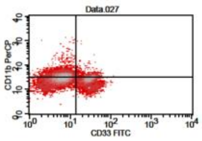

Quadrant Statistics

File: Data.027
Sample ID:
Tube: Untitled
Acquisition Date: 11-Oct-24
Gated Events: 19363
X Parameter: CD33 FITC (Log)
Quad Location: 13, 29

Log Data Units: Linear Values
Patient ID:
Panel: Untitled Acquisition Tube List
Gate: G1
Total Events: 36150
Y Parameter: CD11b PerCP (Log)

Quad	Events	% Gated	% Total	X Mean	X Geo Mean	Y Mean	Y Geo Mean
UL	7621	39.36	21.08	6.70	6.26	41.99	38.93
UR	2163	11.17	5.98	31.81	30.43	37.73	35.81
LL	6305	32.56	17.44	5.50	4.95	21.98	21.20
LR	3274	16.91	9.06	29.14	27.61	20.89	19.81

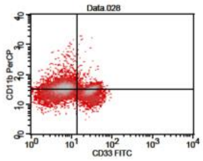

Quadrant Statistics

File: Data.028
Sample ID:
Tube: Untitled
Acquisition Date: 11-Oct-24
Gated Events: 19438
X Parameter: CD33 FITC (Log)
Quad Location: 13, 29

Log Data Units: Linear Values
Patient ID:
Panel: Untitled Acquisition Tube List
Gate: G1
Total Events: 35910
Y Parameter: CD11b PerCP (Log)

Quad	Events	% Gated	% Total	X Mean	X Geo Mean	Y Mean	Y Geo Mean
UL	9201	47.34	25.62	6.53	6.08	44.93	41.05
UR	2887	14.85	8.04	31.77	30.56	41.49	36.66
LL	4202	21.62	11.70	5.31	4.72	22.10	21.32
LR	3148	16.20	8.77	29.23	27.76	21.26	20.33

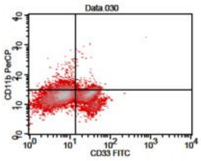

Quadrant Statistics

File: Data.030
Sample ID:
Tube: Untitled
Acquisition Date: 11-Oct-24
Gated Events: 20223
X Parameter: CD33 FITC (Log)
Quad Location: 13, 29

Log Data Units: Linear Values
Patient ID:
Panel: Untitled Acquisition Tube List
Gate: G1
Total Events: 37080
Y Parameter: CD11b PerCP (Log)

Quad	Events	% Gated	% Total	X Mean	X Geo Mean	Y Mean	Y Geo Mean
UL	1993	9.86	5.37	7.46	6.95	40.93	37.66
UR	1201	5.94	3.24	35.07	32.83	42.04	35.01
LL	11632	57.52	31.37	6.34	5.80	19.09	18.28
LR	5397	26.69	14.56	30.71	29.27	19.83	18.52

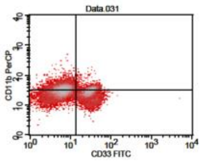

Quadrant Statistics

File: Data.031
Sample ID:
Tube: Untitled
Acquisition Date: 11-Oct-24
Gated Events: 20500
X Parameter: CD33 FITC (Log)
Quad Location: 13, 29

Log Data Units: Linear Values
Patient ID:
Panel: Untitled Acquisition Tube List
Gate: G1
Total Events: 36945
Y Parameter: CD11b PerCP (Log)

Quad	Events	% Gated	% Total	X Mean	X Geo Mean	Y Mean	Y Geo Mean
UL	7758	37.84	21.00	6.69	6.26	39.76	38.44
UR	3357	16.38	9.09	31.70	30.57	36.28	35.56
LL	5490	26.78	14.86	5.50	4.93	21.92	21.14
LR	3895	19.00	10.54	31.04	28.25	20.59	19.51

Figure S4C1

HL-60 *HAX1* KO#2

Untreated 1

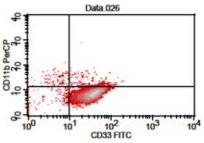

Quadrant Statistics

File: Data.026 | Log Data Units: Linear Values
Sample ID: | Patient ID:
Tube: Untitled | Panel: Untitled Acquisition Tube List
Acquisition Date: 11-Apr-24 | Gate: G1
Gated Events: 35062 | Total Events: 37275
X Parameter: CD33 FITC (Log) | Y Parameter: CD11b PerCP (Log)
Quad Location: 10, 13

Quad	Events	% Gated	% Total	X Mean	X Geo Mean	Y Mean	Y Geo Mean
UL	94	0.27	0.25	5.09	4.54	32.51	28.85
UR	1190	3.39	3.19	54.64	50.90	16.22	14.96
LL	182	0.52	0.49	4.91	3.37	4.66	3.81
LR	33596	95.82	90.13	37.02	35.39	7.67	7.43

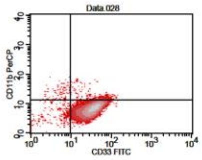

Quadrant Statistics

File: Data.028 | Log Data Units: Linear Values
Sample ID: | Patient ID:
Tube: Untitled | Panel: Untitled Acquisition Tube List
Acquisition Date: 11-Apr-24 | Gate: G1
Gated Events: 46650 | Total Events: 50000
X Parameter: CD33 FITC (Log) | Y Parameter: CD11b PerCP (Log)
Quad Location: 10, 13

Quad	Events	% Gated	% Total	X Mean	X Geo Mean	Y Mean	Y Geo Mean
UL	83	0.18	0.17	5.77	5.32	29.47	26.20
UR	826	1.77	1.65	54.34	50.31	15.43	14.77
LL	359	0.77	0.72	4.79	3.28	3.79	3.09
LR	45382	97.28	90.76	35.55	33.87	7.11	6.85

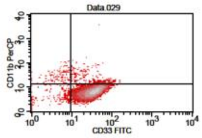

Quadrant Statistics

File: Data.029 | Log Data Units: Linear Values
Sample ID: | Patient ID:
Tube: Untitled | Panel: Untitled Acquisition Tube List
Acquisition Date: 11-Apr-24 | Gate: G1
Gated Events: 44916 | Total Events: 47865
X Parameter: CD33 FITC (Log) | Y Parameter: CD11b PerCP (Log)
Quad Location: 10, 13

Quad	Events	% Gated	% Total	X Mean	X Geo Mean	Y Mean	Y Geo Mean
UL	134	0.30	0.28	5.53	4.94	35.93	31.52
UR	994	2.21	2.08	53.22	48.53	21.13	15.75
LL	340	0.76	0.71	5.18	3.60	4.10	3.48
LR	43448	96.73	90.77	35.58	33.87	7.21	6.96

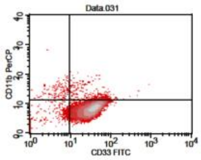

Quadrant Statistics

File: Data.031 | Log Data Units: Linear Values
Sample ID: | Patient ID:
Tube: Untitled | Panel: Untitled Acquisition Tube List
Acquisition Date: 11-Apr-24 | Gate: G1
Gated Events: 46970 | Total Events: 50000
X Parameter: CD33 FITC (Log) | Y Parameter: CD11b PerCP (Log)
Quad Location: 10, 13

Quad	Events	% Gated	% Total	X Mean	X Geo Mean	Y Mean	Y Geo Mean
UL	161	0.34	0.32	5.68	5.13	35.68	29.04
UR	926	1.97	1.85	54.01	47.45	17.32	15.80
LL	356	0.76	0.71	5.46	4.01	4.51	3.78
LR	45527	96.93	91.05	35.96	34.23	7.19	6.94

Untreated 2

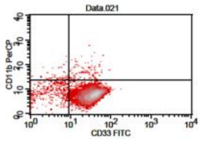

Quadrant Statistics

File: Data.021 | Log Data Units: Linear Values
Sample ID: | Patient ID:
Tube: Untitled | Panel: Untitled Acquisition Tube List
Acquisition Date: 02-Jul-24 | Gate: G1
Gated Events: 36508 | Total Events: 45165
X Parameter: CD33 FITC (Log) | Y Parameter: CD11b PerCP (Log)
Quad Location: 9, 21

Quad	Events	% Gated	% Total	X Mean	X Geo Mean	Y Mean	Y Geo Mean
UL	69	0.19	0.15	5.07	4.52	40.50	34.78
UR	128	0.35	0.28	27.66	23.68	63.27	48.73
LL	608	1.67	1.35	2.99	2.13	7.06	5.48
LR	35703	97.80	79.05	37.15	35.45	6.45	6.16

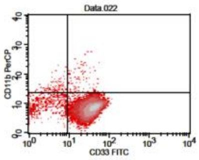

Quadrant Statistics

File: Data.022 | Log Data Units: Linear Values
Sample ID: | Patient ID:
Tube: Untitled | Panel: Untitled Acquisition Tube List
Acquisition Date: 02-Jul-24 | Gate: G1
Gated Events: 40012 | Total Events: 50000
X Parameter: CD33 FITC (Log) | Y Parameter: CD11b PerCP (Log)
Quad Location: 9, 21

Quad	Events	% Gated	% Total	X Mean	X Geo Mean	Y Mean	Y Geo Mean
UL	137	0.34	0.27	4.63	4.13	40.11	34.66
UR	140	0.35	0.28	28.58	24.40	73.05	47.38
LL	920	2.30	1.84	2.35	1.69	7.92	7.04
LR	38815	97.01	77.63	38.16	36.40	7.56	7.19

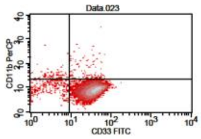

Quadrant Statistics

File: Data.023 | Log Data Units: Linear Values
Sample ID: | Patient ID:
Tube: Untitled | Panel: Untitled Acquisition Tube List
Acquisition Date: 02-Jul-24 | Gate: G1
Gated Events: 40197 | Total Events: 50000
X Parameter: CD33 FITC (Log) | Y Parameter: CD11b PerCP (Log)
Quad Location: 9, 21

Quad	Events	% Gated	% Total	X Mean	X Geo Mean	Y Mean	Y Geo Mean
UL	119	0.30	0.24	4.63	4.08	41.87	33.76
UR	183	0.46	0.37	34.21	30.13	74.68	39.22
LL	496	1.23	0.99	2.66	1.90	8.91	7.98
LR	39399	98.01	78.80	39.17	37.45	8.70	8.35

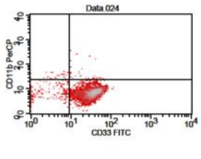

Quadrant Statistics

File: Data.024 | Log Data Units: Linear Values
Sample ID: | Patient ID:
Tube: Untitled | Panel: Untitled Acquisition Tube List
Acquisition Date: 02-Jul-24 | Gate: G1
Gated Events: 8223 | Total Events: 10530
X Parameter: CD33 FITC (Log) | Y Parameter: CD11b PerCP (Log)
Quad Location: 9, 21

Quad	Events	% Gated	% Total	X Mean	X Geo Mean	Y Mean	Y Geo Mean
UL	27	0.33	0.26	4.60	4.01	34.80	33.68
UR	49	0.60	0.47	28.87	24.59	50.47	35.26
LL	327	3.98	3.11	2.91	1.94	6.53	5.67
LR	7820	95.10	74.26	36.04	33.90	7.70	7.30

Untreated 3

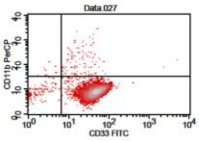

Quadrant Statistics

File: Data.027 | Log Data Units: Linear Values
Sample ID: | Patient ID:
Tube: Untitled | Panel: Untitled Acquisition Tube List
Acquisition Date: 26-Aug-24 | Gate: G1
Gated Events: 34664 | Total Events: 39180
X Parameter: CD33 FITC (Log) | Y Parameter: CD11b PerCP (Log)
Quad Location: 6, 30

Quad	Events	% Gated	% Total	X Mean	X Geo Mean	Y Mean	Y Geo Mean
UL	11	0.03	0.03	3.61	3.37	62.30	56.60
UR	119	0.34	0.30	83.62	23.47	247.97	120.61
LL	164	0.47	0.42	1.72	1.50	8.28	7.41
LR	34370	99.15	87.72	47.49	45.69	8.55	8.23

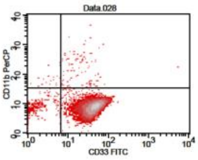

Quadrant Statistics

File: Data.028 | Log Data Units: Linear Values
Sample ID: | Patient ID:
Tube: Untitled | Panel: Untitled Acquisition Tube List
Acquisition Date: 26-Aug-24 | Gate: G1
Gated Events: 43303 | Total Events: 49230
X Parameter: CD33 FITC (Log) | Y Parameter: CD11b PerCP (Log)
Quad Location: 6, 30

Quad	Events	% Gated	% Total	X Mean	X Geo Mean	Y Mean	Y Geo Mean
UL	22	0.05	0.04	3.84	3.51	78.77	65.23
UR	104	0.24	0.21	71.47	20.47	285.85	140.69
LL	409	0.94	0.83	1.65	1.42	7.72	6.96
LR	42768	98.76	86.87	45.70	43.74	8.21	7.88

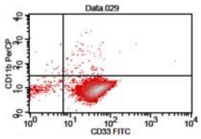

Quadrant Statistics

File: Data.029 | Log Data Units: Linear Values
Sample ID: | Patient ID:
Tube: Untitled | Panel: Untitled Acquisition Tube List
Acquisition Date: 26-Aug-24 | Gate: G1
Gated Events: 44855 | Total Events: 50000
X Parameter: CD33 FITC (Log) | Y Parameter: CD11b PerCP (Log)
Quad Location: 6, 30

Quad	Events	% Gated	% Total	X Mean	X Geo Mean	Y Mean	Y Geo Mean
UL	18	0.04	0.04	3.98	3.60	166.12	86.21
UR	110	0.25	0.22	67.30	26.69	199.39	95.12
LL	221	0.49	0.44	2.04	1.77	10.33	9.56
LR	44506	99.22	89.01	48.13	46.33	9.38	9.02

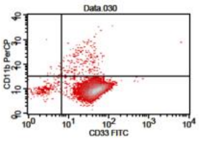

Quadrant Statistics

File: Data.030 | Log Data Units: Linear Values
Sample ID: | Patient ID:
Tube: Untitled | Panel: Untitled Acquisition Tube List
Acquisition Date: 26-Aug-24 | Gate: G1
Gated Events: 44106 | Total Events: 50000
X Parameter: CD33 FITC (Log) | Y Parameter: CD11b PerCP (Log)
Quad Location: 6, 30

Quad	Events	% Gated	% Total	X Mean	X Geo Mean	Y Mean	Y Geo Mean
UL	20	0.05	0.04	4.70	4.55	108.49	62.50
UR	223	0.51	0.45	55.91	24.72	234.30	121.37
LL	195	0.44	0.39	2.81	2.56	10.95	10.10
LR	43668	99.01	87.34	49.03	47.18	9.66	9.30

Untreated 4

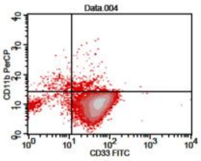

Quadrant Statistics

File: Data.004 | Log Data Units: Linear Values
Sample ID: | Patient ID:
Tube: Untitled | Panel: Untitled Acquisition Tube List
Acquisition Date: 26-Sep-24 | Gate: G1
Gated Events: 45199 | Total Events: 50000
X Parameter: CD33 FITC (Log) | Y Parameter: CD11b PerCP (Log)
Quad Location: 12, 24

Quad	Events	% Gated	% Total	X Mean	X Geo Mean	Y Mean	Y Geo Mean
UL	244	0.54	0.49	6.65	5.87	61.27	50.95
UR	762	1.69	1.52	51.52	41.11	56.33	38.33
LL	463	1.02	0.93	4.12	2.71	11.35	10.48
LR	43730	96.75	87.46	53.13	50.00	10.98	10.14

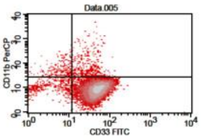

Quadrant Statistics

File: Data.005 | Log Data Units: Linear Values
Sample ID: | Patient ID:
Tube: Untitled | Panel: Untitled Acquisition Tube List
Acquisition Date: 26-Sep-24 | Gate: G1
Gated Events: 38159 | Total Events: 43005
X Parameter: CD33 FITC (Log) | Y Parameter: CD11b PerCP (Log)
Quad Location: 12, 24

Quad	Events	% Gated	% Total	X Mean	X Geo Mean	Y Mean	Y Geo Mean
UL	260	0.68	0.60	7.00	6.25	75.42	58.56
UR	592	1.55	1.38	41.18	34.85	124.17	56.28
LL	464	1.22	1.08	4.74	3.28	11.53	10.52
LR	36843	96.55	85.67	51.70	48.68	9.78	8.99

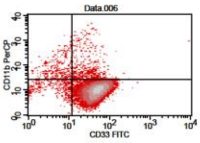

Quadrant Statistics

File: Data.006 | Log Data Units: Linear Values
Sample ID: | Patient ID:
Tube: Untitled | Panel: Untitled Acquisition Tube List
Acquisition Date: 26-Sep-24 | Gate: G1
Gated Events: 45610 | Total Events: 50000
X Parameter: CD33 FITC (Log) | Y Parameter: CD11b PerCP (Log)
Quad Location: 12, 24

Quad	Events	% Gated	% Total	X Mean	X Geo Mean	Y Mean	Y Geo Mean
UL	370	0.81	0.74	6.20	5.36	130.85	75.54
UR	841	1.84	1.68	55.11	37.28	167.64	56.68
LL	421	0.92	0.84	5.43	3.67	11.85	10.42
LR	43978	96.42	87.96	50.97	48.20	10.86	10.10

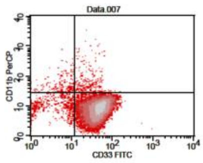

Quadrant Statistics

File: Data.007 | Log Data Units: Linear Values
Sample ID: | Patient ID:
Tube: Untitled | Panel: Untitled Acquisition Tube List
Acquisition Date: 26-Sep-24 | Gate: G1
Gated Events: 44628 | Total Events: 50000
X Parameter: CD33 FITC (Log) | Y Parameter: CD11b PerCP (Log)
Quad Location: 12, 24

Quad	Events	% Gated	% Total	X Mean	X Geo Mean	Y Mean	Y Geo Mean
UL	214	0.48	0.43	6.49	5.60	62.87	48.62
UR	373	0.84	0.75	34.27	29.54	110.81	56.59
LL	820	1.84	1.64	4.14	2.56	9.48	8.50
LR	43221	96.85	86.44	48.70	45.92	8.92	8.11

Figure S4C2

HL-60 *HAX1* KO#2
DMSO, non-induced

DMSO, non-induced (1)

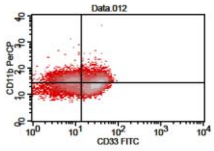

Quadrant Statistics

File: Data.012
Sample ID:
Tube: Untitled
Acquisition Date: 11-Oct-24
Gated Events: 38861
X Parameter: CD33 FITC (Log)
Quad Location: 13, 25

Log Data Units: Linear Values
Patient ID:
Panel: Untitled Acquisition Tube List
Gate: G1
Total Events: 50000
Y Parameter: CD11b PerCP (Log)

Quad	Events	% Gated	% Total	X Mean	X Geo Mean	Y Mean	Y Geo Mean
UL	7619	19.61	15.24	7.91	7.37	39.16	37.20
UR	14245	36.66	28.49	33.67	31.92	36.31	34.49
LL	3484	8.97	6.97	7.51	6.53	18.29	17.65
LR	13513	34.77	27.03	26.41	25.36	19.22	18.81

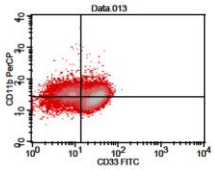

Quadrant Statistics

File: Data.013
Sample ID:
Tube: Untitled
Acquisition Date: 11-Oct-24
Gated Events: 39607
X Parameter: CD33 FITC (Log)
Quad Location: 13, 25

Log Data Units: Linear Values
Patient ID:
Panel: Untitled Acquisition Tube List
Gate: G1
Total Events: 50000
Y Parameter: CD11b PerCP (Log)

Quad	Events	% Gated	% Total	X Mean	X Geo Mean	Y Mean	Y Geo Mean
UL	7858	19.84	15.72	7.81	7.28	41.10	38.07
UR	15115	38.16	30.23	33.35	31.76	36.53	34.84
LL	2806	7.08	5.61	7.15	6.15	18.65	18.00
LR	13828	34.91	27.66	26.50	25.49	19.31	18.91

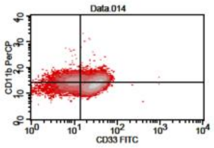

Quadrant Statistics

File: Data.014
Sample ID:
Tube: Untitled
Acquisition Date: 11-Oct-24
Gated Events: 40802
X Parameter: CD33 FITC (Log)
Quad Location: 13, 25

Log Data Units: Linear Values
Patient ID:
Panel: Untitled Acquisition Tube List
Gate: G1
Total Events: 50000
Y Parameter: CD11b PerCP (Log)

Quad	Events	% Gated	% Total	X Mean	X Geo Mean	Y Mean	Y Geo Mean
UL	6281	15.39	12.56	8.41	7.88	37.84	36.13
UR	14721	36.08	29.44	34.26	32.36	35.48	33.98
LL	3666	8.98	7.33	7.74	6.84	18.45	17.84
LR	16134	39.54	32.27	27.54	26.36	19.02	18.58

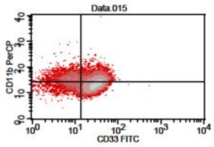

Quadrant Statistics

File: Data.015
Sample ID:
Tube: Untitled
Acquisition Date: 11-Oct-24
Gated Events: 42012
X Parameter: CD33 FITC (Log)
Quad Location: 13, 25

Log Data Units: Linear Values
Patient ID:
Panel: Untitled Acquisition Tube List
Gate: G1
Total Events: 50000
Y Parameter: CD11b PerCP (Log)

Quad	Events	% Gated	% Total	X Mean	X Geo Mean	Y Mean	Y Geo Mean
UL	4529	10.78	9.06	7.79	7.18	43.97	40.70
UR	15580	37.08	31.16	33.65	31.97	37.20	34.90
LL	2051	4.88	4.10	8.49	7.51	17.80	17.14
LR	19852	47.25	39.70	27.21	26.09	18.55	18.09

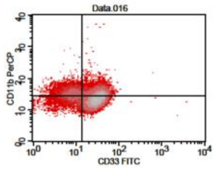

File: Data.016
Sample ID:
Tube: Untitled
Acquisition Date: 11-Oct-24
Gated Events: 41305
X Parameter: CD33 FITC (Log)
Quad Location: 13, 25

Log Data Units: Linear Values
Patient ID:
Panel: Untitled Acquisition Tube List
Gate: G1
Total Events: 50000
Y Parameter: CD11b PerCP (Log)

Quad	Events	% Gated	% Total	X Mean	X Geo Mean	Y Mean	Y Geo Mean
UL	4733	11.46	9.47	7.89	7.31	40.32	38.03
UR	15108	36.58	30.22	34.62	32.92	37.79	34.77
LL	2178	5.27	4.36	7.76	6.74	18.40	17.73
LR	19286	46.69	38.57	28.58	27.05	18.29	17.80

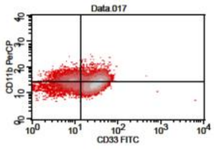

Quadrant Statistics

File: Data.017
Sample ID:
Tube: Untitled
Acquisition Date: 11-Oct-24
Gated Events: 41893
X Parameter: CD33 FITC (Log)
Quad Location: 13, 25

Log Data Units: Linear Values
Patient ID:
Panel: Untitled Acquisition Tube List
Gate: G1
Total Events: 50000
Y Parameter: CD11b PerCP (Log)

Quad	Events	% Gated	% Total	X Mean	X Geo Mean	Y Mean	Y Geo Mean
UL	4918	11.74	9.84	8.03	7.50	39.28	37.58
UR	17400	41.53	34.80	34.47	32.84	35.75	34.38
LL	2732	6.52	5.46	7.64	6.58	18.09	17.45
LR	16843	40.20	33.69	27.28	25.92	19.22	18.81

Figure S4C3

HL-60 *HAX1* KO#2

FAs (1)

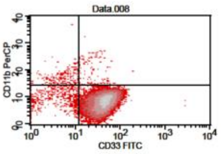

Quadrant Statistics

File: Data.008
Sample ID:
Tube: Untitled
Acquisition Date: 26-Sep-24
Gated Events: 44654
X Parameter: CD33 FITC (Log)
Quad Location: 12, 24

Log Data Units: Linear Values
Patient ID:
Panel: Untitled Acquisition Tube List
Gate: G1
Total Events: 50000
Y Parameter: CD11b PerCP (Log)

Quad	Events	% Gated	% Total	X Mean	X Geo Mean	Y Mean	Y Geo Mean
UL	231	0.52	0.46	5.87	5.04	82.84	54.34
UR	180	0.40	0.36	37.51	31.79	109.45	58.02
LL	751	1.68	1.50	4.28	2.67	8.16	6.83
LR	43492	97.40	86.98	45.12	42.34	7.74	6.92

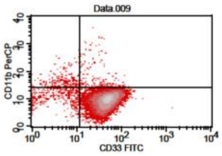

Quadrant Statistics

File: Data.009
Sample ID:
Tube: Untitled
Acquisition Date: 26-Sep-24
Gated Events: 45123
X Parameter: CD33 FITC (Log)
Quad Location: 12, 24

Log Data Units: Linear Values
Patient ID:
Panel: Untitled Acquisition Tube List
Gate: G1
Total Events: 49680
Y Parameter: CD11b PerCP (Log)

Quad	Events	% Gated	% Total	X Mean	X Geo Mean	Y Mean	Y Geo Mean
UL	242	0.54	0.49	5.71	4.96	59.94	47.08
UR	280	0.62	0.56	45.99	38.22	121.82	46.79
LL	474	1.05	0.95	5.00	3.37	11.78	10.30
LR	44127	97.79	88.82	48.99	46.45	10.09	9.34

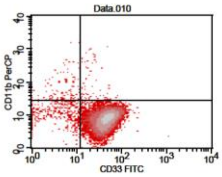

Quadrant Statistics

File: Data.010
Sample ID:
Tube: Untitled
Acquisition Date: 26-Sep-24
Gated Events: 38443
X Parameter: CD33 FITC (Log)
Quad Location: 12, 24

Log Data Units: Linear Values
Patient ID:
Panel: Untitled Acquisition Tube List
Gate: G1
Total Events: 42525
Y Parameter: CD11b PerCP (Log)

Quad	Events	% Gated	% Total	X Mean	X Geo Mean	Y Mean	Y Geo Mean
UL	137	0.36	0.32	5.69	4.90	94.54	53.09
UR	122	0.32	0.29	32.58	28.04	128.58	53.90
LL	412	1.07	0.97	5.03	3.43	10.47	9.23
LR	37772	98.25	88.82	46.88	44.45	8.16	7.58

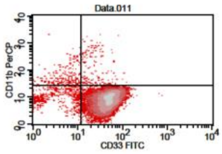

Quadrant Statistics

File: Data.011
Sample ID:
Tube: Untitled
Acquisition Date: 26-Sep-24
Gated Events: 44692
X Parameter: CD33 FITC (Log)
Quad Location: 12, 24

Log Data Units: Linear Values
Patient ID:
Panel: Untitled Acquisition Tube List
Gate: G1
Total Events: 50000
Y Parameter: CD11b PerCP (Log)

Quad	Events	% Gated	% Total	X Mean	X Geo Mean	Y Mean	Y Geo Mean
UL	267	0.60	0.53	5.69	4.87	80.71	53.49
UR	252	0.56	0.50	42.75	34.67	185.75	70.77
LL	570	1.28	1.14	4.79	3.20	10.18	8.77
LR	43603	97.56	87.21	48.49	45.86	9.15	8.16

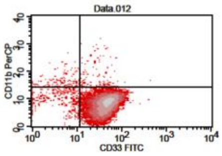

Quadrant Statistics

File: Data.012
Sample ID:
Tube: Untitled
Acquisition Date: 26-Sep-24
Gated Events: 40533
X Parameter: CD33 FITC (Log)
Quad Location: 12, 24

Log Data Units: Linear Values
Patient ID:
Panel: Untitled Acquisition Tube List
Gate: G1
Total Events: 45045
Y Parameter: CD11b PerCP (Log)

Quad	Events	% Gated	% Total	X Mean	X Geo Mean	Y Mean	Y Geo Mean
UL	147	0.36	0.33	5.36	4.45	55.53	45.23
UR	163	0.40	0.36	39.99	33.61	110.32	53.62
LL	420	1.04	0.93	5.43	3.66	9.29	7.61
LR	39803	98.20	88.36	46.82	44.32	7.75	6.98

Figure S4C4

HL-60 *HAX1* KO#2
ATRA 58μM 72h

ATRA (1)

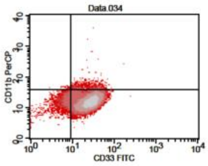

Quadrant Statistics

File: Data.034 | Log Data Units: Linear Values
Sample ID: | Patient ID:
Tube: Untitled | Panel: Untitled Acquisition Tube List
Acquisition Date: 11-Apr-24 | Gate: G1
Gated Events: 44719 | Total Events: 50000
X Parameter: CD33 FITC (Log) | Y Parameter: CD11b PerCP (Log)
Quad Location: 9, 36

Quad	Events	% Gated	% Total	X Mean	X Geo Mean	Y Mean	Y Geo Mean
UL	88	0.20	0.18	7.48	7.36	42.08	41.66
UR	1494	3.34	2.99	29.65	25.69	49.27	44.24
LL	2084	4.66	4.17	6.58	6.22	17.13	15.67
LR	41053	91.80	82.11	26.89	25.03	16.90	15.84

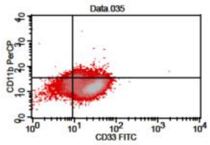

Quadrant Statistics

File: Data.035 | Log Data Units: Linear Values
Sample ID: | Patient ID:
Tube: Untitled | Panel: Untitled Acquisition Tube List
Acquisition Date: 11-Apr-24 | Gate: G1
Gated Events: 44297 | Total Events: 50000
X Parameter: CD33 FITC (Log) | Y Parameter: CD11b PerCP (Log)
Quad Location: 9, 33

Quad	Events	% Gated	% Total	X Mean	X Geo Mean	Y Mean	Y Geo Mean
UL	622	1.40	1.24	7.31	7.15	47.00	41.32
UR	3397	7.67	6.79	25.68	20.92	45.79	43.49
LL	3507	7.92	7.01	6.47	6.11	18.16	16.72
LR	36771	83.01	73.54	28.39	26.19	16.93	15.85

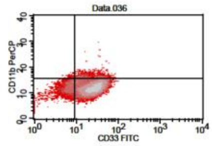

Quadrant Statistics

File: Data.036 | Log Data Units: Linear Values
Sample ID: | Patient ID:
Tube: Untitled | Panel: Untitled Acquisition Tube List
Acquisition Date: 11-Apr-24 | Gate: G1
Gated Events: 43673 | Total Events: 50000
X Parameter: CD33 FITC (Log) | Y Parameter: CD11b PerCP (Log)
Quad Location: 9, 33

Quad	Events	% Gated	% Total	X Mean	X Geo Mean	Y Mean	Y Geo Mean
UL	155	0.35	0.31	7.51	7.42	39.70	39.25
UR	2218	5.08	4.44	27.81	23.98	42.49	40.93
LL	2827	6.47	5.65	6.51	6.14	16.88	15.46
LR	38473	88.09	76.95	26.28	24.39	16.71	15.70

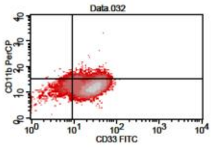

Quadrant Statistics

File: Data.032 | Log Data Units: Linear Values
Sample ID: | Patient ID:
Tube: Untitled | Panel: Untitled Acquisition Tube List
Acquisition Date: 11-Apr-24 | Gate: G1
Gated Events: 43855 | Total Events: 50000
X Parameter: CD33 FITC (Log) | Y Parameter: CD11b PerCP (Log)
Quad Location: 9, 33

Quad	Events	% Gated	% Total	X Mean	X Geo Mean	Y Mean	Y Geo Mean
UL	280	0.64	0.56	7.40	7.27	41.66	40.88
UR	2909	6.63	5.82	32.06	27.78	43.76	41.85
LL	1972	4.50	3.94	6.32	5.87	18.11	16.49
LR	38694	88.23	77.39	28.97	27.09	17.58	16.59

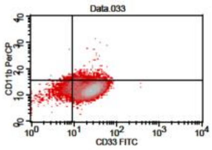

Quadrant Statistics

File: Data.033 | Log Data Units: Linear Values
Sample ID: | Patient ID:
Tube: Untitled | Panel: Untitled Acquisition Tube List
Acquisition Date: 11-Apr-24 | Gate: G1
Gated Events: 33569 | Total Events: 37725
X Parameter: CD33 FITC (Log) | Y Parameter: CD11b PerCP (Log)
Quad Location: 9, 33

Quad	Events	% Gated	% Total	X Mean	X Geo Mean	Y Mean	Y Geo Mean
UL	202	0.60	0.54	7.29	7.15	43.74	41.23
UR	1813	5.40	4.81	28.11	24.08	45.70	42.14
LL	1897	5.65	5.03	6.54	6.14	17.81	16.35
LR	29657	88.35	78.61	26.23	24.42	17.09	16.12

ATRA (2)

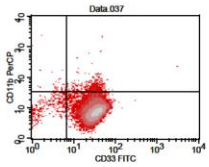

Quadrant Statistics

File: Data.037 | Log Data Units: Linear Values
Sample ID: | Patient ID:
Tube: Untitled | Panel: Untitled Acquisition Tube List
Acquisition Date: 26-Aug-24 | Gate: G1
Gated Events: 31403 | Total Events: 36525
X Parameter: CD33 FITC (Log) | Y Parameter: CD11b PerCP (Log)
Quad Location: 6, 30

Quad	Events	% Gated	% Total	X Mean	X Geo Mean	Y Mean	Y Geo Mean
UL	55	0.18	0.15	4.44	4.14	110.08	52.82
UR	624	1.99	1.71	34.62	27.69	53.51	45.22
LL	350	1.11	0.96	2.97	2.33	11.95	10.07
LR	30374	96.72	83.16	33.96	32.33	8.53	7.90

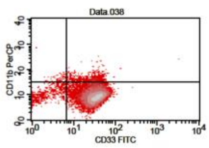

Quadrant Statistics

File: Data.038 | Log Data Units: Linear Values
Sample ID: | Patient ID:
Tube: Untitled | Panel: Untitled Acquisition Tube List
Acquisition Date: 26-Aug-24 | Gate: G1
Gated Events: 39516 | Total Events: 44925
X Parameter: CD33 FITC (Log) | Y Parameter: CD11b PerCP (Log)
Quad Location: 6, 30

Quad	Events	% Gated	% Total	X Mean	X Geo Mean	Y Mean	Y Geo Mean
UL	81	0.20	0.18	4.54	4.28	51.24	42.65
UR	806	2.04	1.79	32.30	25.28	46.31	43.23
LL	519	1.31	1.16	2.64	2.17	12.23	10.76
LR	38110	96.44	84.83	34.48	32.79	9.35	8.71

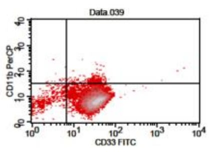

Quadrant Statistics

File: Data.039 | Log Data Units: Linear Values
Sample ID: | Patient ID:
Tube: Untitled | Panel: Untitled Acquisition Tube List
Acquisition Date: 26-Aug-24 | Gate: G1
Gated Events: 33210 | Total Events: 38175
X Parameter: CD33 FITC (Log) | Y Parameter: CD11b PerCP (Log)
Quad Location: 6, 30

Quad	Events	% Gated	% Total	X Mean	X Geo Mean	Y Mean	Y Geo Mean
UL	26	0.08	0.07	4.81	4.63	54.53	45.35
UR	402	1.21	1.05	50.52	28.48	45.39	42.60
LL	516	1.55	1.35	2.74	2.27	9.00	7.41
LR	32266	97.16	84.52	34.91	33.15	7.66	7.09

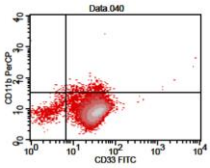

Quadrant Statistics

File: Data.040 | Log Data Units: Linear Values
Sample ID: | Patient ID:
Tube: Untitled | Panel: Untitled Acquisition Tube List
Acquisition Date: 26-Aug-24 | Gate: G1
Gated Events: 38305 | Total Events: 44055
X Parameter: CD33 FITC (Log) | Y Parameter: CD11b PerCP (Log)
Quad Location: 6, 30

Quad	Events	% Gated	% Total	X Mean	X Geo Mean	Y Mean	Y Geo Mean
UL	25	0.07	0.06	4.77	4.53	103.27	48.33
UR	557	1.45	1.26	63.55	27.88	49.30	42.81
LL	574	1.50	1.30	3.04	2.77	9.64	8.73
LR	37149	96.98	84.32	35.60	33.83	8.99	8.38

ATRA (3)

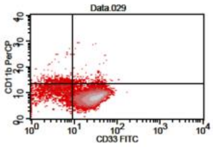

Quadrant Statistics

File: Data.029 | Log Data Units: Linear Values
Sample ID: | Patient ID:
Tube: Untitled | Panel: Untitled Acquisition Tube List
Acquisition Date: 02-Jul-24 | Gate: G1
Gated Events: 30960 | Total Events: 40560
X Parameter: CD33 FITC (Log) | Y Parameter: CD11b PerCP (Log)
Quad Location: 9, 21

Quad	Events	% Gated	% Total	X Mean	X Geo Mean	Y Mean	Y Geo Mean
UL	559	1.81	1.38	4.50	4.05	34.84	30.05
UR	248	0.80	0.61	24.75	20.80	50.04	33.21
LL	1906	6.16	4.70	4.40	3.61	11.41	10.05
LR	28247	91.24	69.64	29.75	27.93	7.30	6.96

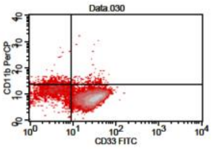

Quadrant Statistics

File: Data.030 | Log Data Units: Linear Values
Sample ID: | Patient ID:
Tube: Untitled | Panel: Untitled Acquisition Tube List
Acquisition Date: 02-Jul-24 | Gate: G1
Gated Events: 30171 | Total Events: 38865
X Parameter: CD33 FITC (Log) | Y Parameter: CD11b PerCP (Log)
Quad Location: 9, 21

Quad	Events	% Gated	% Total	X Mean	X Geo Mean	Y Mean	Y Geo Mean
UL	626	2.07	1.61	4.19	3.74	32.83	29.01
UR	235	0.78	0.60	26.99	23.37	52.38	32.52
LL	1693	5.61	4.36	4.06	3.40	12.49	11.36
LR	27617	91.53	71.06	31.57	29.80	7.77	7.40

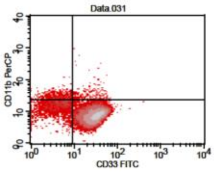

Quadrant Statistics

File: Data.031 | Log Data Units: Linear Values
Sample ID: | Patient ID:
Tube: Untitled | Panel: Untitled Acquisition Tube List
Acquisition Date: 02-Jul-24 | Gate: G1
Gated Events: 37937 | Total Events: 50000
X Parameter: CD33 FITC (Log) | Y Parameter: CD11b PerCP (Log)
Quad Location: 9, 21

Quad	Events	% Gated	% Total	X Mean	X Geo Mean	Y Mean	Y Geo Mean
UL	899	2.37	1.80	4.64	4.17	29.29	28.44
UR	384	1.01	0.77	25.21	21.39	39.11	31.45
LL	1981	5.22	3.96	4.25	3.54	12.87	11.74
LR	34673	91.40	69.35	31.59	29.86	8.13	7.75

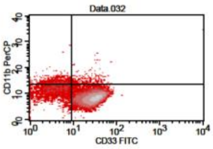

Quadrant Statistics

File: Data.032 | Log Data Units: Linear Values
Sample ID: | Patient ID:
Tube: Untitled | Panel: Untitled Acquisition Tube List
Acquisition Date: 02-Jul-24 | Gate: G1
Gated Events: 37238 | Total Events: 50000
X Parameter: CD33 FITC (Log) | Y Parameter: CD11b PerCP (Log)
Quad Location: 9, 21

Quad	Events	% Gated	% Total	X Mean	X Geo Mean	Y Mean	Y Geo Mean
UL	952	2.56	1.90	4.51	4.07	31.38	29.43
UR	321	0.86	0.64	51.51	20.12	46.81	33.05
LL	2239	6.01	4.48	4.18	3.45	12.31	11.10
LR	33726	90.57	67.45	30.64	28.85	7.77	7.41

ATRA (4)

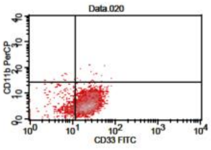

Quadrant Statistics

File: Data.020 | Log Data Units: Linear Values
Sample ID: | Patient ID:
Tube: Untitled | Panel: Untitled Acquisition Tube List
Acquisition Date: 26-Sep-24 | Gate: G1
Gated Events: 3854 | Total Events: 5040
X Parameter: CD33 FITC (Log) | Y Parameter: CD11b PerCP (Log)
Quad Location: 12, 24

Quad	Events	% Gated	% Total	X Mean	X Geo Mean	Y Mean	Y Geo Mean
UL	13	0.34	0.26	6.06	5.37	37.47	35.87
UR	20	0.52	0.40	28.86	25.21	49.75	42.53
LL	550	14.27	10.91	7.61	6.25	3.88	2.93
LR	3271	84.87	64.90	24.50	23.10	5.04	4.27

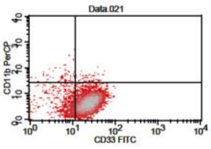

Quadrant Statistics

File: Data.021 | Log Data Units: Linear Values
Sample ID: | Patient ID:
Tube: Untitled | Panel: Untitled Acquisition Tube List
Acquisition Date: 26-Sep-24 | Gate: G1
Gated Events: 11873 | Total Events: 15480
X Parameter: CD33 FITC (Log) | Y Parameter: CD11b PerCP (Log)
Quad Location: 12, 24

Quad	Events	% Gated	% Total	X Mean	X Geo Mean	Y Mean	Y Geo Mean
UL	80	0.67	0.52	6.43	5.59	59.99	46.64
UR	69	0.58	0.45	27.74	25.49	38.94	36.45
LL	1176	9.90	7.60	7.20	5.42	4.14	3.10
LR	10548	88.84	68.14	25.54	24.05	5.25	4.63

Figure S4C4 (countinued)

HL-60 *HAX1* KO#2
ATRA 58μM 72h

ATRA (4)

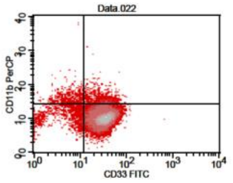

Quadrant Statistics

File: Data.022
Sample ID:
Tube: Untitled
Acquisition Date: 26-Sep-24
Gated Events: 25897
X Parameter: CD33 FITC (Log)
Quad Location: 12, 24

Log Data Units: Linear Values
Patient ID:
Panel: Untitled Acquisition Tube List
Gate: G1
Total Events: 32715
Y Parameter: CD11b PerCP (Log)

Quad	Events	% Gated	% Total	X Mean	X Geo Mean	Y Mean	Y Geo Mean
UL	572	2.21	1.75	6.53	5.89	65.14	41.74
UR	1187	4.58	3.63	33.12	30.31	38.18	34.24
LL	1287	4.97	3.93	5.40	3.59	11.49	10.30
LR	22851	88.24	69.85	32.86	30.96	10.95	10.25

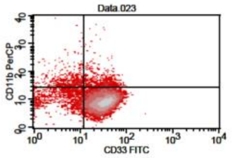

Quadrant Statistics

File: Data.023
Sample ID:
Tube: Untitled
Acquisition Date: 26-Sep-24
Gated Events: 35329
X Parameter: CD33 FITC (Log)
Quad Location: 12, 24

Log Data Units: Linear Values
Patient ID:
Panel: Untitled Acquisition Tube List
Gate: G1
Total Events: 44115
Y Parameter: CD11b PerCP (Log)

Quad	Events	% Gated	% Total	X Mean	X Geo Mean	Y Mean	Y Geo Mean
UL	696	1.97	1.58	6.35	5.66	81.20	46.31
UR	932	2.64	2.11	32.50	29.27	68.93	40.58
LL	1577	4.46	3.57	5.38	3.65	10.65	9.29
LR	32124	90.93	72.82	33.62	31.74	9.10	8.51

ATRA (5)

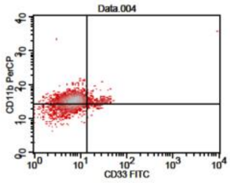

Quadrant Statistics

File: Data.004
Sample ID:
Tube: Untitled
Acquisition Date: 11-Oct-24
Gated Events: 3658
X Parameter: CD33 FITC (Log)
Quad Location: 13, 25

Log Data Units: Linear Values
Patient ID:
Panel: Untitled Acquisition Tube List
Gate: G1
Total Events: 6645
Y Parameter: CD11b PerCP (Log)

Quad	Events	% Gated	% Total	X Mean	X Geo Mean	Y Mean	Y Geo Mean
UL	2570	70.26	38.68	7.16	6.71	39.24	37.18
UR	315	8.61	4.74	51.56	24.91	46.92	35.61
LL	736	20.12	11.08	5.52	4.89	19.12	18.38
LR	37	1.01	0.56	20.01	19.54	21.81	21.55

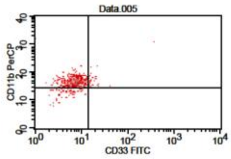

Quadrant Statistics

File: Data.005
Sample ID:
Tube: Untitled
Acquisition Date: 11-Oct-24
Gated Events: 513
X Parameter: CD33 FITC (Log)
Quad Location: 13, 25

Log Data Units: Linear Values
Patient ID:
Panel: Untitled Acquisition Tube List
Gate: G1
Total Events: 885
Y Parameter: CD11b PerCP (Log)

Quad	Events	% Gated	% Total	X Mean	X Geo Mean	Y Mean	Y Geo Mean
UL	437	85.19	49.38	7.31	6.86	54.89	49.29
UR	31	6.04	3.50	29.36	19.30	93.97	57.45
LL	45	8.77	5.08	4.94	4.45	18.64	17.69
LR	0	0.00	0.00	***	***	***	***

Figure S4C5

HL-60 *HAX1* KO#2

ATRA+FAs (1)

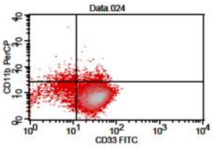

Quadrant Statistics

File: Data.024
Sample ID:
Tube: Untitled
Acquisition Date: 26-Sep-24
Gated Events: 28622
X Parameter: CD33 FITC (Log)
Quad Location: 12, 24
Log Data Units: Linear Values
Patient ID:
Panel: Untitled Acquisition Tube List
Gate: G1
Total Events: 36495
Y Parameter: CD11b PerCP (Log)

Quad	Events	% Gated	% Total	X Mean	X Geo Mean	Y Mean	Y Geo Mean
UL	385	1.35	1.05	6.65	5.97	51.28	39.16
UR	510	1.78	1.40	32.56	30.04	41.79	37.00
LL	1301	4.55	3.56	6.00	4.52	11.15	9.54
LR	26426	92.33	72.41	34.22	32.32	7.78	7.14

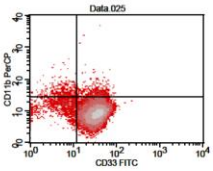

Quadrant Statistics

File: Data.025
Sample ID:
Tube: Untitled
Acquisition Date: 26-Sep-24
Gated Events: 34492
X Parameter: CD33 FITC (Log)
Quad Location: 12, 24
Log Data Units: Linear Values
Patient ID:
Panel: Untitled Acquisition Tube List
Gate: G1
Total Events: 42180
Y Parameter: CD11b PerCP (Log)

Quad	Events	% Gated	% Total	X Mean	X Geo Mean	Y Mean	Y Geo Mean
UL	547	1.59	1.30	6.27	5.61	44.07	39.29
UR	822	2.38	1.95	34.77	32.10	47.66	35.48
LL	1154	3.35	2.74	5.87	4.51	13.07	11.70
LR	31969	92.69	75.79	34.59	32.79	8.83	8.19

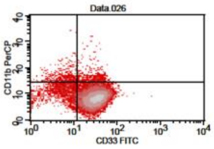

Quadrant Statistics

File: Data.026
Sample ID:
Tube: Untitled
Acquisition Date: 26-Sep-24
Gated Events: 39321
X Parameter: CD33 FITC (Log)
Quad Location: 12, 24
Log Data Units: Linear Values
Patient ID:
Panel: Untitled Acquisition Tube List
Gate: G1
Total Events: 49065
Y Parameter: CD11b PerCP (Log)

Quad	Events	% Gated	% Total	X Mean	X Geo Mean	Y Mean	Y Geo Mean
UL	590	1.50	1.20	6.40	5.76	56.80	42.26
UR	685	1.74	1.40	30.52	27.83	55.05	39.41
LL	1745	4.44	3.56	6.26	4.91	11.30	9.76
LR	36301	92.32	73.99	32.77	30.97	7.65	7.04

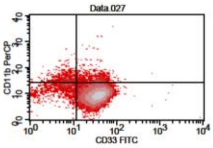

Quadrant Statistics

File: Data.027
Sample ID:
Tube: Untitled
Acquisition Date: 26-Sep-24
Gated Events: 38221
X Parameter: CD33 FITC (Log)
Quad Location: 12, 24
Log Data Units: Linear Values
Patient ID:
Panel: Untitled Acquisition Tube List
Gate: G1
Total Events: 47130
Y Parameter: CD11b PerCP (Log)

Quad	Events	% Gated	% Total	X Mean	X Geo Mean	Y Mean	Y Geo Mean
UL	846	2.21	1.80	6.87	6.36	43.81	39.24
UR	1198	3.13	2.54	35.34	31.59	46.76	36.55
LL	1009	2.64	2.14	6.42	5.33	14.42	12.91
LR	35168	92.01	74.62	36.68	34.41	9.64	8.97

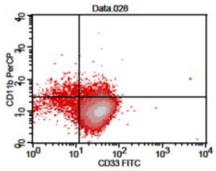

Quadrant Statistics

File: Data.028
Sample ID:
Tube: Untitled
Acquisition Date: 26-Sep-24
Gated Events: 41486
X Parameter: CD33 FITC (Log)
Quad Location: 12, 24
Log Data Units: Linear Values
Patient ID:
Panel: Untitled Acquisition Tube List
Gate: G1
Total Events: 50000
Y Parameter: CD11b PerCP (Log)

Quad	Events	% Gated	% Total	X Mean	X Geo Mean	Y Mean	Y Geo Mean
UL	742	1.79	1.48	6.39	5.71	45.82	40.73
UR	1675	4.04	3.35	37.89	32.66	39.34	34.95
LL	1015	2.45	2.03	6.22	4.85	14.43	13.12
LR	38054	91.73	76.11	34.75	32.78	10.62	9.88

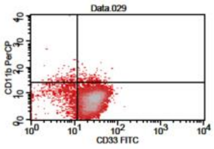

Quadrant Statistics

File: Data.029
Sample ID:
Tube: Untitled
Acquisition Date: 26-Sep-24
Gated Events: 17630
X Parameter: CD33 FITC (Log)
Quad Location: 12, 24
Log Data Units: Linear Values
Patient ID:
Panel: Untitled Acquisition Tube List
Gate: G1
Total Events: 24990
Y Parameter: CD11b PerCP (Log)

Quad	Events	% Gated	% Total	X Mean	X Geo Mean	Y Mean	Y Geo Mean
UL	188	1.07	0.75	6.46	5.91	92.92	38.60
UR	191	1.08	0.76	25.18	23.24	41.41	37.41
LL	1252	7.10	5.01	6.63	4.95	7.96	5.50
LR	15999	90.75	64.02	27.80	26.16	6.54	5.58

ATRA+FAs (2)

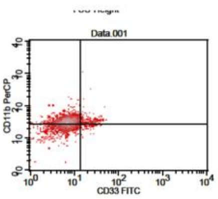

Quadrant Statistics

File: Data.001
Sample ID:
Tube: Untitled
Acquisition Date: 11-Oct-24
Gated Events: 2599
X Parameter: CD33 FITC (Log)
Quad Location: 13, 25
Log Data Units: Linear Values
Patient ID:
Panel: Untitled Acquisition Tube List
Gate: G1
Total Events: 4740
Y Parameter: CD11b PerCP (Log)

Quad	Events	% Gated	% Total	X Mean	X Geo Mean	Y Mean	Y Geo Mean
UL	1647	63.37	34.75	7.10	6.63	36.41	35.25
UR	224	8.62	4.73	24.41	23.31	42.25	35.65
LL	691	26.59	14.58	5.81	5.17	19.11	18.26
LR	37	1.42	0.78	20.44	19.76	20.90	20.44

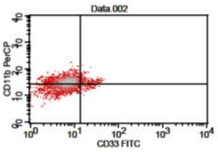

Quadrant Statistics

File: Data.002
Sample ID:
Tube: Untitled
Acquisition Date: 11-Oct-24
Gated Events: 4411
X Parameter: CD33 FITC (Log)
Quad Location: 13, 25
Log Data Units: Linear Values
Patient ID:
Panel: Untitled Acquisition Tube List
Gate: G1
Total Events: 7740
Y Parameter: CD11b PerCP (Log)

Quad	Events	% Gated	% Total	X Mean	X Geo Mean	Y Mean	Y Geo Mean
UL	3077	69.76	39.75	7.18	6.74	37.85	36.68
UR	318	7.21	4.11	24.13	22.87	37.15	35.88
LL	970	21.99	12.53	5.59	4.97	19.43	18.77
LR	46	1.04	0.59	20.18	19.67	21.13	20.87

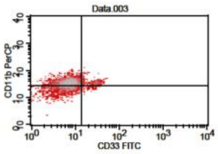

Quadrant Statistics

File: Data.003
Sample ID:
Tube: Untitled
Acquisition Date: 11-Oct-24
Gated Events: 4109
X Parameter: CD33 FITC (Log)
Quad Location: 13, 25
Log Data Units: Linear Values
Patient ID:
Panel: Untitled Acquisition Tube List
Gate: G1
Total Events: 7260
Y Parameter: CD11b PerCP (Log)

Quad	Events	% Gated	% Total	X Mean	X Geo Mean	Y Mean	Y Geo Mean
UL	2875	69.97	39.60	7.15	6.68	38.40	37.26
UR	343	8.35	4.72	24.55	23.22	38.22	36.83
LL	859	20.91	11.83	5.55	4.88	19.13	18.44
LR	32	0.78	0.44	20.00	19.49	21.81	21.60

Figure S5. Gating strategy for flow cytometry experiments.

A. FSC/SSC density plots with a broad gate (R1) set to encompass differentiating cell population (CD11b+ and CD33-) in all tested conditions. B. Single channel staining for the gated population (R1) CD33 (left) and CD11b (right) used for compensation and for background setting. WT HL-60 cells induced by ATRA (to obtain CD11b which is not present in non-induced cells).

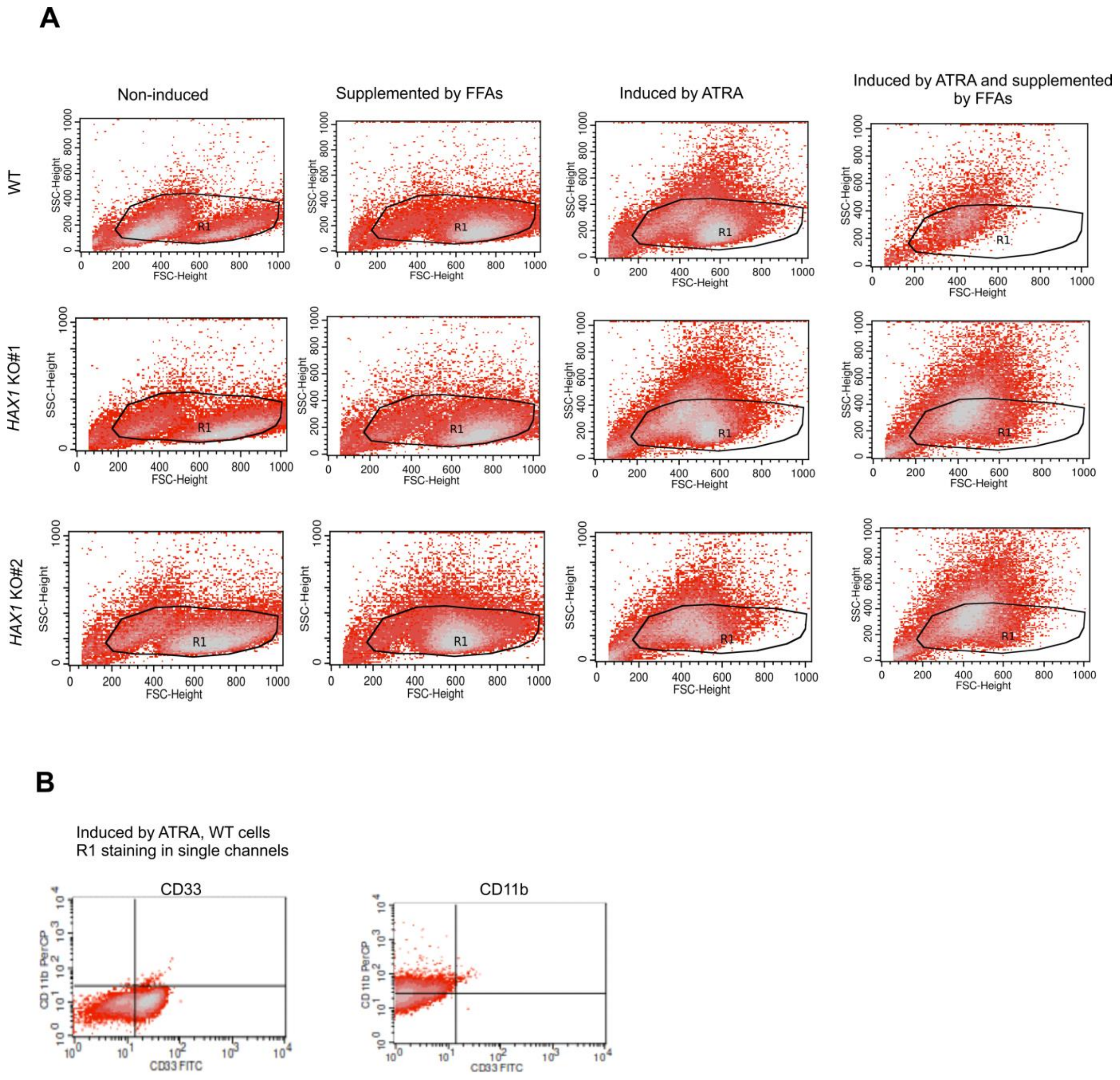

Figure S6. Changes in ATP production rate for mitochondrial respiration and glycolysis

A. Untreated cells, proportion of mito/glycoATP production rate for technical repeats of WT and *HAX1* KO (#1 and #2) cell lines. B. .Cells treated with ATRA+FAs for 24h, proportion of mito/glycoATP production rate for technical repeats of WT and HAX1 KO (#1 and #2) cell lines. C. % of energy production derived from glycolysis (Glyco) and oxidative phosphorylation (Mito) calculated for untreated WT and HAX1 KO (#1 and #2) cell lines. Statistical difference calculated by ANOVA and Tukey, details in Table S1. D. % of energy production derived from glycolysis (Glyco) and oxidative phosphorylation (Mito) calculated for WT and HAX1 KO (#1 and #2) cell lines treated with ATRA+FAs. Statistical difference calculated by ANOVA and Tukey, details in Table S1.

A

Untreated

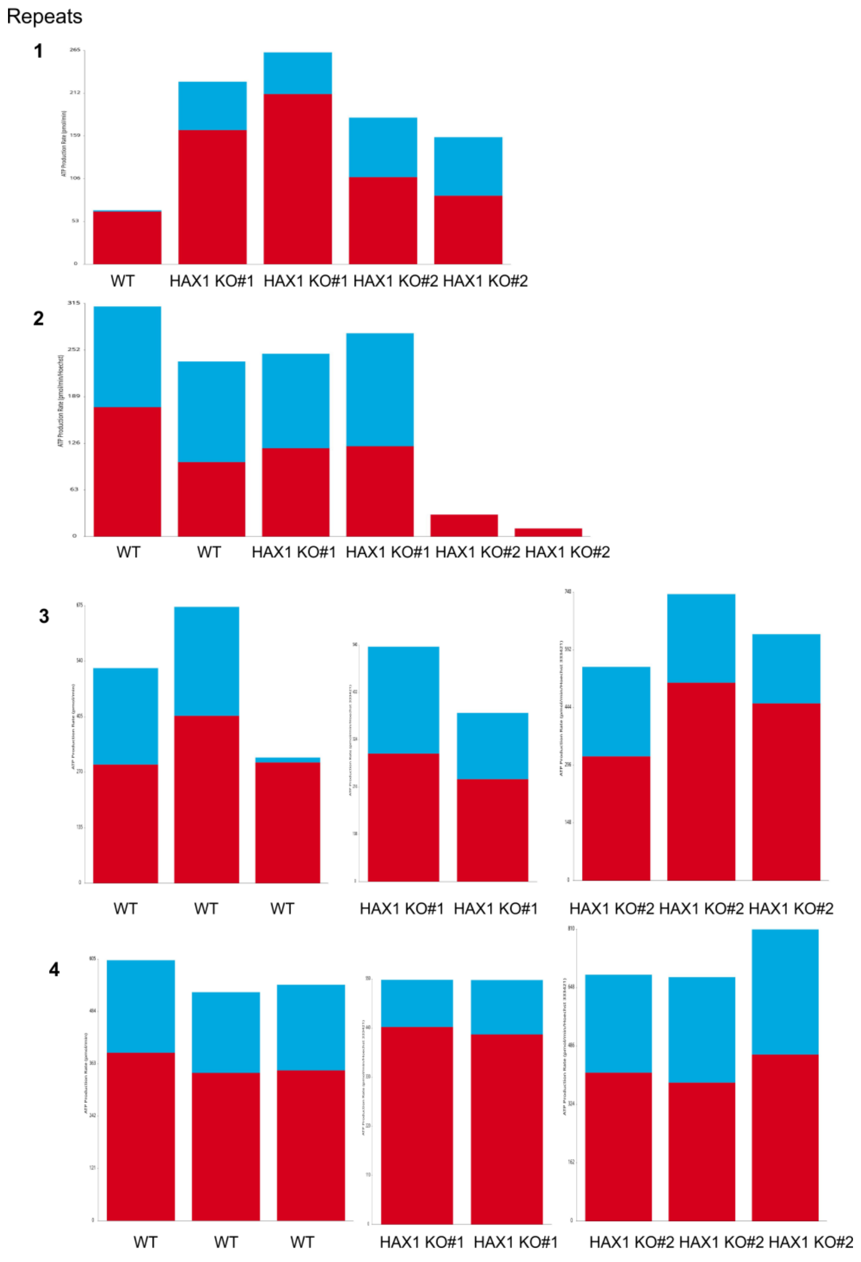

B

24h ATRA+FAs

Reapeat 1

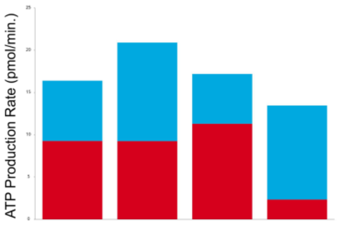

WT

mitoATP Mean 8.94 | Std Dev: 2.89
glycoATP Mean 8.06 | Std. Dev: 3.92

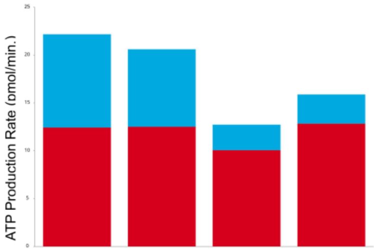

HAX1 KO#1

mitoATP Mean 5.89 | Std Dev: 3.57
glycoATP Mean 11.97 | Std. Dev: 1.28

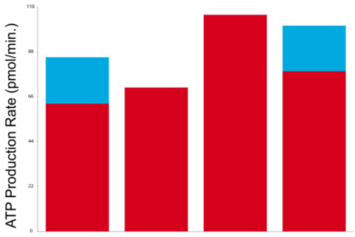

HAX1 KO#2

mitoATP Mean 11.29 | Std Dev: 13.04
glycoATP Mean 79.57 | Std. Dev: 18.97

Reapeat 2

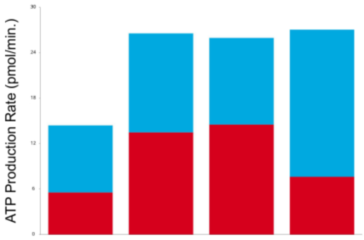

WT

mitoATP Mean 13.20 | Std Dev: 4.50
glycoATP Mean 10.31 | Std. Dev: 4,38

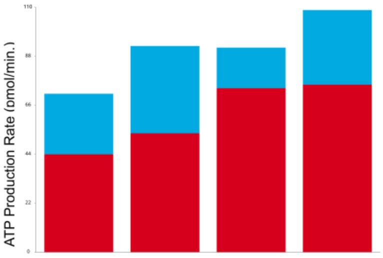

HAX1 KO#1

mitoATP Mean 29.52. | Std Dev: 9.00
glycoATP Mean 61.66 | Std. Dev: 15.39

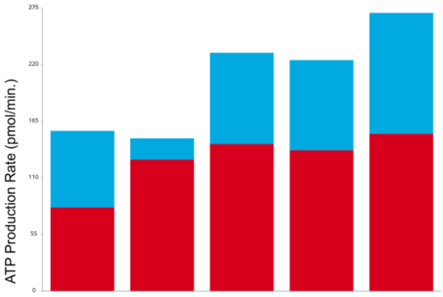

HAX1 KO#2

mitoATP Mean 77.85 | Std Dev: 35.62
glycoATP Mean 128.34 | Std. Dev: 27.82

Figure S7. Statistical analysis of morphological changes in the nuclei of the untreated and ATRA-induced HL-60 cells.

Cells classified as promyelocytes, melocytes/metamyelocytes, band cells and segmented cells. Upper panel: untreated cells, lower panel: 58 µM ATRA-induced cells. Statistical analysis: One-way ANOVA, Tukey, n=7-10, statistical details in Table S1.

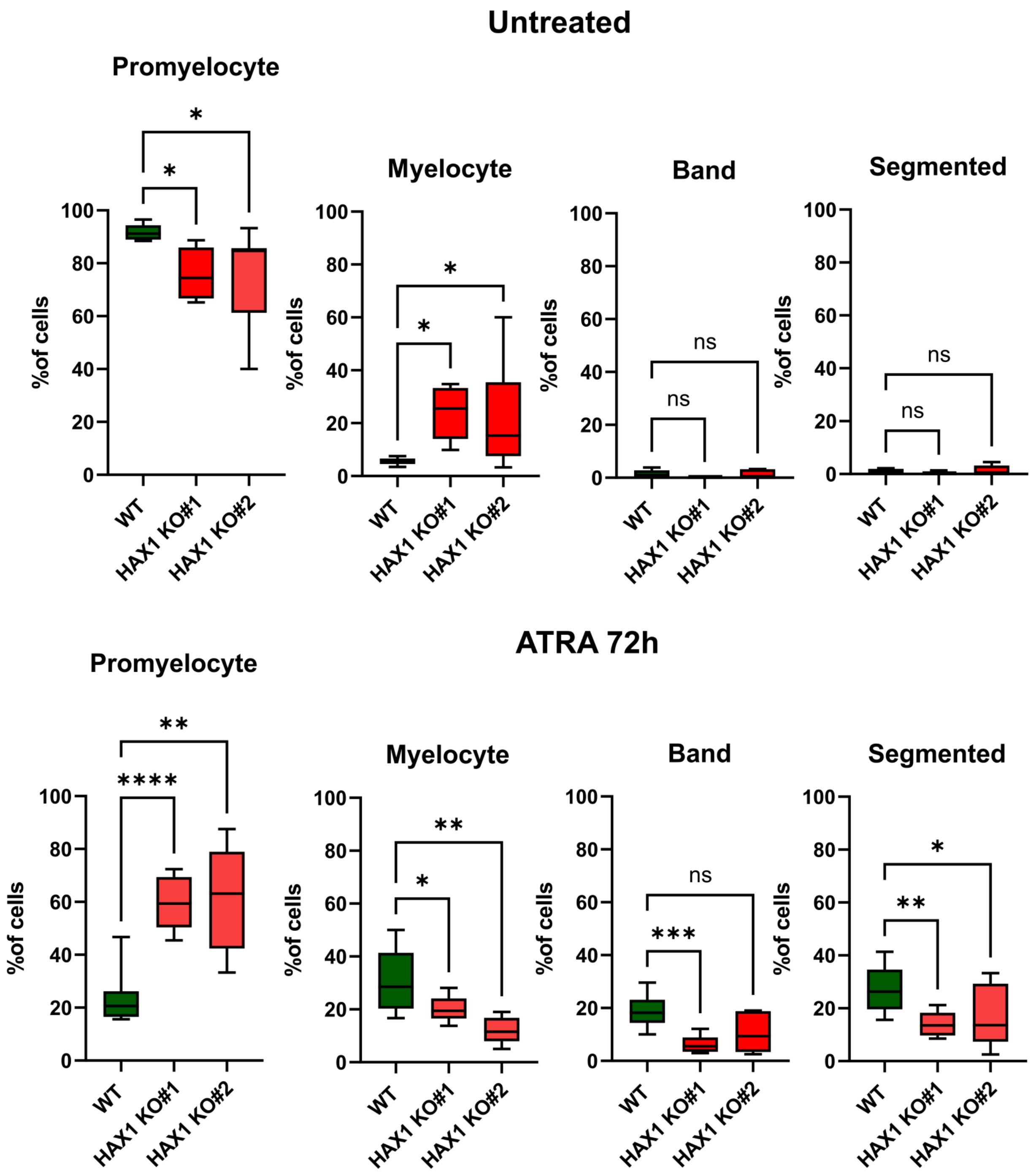

Supplement: Supplementary file 1 — Figures S1-S7 [file 41419_2026_8805_MOESM1_ESM.pdf]
