# Supplementary material for: Granulocyte differentiation arrest in HAX1-deficient cells, demonstrated in a new in vitro model of a certain phenotypic aspects of Kostmann disease, is caused by ineffective lipid droplet autophagy and fatty acids uptake
Source: Cell Death Dis. 2026 May 5;17(1):594. doi: 10.1038/s41419-026-08805-y (PMC13287692; doi:10.1038/s41419-026-08805-y)
Supplement: Supplementary file 2 — Tables S1-S5 [file 41419_2026_8805_MOESM2_ESM.pdf]

Table S1. Parameters of all One-way ANOVA and Tukey analyses in the study

Figure panel	Sample description	Group size (n)	Number of biological replicates	P-value	F (DFn, DFd),
3	Decanoic acid:				F(2,21)=7.379
	WT vs. HAX1 KO#1	9/6	3/2	0.0315	
	WT vs. HAX1 KO#2,	9/9	3/3	0.3123	
	α-linolenic acid:				F(2,24)=8.400
	WT vs. *HAX1* KO#1	9/9	3/3	0.0025	
	WT vs. *HAX1* KO#2,	9/9	3/3	0.0039	
	Lauric acid:				F(2,24)=48.27
	WT vs. *HAX1* KO#1	9/9	3/3	<0.0001	
	WT vs. *HAX1* KO#2,	9/9	3/3	0.0001	
	Arachidic acid:				F(2,24)=5.084
	WT vs. *HAX1* KO#1	9/9	3/3	0.2592	
	WT vs. *HAX1* KO#2,	9/9	3/3	0.1734	
	Adrenic acid:				F(2,24)=127.2
	WT vs. *HAX1* KO#1	9/9	3/3	<0.0001	
	WT vs. *HAX1* KO#2,	9/9	3/3	<0.0001	
	Erucic acid:				F(2,18)=157.6
	WT vs. *HAX1* KO#1	7/5	2/2	0.0898	
	WT vs. *HAX1* KO#2,	7/9	2/3	<0.0001	
	DHGL acid:				F(2,24)=28.76
	WT vs. *HAX1* KO#1	9	3/3	<0.0001	
	WT vs. *HAX1* KO#2,	9	3/3	<0.0001	
	EPA:				F(2,24)=43.31
	WT vs. *HAX1* KO#1	9	3/3	0.0006	
	WT vs. *HAX1* KO#2,	9	3/3	<0.0001	
	Nervonic acid:				F(2,24)=120.8
	WT vs. *HAX1* KO#1	9	3/3	0.2150	
	WT vs. *HAX1* KO#2,	9	3/3	<0.0001	
	Linoleic acid:				F(2,24)=100.7
	WT vs. *HAX1* KO#1	9	3/3	0.6728	
	WT vs. *HAX1* KO#2,	9	3/3	<0.0001	
	Arachidonic acid:				F(2,24)=40.59
	WT vs. *HAX1* KO#1	9	3/3	0.0057	
	WT vs. *HAX1* KO#2,	9	3/3	<0.0001	
	Myristic acid:				F(2,24)=262.6
	WT vs. *HAX1* KO#1	9	3/3	0.0789	
	WT vs. *HAX1* KO#2,	9	3/3	<0.0001	
	Palmitoleic acid:				F(2,24)=114.8
	WT vs. *HAX1* KO#1	9	3/3	0.9686	
	WT vs. *HAX1* KO#2,	9	3/3	<0.0001	
	DHA:				F(2,24)=98.83
	WT vs. *HAX1* KO#1	9	3/3	<0.0001	
	WT vs. *HAX1* KO#2,	9	3/3	<0.0001	
	Trans-vaccenic acid:				F(2,24)=100.2
	WT vs. *HAX1* KO#1	9	3/3	0.0597	
	WT vs. *HAX1* KO#2,	9	3/3	<0.0001	
	Oleic acid:				F(2,24)=21.26
	WT vs. *HAX1* KO#1	9	3/3	0.9937	
	WT vs. *HAX1* KO#2	9	3/3	<0.0001	
4A	Acetoacetate:		3		F (2, 21) =
	WT vs. *HAX1* KO#1	8/8		0.0431	5.467
	WT vs. *HAX1* KO#2,	8/8		0.0152	
	β-hydroxybutyrate:		3		F (2, 21) =
	WT vs. *HAX1* KO#1	9/8		0,7088	0,3697
	WT vs. *HAX1* KO#2	9/7		0,7949	
	Fatty acids:		3		F (2, 20) =
	WT vs. *HAX1* KO#1	6/8		0.0305	9.824
	WT vs. *HAX1* KO#2	6/9		0.0007	
4C	Urea:		3		F (2, 31) =
	WT vs. *HAX1* KO#1	12/12		0.0613	9.497
	WT vs. *HAX1* KO#2	12/10		0.004	
	Aspartate:		3		F (5, 39) =

	WT vs. *HAX1* KO#1	9/9		0.0057	291.2
	WT vs. *HAX1* KO#2	9/9		<0.0001	
	Lactate:		3		F (2, 20) =
	WT vs. *HAX1* KO#1	8/8		<0.0001	40.17
	WT vs. *HAX1* KO#2	8/7		<0.0001	
6B	LD numbers:				
	Untreated:		8		F (5, 196) =
	WT vs. *HAX1* KO#1	51/52		<0.0001	285.4
	WT vs. *HAX1* KO#2	51/51		<0.0001	
	HAX1 KO#1 vs. *HAX1* KO#2	52/51		<0.0001	
	bafilomycin		5		F (5, 174) =
	WT vs. *HAX1* KO#1	30/30		0.6361	314.2
	WT vs. *HAX1* KO#2	30/30		0.9994	
	HAX1 KO#1 vs. *HAX1* KO#2	30/30		0.4136	
	WT untreated vs. WT bafilomycin	30/30		<0.0001	
	E64D		3		F (5, 196) =
	WT vs. *HAX1* KO#1	16/16		0.9992	285.4
	WT vs. *HAX1* KO#2	16/16		>0.9999	
	HAX1 KO#1 vs. *HAX1* KO#2	16/16		0.9926	
	WT untreated vs. WT E64D	51/16		<0.0001	
6C	LC3(autophagosomes):				
	Untreated:				F (5, 379) =
	WT vs. *HAX1* KO#1	61/82	10/13	<0.0001	3446
	WT vs. *HAX1* KO#2	61/66	10/11	<0.0001	
	bafilomycin				F (5, 379) =
	WT vs. *HAX1* KO#1	56/62	9/10	<0.0001	3446
	WT vs. *HAX1* KO#2	56/58	9/10	<0.0001	
	E64D				F (5, 174) =
	WT vs. *HAX1* KO#1	30/30	5/5	<0.0001	1264
	WT vs. *HAX1* KO#2	30/30	5/5	<0.0001	
6D	mCherry/GFP ratio (untreated vs. starved)				F (5, 176) =
	WT vs. WT 2h				7.264
	HAX1 KO#1 vs. *HAX1* KO#1 2h	34/31	3	<0.0001	
	HAX1 KO#1 vs. *HAX1* KO#1 2h	21/23	2	0.7483	
		36/33	3	0.9778	
7C	%CD11b+ cells				
	Untreated				F (2, 42) =
	WT vs. *HAX1* KO#1	15/14	4/3	<0.0001	31.19
	WT vs. *HAX1* KO#2	15/16	4/4	<0.0001	
	HAX1 KO#1 vs. *HAX1* KO#2	14/16	3/4	0.0870	
	Low DMSO (non-induced control)				F (2, 13) =
	WT vs. *HAX1* KO#1	5/5	1/1	0.0010	13,16
	WT vs. *HAX1* KO#2	5/6	1/1	0.0035	
	HAX1 KO#1 vs. *HAX1* KO#2	5/6	1/1	0.6318	
	Free FAs				F (2, 20) =
	WT vs. *HAX1* KO#1	8/10	2/1	0.0341	3.735
	WT vs. *HAX1* KO#2	8/5	2/1	0.5952	
	HAX1 KO#1 vs. *HAX1* KO#2	10/5	1/1	0.3967	
	ATRA				F (2, 46) =
	WT vs. *HAX1* KO#1	18/12	4/3	0.0001	20.26
	WT vs. *HAX1* KO#2	18/19	4/5	<0.0001	
	HAX1 KO#1 vs. *HAX1* KO#2	12/19	3/5	0.7514	
	ATRA+FAs				F (2, 25) =
	WT vs. *HAX1* KO#1	12/6	2/1	0.0005	50.96
	WT vs. *HAX1* KO#2	12/9	2/2	<0.0001	
	HAX1 KO#1 vs. *HAX1* KO#2	6/9	1/2	0.0002	
8C	Untreated		3		F(2,36)=3.583
	WT vs. *HAX1* KO#1	12/13		0.0387	
	WT vs. *HAX1* KO#2	12/14		0.0541	
	ATRA		3		F(2,38)=14.49
	WT vs. *HAX1* KO#1	14/13		0.0001	
	WT vs. *HAX1* KO#2	14/14		<0.0001	
	ATRA+PMA		3		F(2,40)=42.77
	WT vs. *HAX1* KO#1	15/14		<0.0001	
	WT vs. *HAX1* KO#2	15/14		<0.0001	
8D	Untreated		4		F(5, 46)=11.93
	WTglyco vs. WTmito	9		0.0013	

	HAX1 KO#1glyco vs. *HAX1* KO#1mito	8		<0.0001	
	HAX1 KO#2glyco vs. *HAX1* KO#2mito	9		0.0294	
	ATRA+FAs		2		
	WTglyco vs. WTmito	8		0.7006	F(5,46)=14.54
	HAX1 KO#1glyco vs. *HAX1* KO#1mito	8		0.0002	
	HAX1 KO#2glyco vs. *HAX1* KO#2mito	9		<0.0001	
S7	**Untreated**		1		
	Promyelocyte				F(2,19)=5.040
	WT vs. *HAX1* KO#1	8/7		0.0290	
	WT vs. *HAX1* KO#2	8/7		0.0224	
	Myelocyte/Metamyelocyte				F(2,19)=5.200
	WT vs. *HAX1* KO#1	8/7		0.0170	
	WT vs. *HAX1* KO#2	8/7		0.0332	
	Band cell				F(2,19)=2.477
	WT vs. *HAX1* KO#1	8/7		0.1101	
	WT vs. *HAX1* KO#2	8/7		0.9988	
	Segmented				F(2,19)=1.994
	WT vs. *HAX1* KO#1	8/7		0.3449	
	WT vs. *HAX1* KO#2	8/7		0.6962	
	**ATRA**		1		
	Promyelocyte				F(1.36,12.19)=
	WT vs. *HAX1* KO#1	10/10		<0.0001	17.48
	WT vs. *HAX1* KO#2	10/10		0.0033	
	Myelocyte/Metamyelocyte				F(1.45,13.01)=
	WT vs. *HAX1* KO#1	10/10		0.0243	13.50
	WT vs. *HAX1* KO#2	10/10		0.0069	
	Band cell				F(1.4,12.57)=
	WT vs. *HAX1* KO#1	10/10		0.0002	12.69
	WT vs. *HAX1* KO#2	10/10		0.0684	
	Segmented				F(1.62,14.55)=
	WT vs. *HAX1* KO#1	10/10		0.0011	8.325
	WT vs. *HAX1* KO#2	10/10		0.0267	

Table S2.Statistical analysis of the fatty acid uptake dynamics (Wilcoxon rank-sum test) .

A. Untreated cells

	group A	group B	cox.statistic	cox.pvalue
1	WT_UNTR_0	HAX1 KO#1_UNTR_0	18	0.0503085150143974
2	WT_UNTR_0	HAX1 KO#2_UNTR_0	11	0.0103254389457132
3	WT_UNTR_20	HAX1 KO#1_UNTR_20	9	0.00399012751953928
4	WT_UNTR_20	HAX1 KO#2_UNTR_20	0	0.000384567345635064
5	WT_UNTR_60	HAX1 KO#1_UNTR_60	9	0.00399012751953928
6	WT_UNTR_60	HAX1 KO#2_UNTR_60	0	0.000358194289171574

B. Cells after insulin treatment

	group A	group B	cox.statistic	cox.pvalue
1	WT_INS_0	HAX1 KO#1_INS_0	18	0.0503085150143974
2	WT_INS_0	HAX1 KO#2_INS_0	18	0.0503085150143974
3	WT_INS_20	HAX1 KO#1_INS_20	4	0.000493624023035788
4	WT_INS_20	HAX1 KO#2_INS_20	0	4.11353352529823e-05
5	WT_INS_60	HAX1 KO#1_INS_60	5	0.000781571369806664
6	WT_INS_60	HAX1 KO#2_INS_60	0	4.11353352529823e-05

Table S3. Lipid droplet and autophagosome analysis. Numeral table showing the mean value for each biological replicate, number of technical repeats and cell number in the replicate (where appropriate), SD and SEM.

Sample description	n (in one replicate)	Cell number (in one replicate)	Mean (per cell)	SD	SEM
LD numbers:<br>**Untreated:**<br>WT					
	6	340	0.652200945	0.135039252	0.055129544
	6	334	0.725232062	0.173429727	0.07080239
	6	277	0.722015905	0.0917381	0.037451923
	6	324	0.613487085	0.160859395	0.065670573
	6	330	0.582849932	0.15133829	0.061783598
	6	341	0.583081206	0.112814497	0.046056325
	6	340	0.574228774	0.149921348	0.061205134
	9	476	0.588498334	0.054664807	0.018221602
HAX1 KO#1					
	6	306	1.215125811	0.083919788	0.03426011
	6	312	1.207396978	0.054077497	0.022077046
	6	316	1.261976641	0.074026996	0.030221395
	6	301	1.373566434	0.166133576	0.067823749
	6	288	1.217329932	0.037476769	0.015299827
	6	277	1.304990785	0.121654448	0.049665221
	6	278	1.392802772	0.136836018	0.05586307
	10	516	1.272304098	0.11291978	0.03570837
HAX1 KO#2					
	6	290	1.535904413	0.080674388	0.032935181
	6	281	1.578808923	0.197251608	0.080527632
	6	255	1.557201073	0.076753503	0.031334486
	6	267	1.561326475	0.254348391	0.103837296
	6	253	1.479483331	0.073384973	0.02995929
	6	245	1.571026427	0.20462945	0.083539623
	6	256	1.575642261	0.170136024	0.069457741
	9	400	1.456943828	0.151045693	0.050348564
**Bafilomycin**<br>WT					
	6	330	1.546840659	0.068394511	0.027921942
	6	312	1.705937023	0.059792806	0.024410311
	6	316	1.629544109	0.128473332	0.052449018
	6	314	1.616355386	0.07618722	0.031103302
	6	285	1.717922754	0.070208128	0.028662348
HAX1 KO#1					
	6	269	1.650541178	0.110899152	0.045274389
	6	260	1.733732623	0.146100795	0.0596454
	6	221	1.665139667	0.178592544	0.072910101
	6	296	1.713412224	0.117269953	0.047875258
	6	286	1.696243941	0.114235414	0.046636412
HAX1 KO#2					
	6	273	1.733680565	0.051632835	0.021079017
	6	213	1.555012762	0.043584803	0.017793421
	6	246	1.741573448	0.099786758	0.040737773
	6	248	1.605719704	0.120450232	0.049173601
	6	240	1.526818205	0.147778808	0.060330446
**E64D**<br>WT					
	6	267	1.354542439	0.146743723	0.059907874
	6	278	1.250849601	0.056156817	0.022925925
	4	182	1.301675725	0.038830533	0.019415267
HAX1 KO#1					
	6	299	1.255982714	0.055967734	0.022848732
	6	294	1.294417644	0.031522659	0.012869072
	4	194	1.31879085	0.029580128	0.014790064
HAX1 KO#2					
	6	264	1.243327603	0.052504633	0.021434927

	6	279	1.314811827	0.048141039	0.019653497
	4	181	1.410645282	0.094857231	0.047428616
LC3 puncta (autophagosomes):					
**Untreated:**					
WT	6	130	3.143568692	0.207162929	0.084573912
	6	149	3.489577347	0.255914849	0.1044768
	6	152	3.472692414	0.137745341	0.0562343
	6	156	3.57477196	0.131448675	0.053663697
	6	158	3.382473312	0.181139742	0.07394999
	6	138	3.290931677	0.193661927	0.079062151
	6	112	3.381178775	0.175884691	0.071804624
	6	157	3.307450867	0.269584066	0.110057234
	6	168	3.355958227	0.307688136	0.125613155
	7	187	3.4173667143	0.299705921	0.11327819
HAX1 KO#1					
	6	64	6.492712843	0.232960036	0.095105537
	6	71	5.895138889	0.450620072	0.183964874
	6	86	6.23754085	0.18227191	0.074412196
	6	125	5.912803566	0.396230171	0.16176029
	6	81	5.999928176	0.570026318	0.23271227
	6	72	5.867327117	0.286355149	0.116904
	6	115	5.845095183	0.388810264	0.158731125
	6	90	5.747616514	0.311956036	0.127355518
	6	106	5.901394994	0.225758131	0.092165371
	7	106	6.067738475	0.559494041	0.21146887
	6	109	6.185650306	0.533826493	0.217933753
	6	89	5.665780593	0.28445977	0.116130215
	9	136	6.173321123	0.449633119	0.149877706
HAX1 KO#2					
	6	82	7.466956654	0.261903228	0.106921545
	6	97	7.197031039	0.286031505	0.116771873
	6	72	7.423354097	0.253053862	0.103308807
	6	106	7.126214064	0.262164611	0.107028254
	6	87	7.250676937	0.454539537	0.185564989
	6	73	7.041545954	0.580490717	0.236984343
	6	91	7.505377005	0.534858945	0.21835525
	6	92	7.235430283	0.415833934	0.169763493
	6	109	7.123463274	0.415070517	0.169451829
	6	125	7.279575517	0.387421669	0.158164234
	6	121	7.060540554	0.749102531	0.305819828
**Bafilomycin**					
WT	6	93	8.00376462	0.68784153	0.280810129
	6	91	7.835443759	0.492635297	0.201117518
	6	78	7.891362877	0.533647488	0.217860675
	6	91	8.160643292	0.424875391	0.173454652
	6	101	8.176256614	0.289482472	0.118180724
	6	105	8.194212963	0.427308921	0.174448137
	6	99	8.178666472	0.440256077	0.179733791
	6	98	8.23211675	0.264140516	0.107834914
	8	82	8.139961081	0.477734615	0.168904693
HAX1 KO#1					
	6	185	11.959655	0.665892435	0.271849448
	6	166	12.3480893	0.925290861	0.377748412
	6	117	12.13227347	1.013799779	0.413882026
	6	159	12.51208031	0.919624771	0.375435241
	6	166	11.94305578	0.64813758	0.264601059
	6	145	12.17147165	1.109127906	0.452799571
	6	153	12.43117898	0.899302292	0.367138623
	6	153	12.12270334	0.760732	0.310567539
	6	94	11.53805556	0.602873136	0.213147841
	7	135	12.52011778	0.941134956	0.355715578
HAX1 KO#2					
	6	112	14.92426383	0.920412536	0.375756844
	6	127	14.89707368	0.884819686	0.361226124
	6	87	15.04021904	1.135669068	0.463634956
	6	71	15.14824435	0.82948127	0.33863431

	6	87	15.44080197	0.583267153	0.238117818
	6	89	15.25154486	0.625462971	0.255344189
	6	56	15.67013889	0.6965212	0.284353589
	6	109	15.26339031	0.614782948	0.250984087
	6	136	15.15139167	0.909908193	0.321701127
	4	121	14.31094114	0.457187068	0.228593534
**E64D**					
WT	6	303	7.80939508	0.262101702	0.107002572
	6	302	8.013852408	0.25195557	0.102860431
	6	297	7.568037421	0.3915147	0.159835207
	6	314	8.176473521	0.440865756	0.179982691
	6	311	7.476354026	0.514194744	0.209919125
HAX1 KO#1					
	6	295	9.84017045	0.655514419	0.267612641
	6	275	10.22347173	0.652619227	0.266430684
	6	286	9.521425391	0.73879159	0.301610404
	6	324	10.47667815	0.678422207	0.276964706
	6	270	10.08036006	0.427188018	0.174398778
HAX1 KO#2					
	6	304	14.69719629	0.442123121	0.180496008
	6	280	15.80509858	0.792018707	0.323340283
	6	280	15.30495439	1.042003312	0.425396071
	6	336	14.34363667	1.749050154	0.714046735
	6	263	15.17778767	1.247122008	0.509135428
mCherry/GFP ratio (untreated vs. starved)					
0h					
WT			(normalized)		
	9		1	0.166466248	0.055488749
	11		1	0.407734292	0.122936515
	15		1	0.138925488	0.035870407
HAX1 KO#1					
	10		1.07798112	0.151648947	0.047955608
	15		1.002673888	0.106026032	0.027375804
HAX1 KO#2					
	11		1.227304702	0.138812159	0.041853441
	11		1.059652854	0.190072104	0.057308896
	14		1.168721379	0.204339195	0.054611947
2h starved					
WT					
	11		1.153546519	0.116458273	0.035113491
	7		1.339297857	0.504584052	0.152137816
	13		1.168173244	0.081539408	0.022614963
HAX1 KO#1					
	10		1.110155026	0.120452526	0.038090433
	13		1.10837387	0.110575523	0.030668132
HAX1 KO#2					
	11		1.219103519	0.199329556	0.060100122
	10		1.118398817	0.404671358	0.122013005
	13		1.174072093	0.120820359	0.033509538

Table S4.The plasma profiles used in QSM analysis

overnight fast state	
Glucose 5.8 mM	Isoleucine 0.06 mM
Fatty acids 0.5 mM	β-hydroxybutyrate 0.08 mM
Lactate 0.8 mM	Acetoacetate 0.04 mM
Valine 0.2 mM	Catecholamines 0.75 nM
Leucin 0.15 mM	Insulin 100 pM

Table S5. Description of the antibodies used in the research

**Epitope**	**Company**	**Cat.#**	**Clone**	**Dilution**	**Application**
**Primary**					
FLAG	ThermoFisher Scientific	MA1-91878	FG4R	1:300	IP
HAX1	ThermoFisher Scientific	PA5-27592		1:500	WB
HAX1	Proteintech	11266-1-AP		1:500	IF
Spot-Tag VHH Nanobody conjugated to ATTO594	Chromotek	eba594		1:1000	FRET
LC3A/B	Cell Signaling	12741	D3U4C	1:200	IF
CD11b	ThermoFisher Scientific	46-0118-42	ICRF44 PERCPEF 710	5ul/test	Flow cytometry
CD33 CoraLite® Plus 488 Anti-Human CD33	Proteintech	CL488-65272	WM53	0,33ul/test	Flow cytometry
**Secondary**					
AlexaFluor 488	Cell Signaling	4412S		1:1000	IF
