# Supplementary figures and images for: Granulocyte differentiation arrest in HAX1-deficient cells, demonstrated in a new in vitro model of a certain phenotypic aspects of Kostmann disease, is caused by ineffective lipid droplet autophagy and fatty acids uptake
Source: Cell Death Dis. 2026 May 5;17(1):594. doi: 10.1038/s41419-026-08805-y (PMC13287692; doi:10.1038/s41419-026-08805-y)

Uncropped images

Figure 1D:

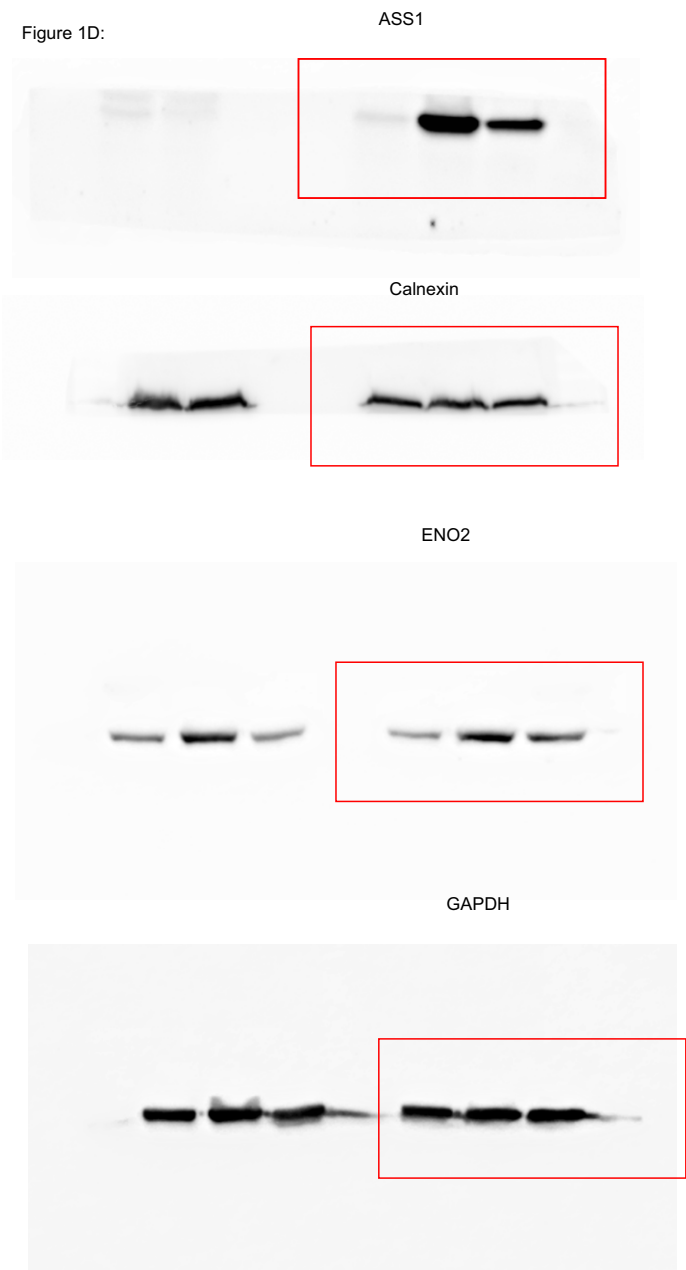

Figure 5E

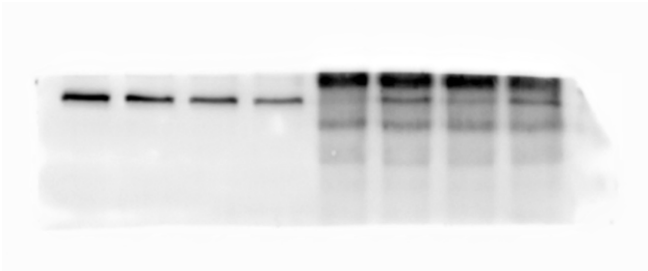

Figure S2

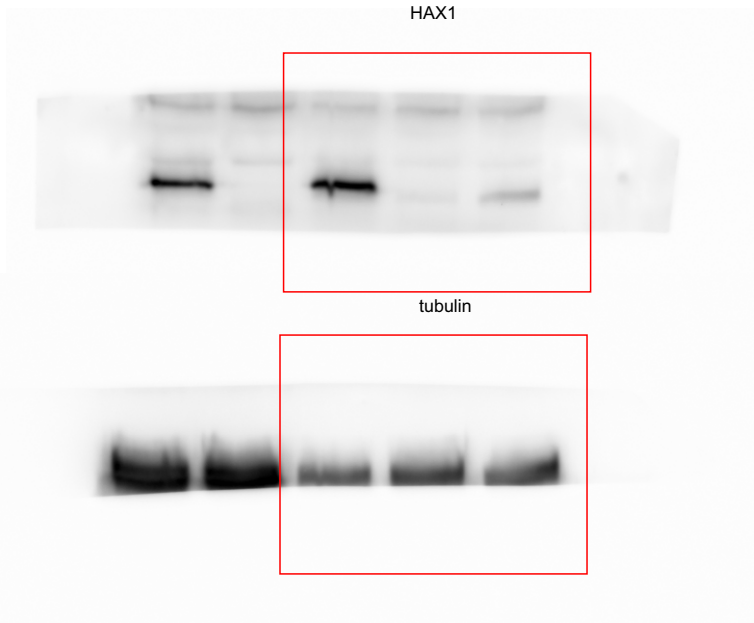

Supplement: Supplementary file 3 — Uncropped WB [file 41419_2026_8805_MOESM3_ESM.pdf]
