# Supplementary material for: Granulocyte differentiation arrest in HAX1-deficient cells, demonstrated in a new in vitro model of a certain phenotypic aspects of Kostmann disease, is caused by ineffective lipid droplet autophagy and fatty acids uptake
Source: Cell Death Dis. 2026 May 5;17(1):594. doi: 10.1038/s41419-026-08805-y (PMC13287692; doi:10.1038/s41419-026-08805-y)
Supplement: Supplementary file 4 — Supplementary files legend [file 41419_2026_8805_MOESM4_ESM.pdf]

File S1. Quantitative mass spectrometry results of HL60 WT and *HAX1* KO#2 cells, each cell line in four replicates.

File S2. Quantitative mass spectrometry data analyzed with DAVID Annotation tool (DAVID Knowledgebase v2025_1) and Enrichr (Ma'ayan Laboratory, Computational Systems Biology).

File S3. QSM DataAnalysis Report (Doppelganger Biosystem GmbH).

File S4. QSM DataAnalysis, statistical details.

File S5. Metabolic mass spectrometry results of HL60 WT and *HAX1* KO cells (#1 and #2) for the fatty acid panel.
